# Ternary Inclusion Co‐Crystals for Efficient Photothermal Conversion and Solar‐Driven Water Evaporation

**DOI:** 10.1002/advs.202500050

**Published:** 2025-04-29

**Authors:** Ruotong Wang, Yi Su, Zhiyu Xiao, Tongtong Wang, Kun Liu, Zhihao Gong, Jiabin Wu, Junyi Chen, Zhixue Liu, Jingjing Li, Yu‐Hui Zhang, Lu Wang, Bin Li, Xiaotao Zhang, Chunju Li

**Affiliations:** ^1^ Academy of Interdisciplinary Studies on Intelligent Molecules Tianjin Key Laboratory of Structure and Performance for Functional Molecules College of Chemistry Tianjin Normal University Tianjin 300387 P. R. China; ^2^ Key Laboratory of Organic Integrated Circuit Ministry of Education & Tianjin Key Laboratory of Molecular Optoelectronic Sciences Department of Chemistry Institute of Molecular Aggregation Science Tianjin University Tianjin 300072 P. R. China; ^3^ College of Science & College of Material Science and Art Design Inner Mongolia Agricultural University Hohhot 010018 P. R. China

**Keywords:** host–guest chemistry, macrocycle, organic co‐crystal, photothermal conversion, solar‐driven water evaporation

## Abstract

Organic co‐crystal engineering offers a convenient and efficient platform for preparing photothermal conversion (PTC) materials. However, current donor–acceptor (D–A) co‐crystals generally have medium photothermal performance. Here, an inclusion co‐crystal strategy is presented, i.e., host–guest encapsulation of small acceptor inside donor‐type macrocycle's cavity, to enhance PTC efficiency through the promotion of D–A binding. A naphthyl‐sidewall Tröger's base (TB[2]) molecular box donor is elaborately designed, which can encapsulate electron‐deficient 7,7,8,8‐tetracyanoquinodimethane (TCNQ) to form a 1:2 ternary inclusion charge‐transfer (CT) co‐crystal via the synergism of multiple noncovalent forces. Under 808 nm laser irradiation (0.7 W cm^−2^), the PTC efficiency of co‐crystals is as high as 94.3%. The co‐crystals are further introduced into the porous polymer of polyurethane (PU) to prepare an interfacial evaporator (TB‐TCNQ@PU) for solar‐driven water evaporation. Under 1 Sun irradiation, a high‐water evaporation rate of 1.746 kg m^−2^ h^−1^ and a prominent solar‐to‐vapor efficiency of 93.8% are achieved. This work opens new avenues for the efficient PTC materials.

## Graphical abstract

An inclusion co‐crystal strategy for elevating photothermal conversion efficiency is developed. A Tröger's base molecular box strongly bind two electron‐deficient TCNQ forming 1:2 ternary inclusion charge‐transfer co‐crystals, which displays a high photothermal efficiency of 94.3%. The co‐crystals are introduced into the porous polyurethane to prepare an interfacial heating system for solar‐driven water evaporation and seawater desalination.

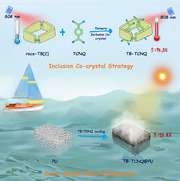

## 1 Introduction

Organic photothermal conversion (PTC) materials have attracted tremendous interests for their great potentials in photothermal therapy (PTT),^[^ ^1^ ^]^ photothermal catalysis,^[^ ^2^ ^]^ latent heat storage,^[^ ^3^ ^]^ and solar evaporation and desalination.^[^ ^4^ ^]^ To maximize the utilization of solar light comprised 53% near‐infrared (NIR) light, two main approaches are employed to boost PTC efficiency: enhancing the NIR absorption to harvest more photoenergy and promoting nonradiative transition processes to avoid energy dissipation.^[^ ^5^ ^]^ At present, typical organic PTC materials embrace indocyanine green dye,^[^ ^6^ ^]^ porphysome,^[^ ^7^ ^]^ organic free radical,^[^ ^8^ ^]^ conjugated organic semiconductor (such as polyaniline,^[^ ^9^ ^]^ and polypyrrole^[^ ^10^ ^]^) and porous organic polymers,^[^ ^11^ ^]^ etc. However, these materials generally involve the complicated design, tedious synthesis and high cost, which have extremely restricted the applications of organic PTC materials to some degree. Therefore, it is essential to develop facile and effective methods for designing and preparing PTC materials.

Organic charge‐transfer (CT) co‐crystals,^[^ ^12^ ^]^ a type of crystalline materials, are formed by exploiting an electron donor (D) and an acceptor (A) in a stoichiometric ratio through the assembly of two components. They could be easily accessible through the mechanochemical preparation, vapor methods and solution‐processing methods, and show unpredicted physicochemical properties such as organic semiconductor,^[^ ^13^ ^]^ ferroelectricity,^[^ ^14^ ^]^ optical waveguide,^[^ ^15^ ^]^ and ambipolar charge transport^[^ ^16^ ^]^ due to the tunable electron energy level and CT nature. In especial, CT co‐crystals display unique electron delocalization from the D to the A, and effectively minimize energy band to achieve a remarkably redshifted NIR absorption,^[^ ^17^ ^]^ providing a significant potential in PTC materials.^[^ ^18^ ^]^ In 2018, Hu and co‐workers pioneered a NIR photothermal co‐crystal using dibenzotetrathiafulvalene (DBTTF) and 1,2,4,5‐tetracyanobenzene (TCNB) with a PTC efficiency of 18.8%.^[^ ^19^ ^]^ Since then, a series of D–A co‐crystal systems based on planar D/A molecules have been fabricated as PTC materials.^[^ ^20^ ^]^

To date, the reported PTC efficiencies of small‐molecule co‐crystals are less than 70% in most cases because of their relatively weak D‐A complexation. Perepichka and co‐workers reported that intermolecular hydrogen bonds in complexes favor CT process and form strong D–A overlaps,^[^ ^21^ ^]^ which effectively suppresses the emission processes and sufficiently converts light energy to heat via nonradiative pathways. Macrocycles having preorganized cavities and multivalent binding sites could engulf suitable guest molecules in high affinities and selectivities.^[^ ^22^ ^]^ We consider that inclusion co‐crystal engineering, i.e., the combination of host–guest chemistry and co‐crystal engineering, would be a powerful strategy to boost PTC performance through the encapsulation of D (or A) guests by A (or D) edged macrocycles. Compared with planar small‐molecule co‐crystals, inclusion co‐crystals possess the following advantages: 1) the encapsulation of guests by macrocycles would significantly enhance the D–A interactions by the synergism of multiple noncovalent forces; 2) the polygonal skeleton and tunable cavity size of macrocycles endow co‐crystals diverse stoichiometric ratios and molecular packing modes.

Herein, a box‐type macrocycle (TB[2]) bearing naphthyl‐sidewall Tröger's base skeleton is designed as the electron donor, and an electron‐withdrawing TCNQ is selected as the acceptor. A novel ternary inclusion CT co‐crystal (TB‐TCNQ) with a 1:2 D–A stoichiometric ratio is constructed. Due to the strong host–guest D–A interactions induced by the synergism of multiple π···π and C–H···π/N forces, the co‐crystals exhibit a broad absorption spectrum in the NIR region and exceptional PTC property. Under 808 nm laser irradiation (0.7 W cm^−2^), the temperature of TB‐TCNQ sharply increases to 160 °C within 80 s. A considerably high PTC efficiency of 94.3% is achieved, breaking the record values of all CT co‐crystals reported to date. Considering the excellent PTC performance, porous polyurethane (PU) foam is loaded with the co‐crystals to form an efficient interfacial water evaporation system (TB‐TCNQ@PU) for solar steam generation under simulated solar illumination. A high solar‐evaporation rate of 1.746 kg m^‒2^ h^‒1^ and a significant solar‐to‐vapor efficiency of 93.8% under 1 sunlight irradiation are obtained, demonstrating that the TB‐TCNQ@PU is a highly efficient solar‐thermal conversion material.

## 2 Results and Discussion

### 2.1 Synthesis and Crystal Structure of TB[2]

**Figure 1:**
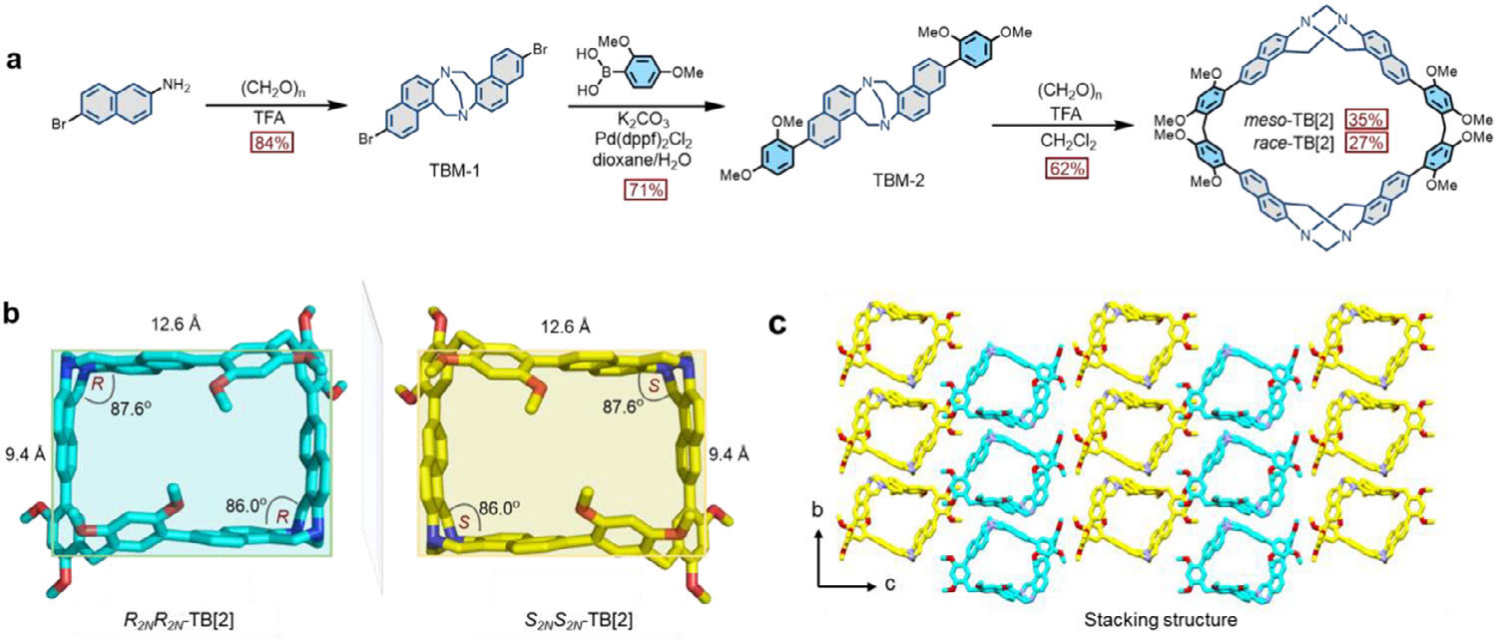
a) Synthetic routes of TB[2] macrocycles. b) The single‐crystal structure of *race*‐TB[2]. c) The stacking structure of *race*‐TB[2]. The hydrogen atoms and solvents are omitted for clarity.

Naphthyl‐sidewall Tröger's base (TB) was chosen as the macrocycle's skeleton due to its π‐electron rich characteristics and rigid clip‐shaped structures. Two 2,4‐dimethoxyphenyl moieties were connected at TB's ends considering that they are good reaction modules for cyclization with aldehydes catalyzed by Lewis acid, as well demonstrated by our previous works.^[^ ^23^ ^]^ In addition, the substituent groups of electron‐rich dimethoxyphenyls and the diazocine nitrogen atoms further increase the electron donating ability of naphthyls. Since the intersection angle of TB is about 90°, dimeric macrocycles with box‐type structures are anticipated to yield. There would be one pair of enantiomers (*race*‐TB[2]) and one *meso*‐isomer (*meso*‐TB[2]) due to TB's inherently *C*_2_‐symmetric chirality. The synthetic procedures of TB[2] are illustrated in **Figure** **1**a. Dibromo‐substituted TB skeleton of TBM‐1 was first prepared by troegeration of 6‐bromonaphthalen‐2‐amine precursor in trifluoroacetic acid (TFA). Then, TBM‐1 reacted with 2,4‐dimethoxybenzeneboronic acid through the Suzuki‐Miyaura coupling reaction to obtain the monomer TBM‐2. Subsequently, the dimeric macrocycles of *meso*‐TB[2] and *race*‐TB[2] were synthesized by a one‐step cyclization of TBM‐2 with paraformaldehyde under the catalysis of TFA with the yields of 35% and 27%, respectively. Although the cyclization has moderate yield, it is considerably satisfactory to the macrocyclic synthesis. The synthetic details are described in the Supporting Information. All chemical structures were confirmed by ^1^H NMR, ^13^C NMR, 2D ^1^H‐^1^H COSY spectroscopy, and high‐resolution mass spectrometry (HRMS) (Figures S1–S15, Supporting Information). It is noted that *meso*‐TB[2] possesses very poor solubility; thus, a small amount of TFA was added to increase its solubility in CDCl_3_ during NMR experiments (Figures S8–S10, Supporting Information).

Single crystals of the monomer TBM‐2 (Figure S16, Supporting Information) and racemic stereoisomers *race*‐TB[2] were successfully obtained, crystallizing in the *P*2_1_/*n* space group (Table S1, Supporting Information) and monoclinic *C*2/*c* space group (Table S2, Supporting Information). As expected, there are one pair of enantiomers (*R*_*2N*_ *R*_*2N*_‐TB[2] and *S*_*2N*_ *S*_*2N*_‐TB[2]), displaying a rigid and box‐shaped geometry with the cavity size of about 12.6 × 9.4 Å (Figure 1b), which is enough to accommodate two small electron‐deficient guests in the cavity. The dihedral angles of the two TB skeletons are 86.0° and 87.6°, respectively, demonstrating the efficient stereoscopic cavity. The packing of the enantiomers in the bc plane forms a 2D tessellations by the C–H···π/N interactions between adjacent TB[2] molecules (Figure 1c; Figures S17–S19, Supporting Information).

### 2.2 Host–Guest Complexation

**Figure 2:**
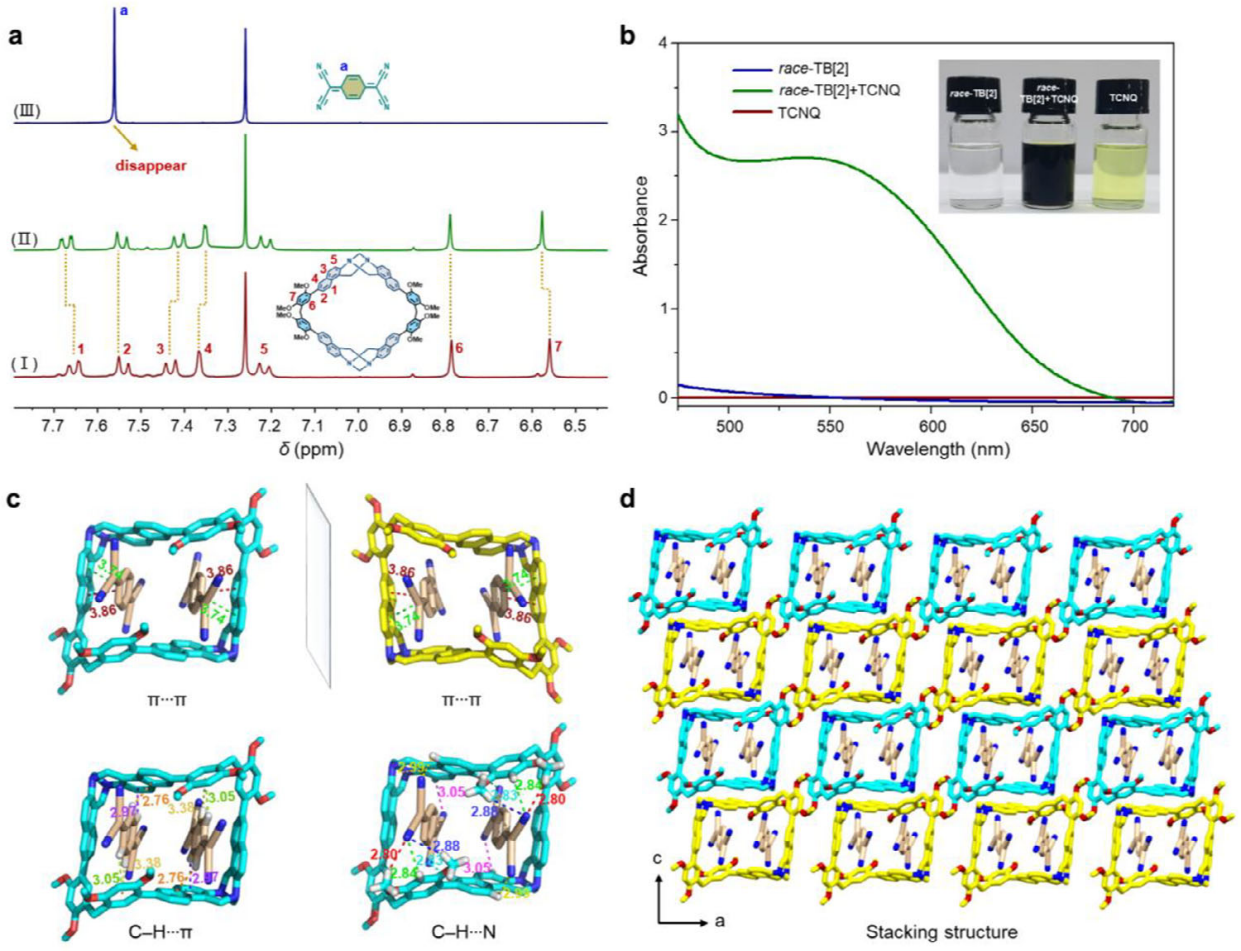
a) Partial ^1^H NMR spectra (400 MHz, 298 K) of (I) free *race*‐TB[2] at 3.0 mm, (II) *race*‐TB[2] (3.1 mm) and 2.0 equiv of TCNQ (5.9 mm), and (III) free TCNQ (6.1 mm) in CDCl_3_. b) UV–vis spectra of *race*‐TB[2], TCNQ and their mixture at 20 mm in CHCl_3_. Inset: the color of *race*‐TB[2], TCNQ and their host–guest mixed solution. c) The π···π, C–H···π, and C–H···N interactions between *race*‐TB[2] and TCNQ in the single‐crystal structure of TB‐TCNQ. d) The stacking mode of TB‐TCNQ. The hydrogen atoms are omitted for clarity.

Due to the electron‐rich and rigid cavity suiting the uptake of planar guest molecules, we chose the typical electron‐deficient TCNQ as guest to investigate the host–guest complexation with *race*‐TB[2] in solution and in the solid state. The mole ratio plot confirmed a 1:2 binding stoichiometry between *race*‐TB[2] and TCNQ in CHCl_3_ (Figure S20, Supporting Information). As shown in **Figure** **2**a, when *race*‐TB[2] (3.0 mm) was added into a CDCl_3_ solution containing 2.0 equiv of TCNQ, the signal related to the protons on TCNQ disappeared compared with the free TCNQ. These results suggest that TCNQ is engulfed inside the macrocycle cavity, thus leading to an efficient shield toward the guest's protons. Simultaneously, the chemical shift of aromatic protons on *race*‐TB[2] moved upfield for protons 3 and 4 and downfield for 1 and 7, which is due to the synergistic results of deshielding effects and π···π interactions between naphthyl and TCNQ. The host–guest complexation of *meso*‐TB[2] toward TCNQ was not investigated due to its poor solubility. The UV–vis spectra displayed a remarkable red‐shift and broad absorption at the range of 475–700 nm of the host–guest mixed solution, which are completely different from the spectrum of individual *race*‐TB[2] or TCNQ, demonstrating the existence of CT interaction between *race*‐TB[2] and TCNQ (Figure 2b). Moreover, an obvious color change to black was observed after mixing the two components in CHCl_3_ (inset pictures in Figure 2b). HRMS also provided an evidence for the encapsulation of two TCNQ inside the *race*‐TB[2] cavity (Figure S21, Supporting Information), wherein the peak related to the 1:2 host–guest complex was found. To determine the host–guest binding affinity, the association constant (*K*_a_) of *race*‐TB[2] with TCNQ was determined by fluorescence titration method in CHCl_3_. A significantly high *K*_a_ value, (1.26 ± 0.04) × 10^5^ m ^‒1^, was determined by a 1:2 host–guest equivalent binding site model (Figure S22, Supporting Information). In sharp contrast, for small‐molecule D–A co‐crystals, the binding investigations are rarely reported in solution because their complexation is too weak to be measured.

X‐ray crystallography further unambiguously confirmed the formation of inclusion complex (Figure 2). The black co‐crystals of TB‐TCNQ with metallic luster were obtained by slow diffusion of Et_2_O into the solution of *race*‐TB[2] and TCNQ in CHCl_3_ (Figures S23, S24, Supporting Information). The crystallographic analysis indicated TB‐TCNQ crystallizes in the monoclinic *C*2/*c* space group with 0.5 *race*‐TB[2] and one TCNQ molecules in the asymmetric unit (Figure S25, Supporting Information), which is consistent with the results in solution. Two TCNQ molecules, orientated in a face‐to‐face manner, are engulfed in the cavity of *race*‐TB[2] forming 1:2 ternary inclusion CT co‐crystals by π···π interactions between naphthyl and TCNQ with the centroid‐to‐centroid distances of 3.74 and 3.86 Å, respectively (Figure 2c). In addition, multiple C–H···π interactions between the hydrogen atoms on TCNQ and aromatic rings of *race*‐TB[2] as well as C–H···N hydrogen bonds between N atoms on TCNQ and hydrogen atoms on *race*‐TB[2] are also beneficial for enhancing the D–A interactions of co‐crystals (Figure 2c). In the stacking structure, the *race*‐TB[2] assembled into 1D infinite channel and TCNQ molecules are isolated and stabilized in the intrinsic cavities (Figure 2d; Figure S26, Supporting Information). In the co‐crystal structure, a large number of molecular‐scale D–A interfaces would benefit the efficient exciton dissociation and exciton transport pathways.

### 2.3 Photophysical Properties of the Co‐Crystals

**Figure 3:**
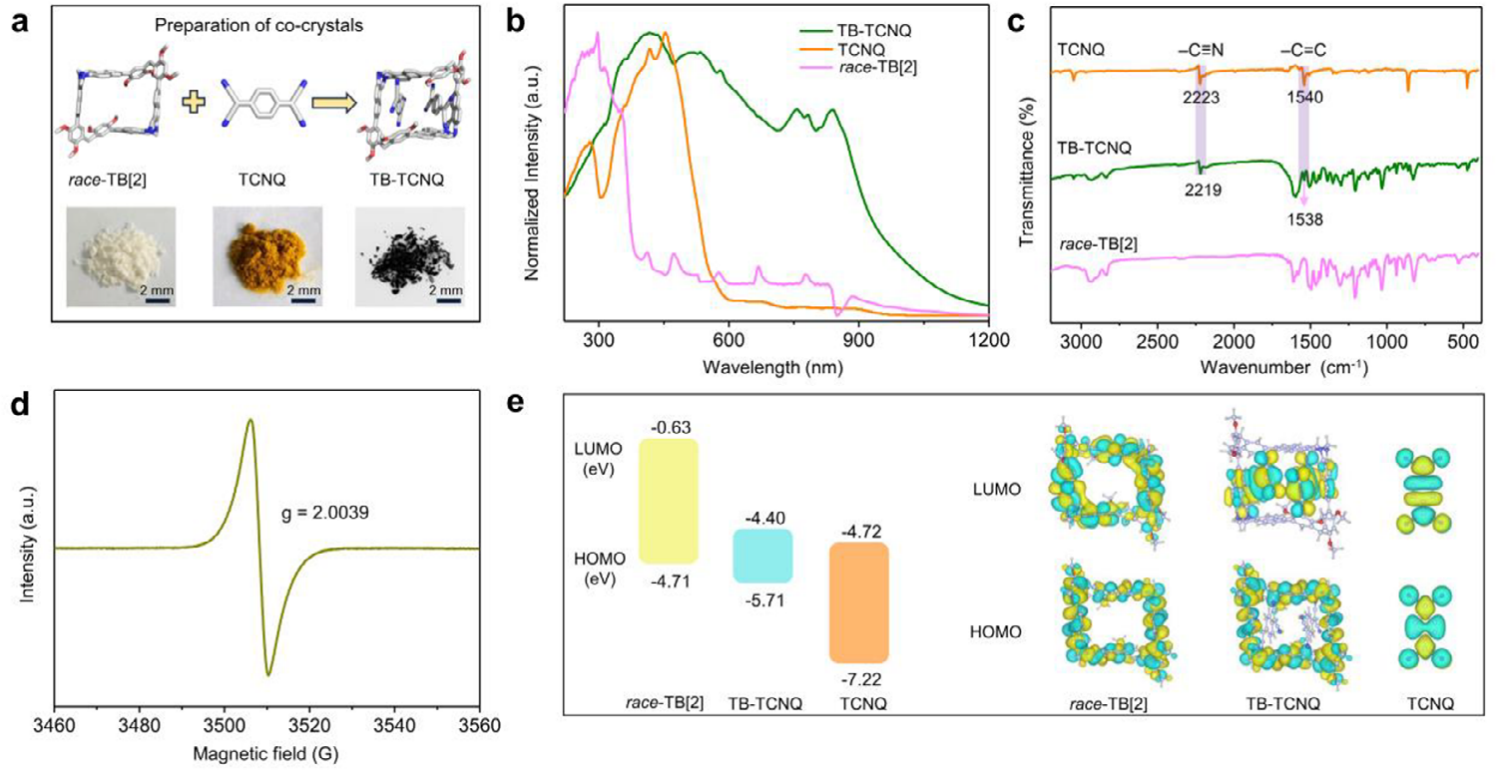
a) Optical images of *race*‐TB[2], TCNQ and TB‐TCNQ. b,c) The UV–vis–NIR absorption and Fourier transform infrared spectra of *race*‐TB[2], TCNQ and TB‐TCNQ. d) EPR spectrum of TB‐TCNQ. e) The calculated energy diagrams and molecular orbital diagrams of *race*‐TB[2], TCNQ and TB‐TCNQ.

The co‐crystals could be prepared on a large scale by facile solution self‐assembly method (**Figure** **3**a). Solid‐state UV–vis–NIR absorption and fluorescence spectra were measured to elucidate the photophysical properties of the co‐crystals. As depicted in Figure 3b, TB‐TCNQ displayed broad absorption band and a large red‐shift absorption peak in the NIR region (700–1200 nm) compared with the individual component of *race*‐TB[2] and TCNQ, which could be attributed to the strong host–guest CT interactions. Moreover, TB‐TCNQ exhibits relatively low reflection and transmission, an adsorption of average 66% in the wavelength range of 200–1200 nm (Figures S27‒S29, Supporting Information), suggesting the good light absorption properties of the co‐crystals. The fluorescence of *race*‐TB[2] was completely quenched upon the co‐crystallization (Figure S30, Supporting Information), indicating TB‐TCNQ can hinder radiative transitions by fluorescence quenching, thus dissipating energy into heat. The Fourier transform infrared (FTIR) and Raman spectra studies reveal that the characteristic peaks of TB‐TCNQ are the combinations of *race*‐TB[2] and TCNQ (Figure 3c; Figure S31, Supporting Information), implying the formation of high‐quality assembly co‐crystals by noncovalent interaction. Besides, the subtle shift changes of vibration characteristic peaks of TCNQ in FTIR and Raman spectra demonstrated the alteration of chemical environments and the augmentation of electron cloud density.^[^ ^24^ ^]^ For instance, the IR characteristic peaks at 2223 cm^‒1^ (C≡N) and 1540 cm^‒1^ (C═C) in TCNQ are red‐shifted to 2219 cm^‒1^ (C≡N) and 1538 cm^‒1^. Electron spin resonance (ESR) showed an intense signal of TB‐TCNQ with the *g* factor of 2.0039 (Figure 3d), which is near to the value of a free electron at around 2.0023,^[^ ^25^ ^]^ indicating the CT characteristic of active radicals and the unpaired electrons. Moreover, thermogravimetric analysis (TGA) gave low weight loss of TB‐TCNQ under 400 °C, supporting its high stability due to the strong intermolecular interactions (Figure S32, Supporting Information).

Density functional theory (DFT) calculations were performed to confirm CT interactions in the inclusion co‐crystals. The geometry structures of the ground state molecules required for the calculations were obtained from the single‐crystal diffraction data. As shown in Figure 3e, the molecular orbital (MO) diagrams presented that the highest occupied molecular orbital (HOMO) of TB‐TCNQ concentrates on the electron‐donor *race*‐TB[2], while the lowest unoccupied molecular orbitals (LUMO) are mainly localized on TCNQ acceptor. The HOMO and LUMO energy levels of TB‐TCNQ were calculated to be −5.71 eV and −4.40 eV, respectively, which are close to the HOMO (−4.71 eV) of *race*‐TB[2] and the LUMO (−4.72 eV) of TCNQ. The co‐crystals exhibited a significantly narrowed bandgap (1.31 eV) compared to the individual *race*‐TB[2] (4.08 eV) and TCNQ (2.50 eV), which is consistent with the experimental absorption spectra. The natural transition orbitals (NTO) analysis was carried out to analyze the electron delocalization and exciton dissociation of CT co‐crystals. As shown in Figure S33 (Supporting Information), the holes were delocalized over TCNQ, and the particles were localized over naphthyl moiety on macrocyclic skeleton, implying CT transition process from *race*‐TB[2] to TCNQ.

### 2.4 Photothermal Conversion Properties

**Figure 4:**
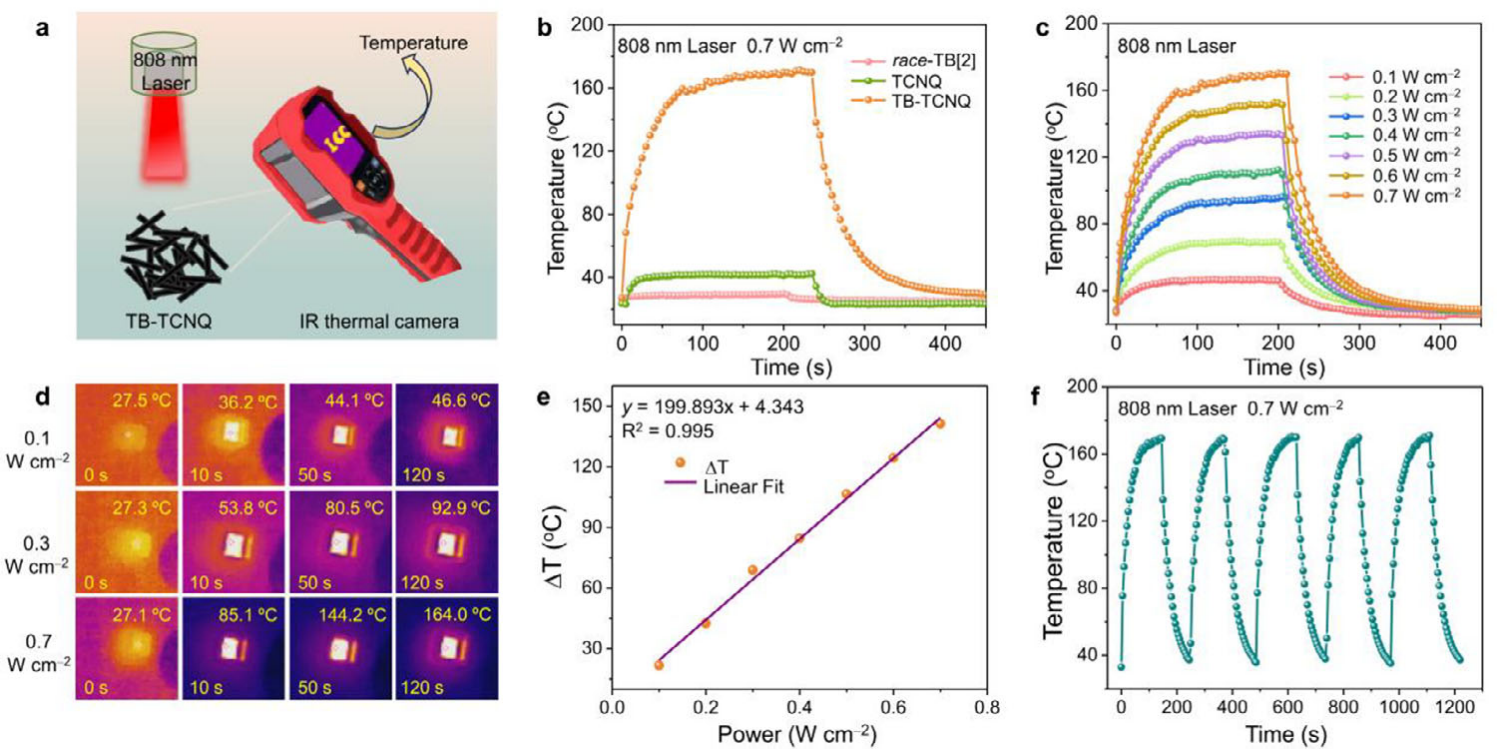
a) The schematic illustration of the PTC measurement. b) PTC behavior of *race*‐TB[2], TCNQ and TB‐TCNQ under 808 nm laser irradiation (0.7 W cm^−2^). c) The heating and cooling curves of TB‐TCNQ under various powers (0.1 to 0.7 W cm^−2^) of 808 nm laser within 450 s. d) Thermal images of TB‐TCNQ under 808 nm laser irradiation at 0.1, 0.3 and 0.7 W cm^−2^. e) The linear relationship between ΔT and laser power for TB‐TCNQ. f) Photothermal cycling test of TB‐TCNQ at a power of 0.7 W cm^−2^.

Considering the intense NIR absorption and good thermal stability, the PTC performance of TB‐TCNQ was investigated under NIR light irradiation recording by an IR thermal camera over time (**Figure** **4**a). Under 808 nm laser irradiation with a power density of 0.7 W cm^‒2^, the temperature of TB‐TCNQ dramatically increased from room temperature to 160 °C within 80 s. By contrast, the temperature of individual component of *race*‐TB[2] had almost no response and TCNQ had an increase of 16 °C under the same condition (Figure 4b). This suggested the TB‐TCNQ has an excellent PTC performance due to the synergistic effect of the host–guest interaction. The maximum temperature‐increasing (∆T) of TB‐TCNQ is positively proportional (y = 199.893x + 4.343) to the laser power density from 0.1 to 0.7 W cm^‒2^ (Figure 4c–e; Figure S34, Supporting Information), revealing the controllability of PTC behavior by altering the excitation power. Moreover, the photothermal heating rate has also linear relationship with power densities within 40 s (Figure S35, Supporting Information). When the laser power density exceeds 0.9 W cm^‒2^, the proportional relationship is no longer maintained (Figure S36, Supporting Information). After five irradiation cycles, the temperature could still maintain a rapid and stable heating rate (Figure 4f). Under continuous laser irradiation (0.7 W cm^‒2^) over 12 h (Figure S37, Supporting Information), the structure of TB‐TCNQ did not change from the powder X‐ray diffraction (PXRD) and Raman spectra (Figures S38, S39, Supporting Information). These results implied that TB‐TCNQ has satisfactorily recyclable and chemical stability. According to the cooling curve under 0.7 W cm^‒2^ (Figure S40, Supporting Information), the heat loss was calculated to be 0.52 W and PTC efficiency of TB‐TCNQ was calculated to be 94.3% by the linear relationship between time and ln(*θ*) (see Supporting Information). To the best of our knowledge, this is the highest value of PTC efficiency of the reported CT co‐crystals so far (Table S4, Supporting Information), which can be comparable with the state‐of‐the‐art PTC materials (Table S5, Supporting Information). We consider the elevated PTC efficiency is attributable to the following reasons. The synergism of C–H···π/N interactions by host–guest encapsulation facilitates the electron transition and delocalization between *race*‐TB[2] and TCNQ, and enhances the D–A interactions. It effectively restrains the emission processes and converts absorbed light into heat via the nonradiative pathways.

### 2.5 Excited‐State Dynamic Investigation

Femtosecond transient absorption (fs‐TA) spectra were employed to clarify the excited‐state dynamics and PTC properties of the host–guest complex. As shown in Figure S41a (Supporting Information), under the 808 nm photoexcitation, two remarkable excited state absorption (ESA) signals at 1035 and 1073 nm appear immediately and the absorption intensity reaches a maximum at 4.0 ps, which could be imputed to the excited CT state. Then, the ESA signals gradually decrease and disappear (Figure S41b, Supporting Information), which is due to the rapid inactivation of the excited CT state through the nonradiative transitions of the internal conversion (IC) process or vibrational relaxation (VR). To investigate the excited‐state kinetics, a two‐exponential fitting was used. The ultrafast decay lifetimes *τ*_1_ and *τ*_2_ were gained by fitting the fs‐TA result at 1035 nm (Figure S41c, Supporting Information). The first decay lifetime *τ*_1_ of 9.15 ± 0.20 ps (amplitude = 91.8%) at 1035 nm can be assigned to the IC transition and superfast VR from the excited state to the ground state. The corresponding second lifetime *τ*_2_ of 185 ± 49.0 ps (amplitude = 8.13%) represents the intersystem crossing (ISC) to the triplet state and then back to the ground state. All of these decay processes contributed to the thermal generation. In a word, when the co‐crystals are excited, the electronic transitions occur from ground CT state to the excited states. The excited electrons subsequently experience nonradiative decay including IC, ISC, VR and charge dissociation and go back to the ground states, which are beneficial for the high‐efficiency PTC.

### 2.6 Solar‐Driven Water Evaporation

**Figure 5:**
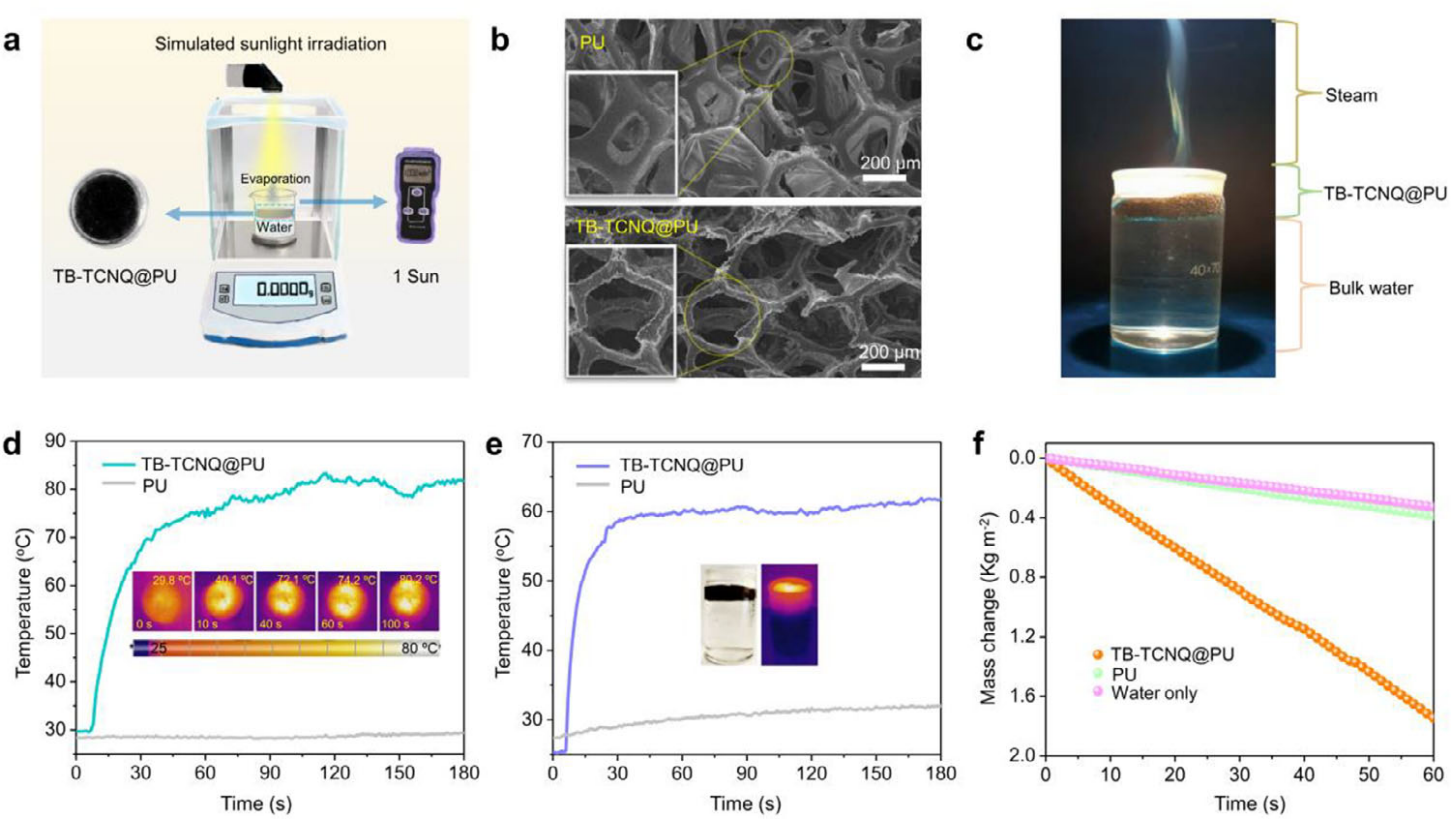
a) The experimental simulation device of solar‐driven water evaporation used to record the water mass change under simulated solar irradiation. b) SEM images of blank PU and TB‐TCNQ@PU. c) The photo of steam generation of TB‐TCNQ@PU under 3 Sun irradiation. d) PTC behavior of TB‐TCNQ@PU and PU under 1 Sun irradiation. Inset shows IR thermal images of TB‐TCNQ@PU under 1 Sun. e) The temperature changes of TB‐TCNQ@PU and PU floating on water under 1 Sun irradiation. Insets represent the optical and IR thermal images of TB‐TCNQ@PU for 30 s. f) Water evaporation curves of only water, PU and TB‐TCNQ@PU under 1 Sun irradiation.

The solar‐driven interfacial water evaporation technology has been considered as a promising and green method for producing freshwater.^[^ ^26^, ^27^ ^]^ It is expected to achieve the low‐cost and low‐pollution supply of freshwater resource. Due to the excellent light‐to‐heat conversion performance and thermal stability of the inclusion co‐crystals, the application of solar‐driven interfacial water evaporation was further investigated under simulated solar illumination. As shown in Figure S42 (Supporting Information), the co‐crystals show a significant temperature response reaching 65 °C under solar irradiation (1 Sun, 1 kW m^‒2^) within 60 s, indicating TB‐TCNQ is a prospective candidate for harvesting solar energy in solar‐driven water evaporation. First, the co‐crystals were introduced into the 3D scaffold of polyurethane (PU) foam to fabricate black TB‐TCNQ‐loaded material (TB‐TCNQ@PU) as an efficient interfacial water evaporation system (**Figure** **5**a; Figure S43, Supporting Information).^[^ ^28^ ^]^ This reason is that the abundant open porous structure of PU can be worked as water channels for replenishment of surface water evaporated, and the low thermal conductivity (0.034 W m^‒1^ K^‒1^) of PU can effectively reduce heat loss. Scanning electron microscope (SEM) of as‐prepared TB‐TCNQ@PU shows that TB‐TCNQ‐loaded foam has a coarse surface and the periodically macroporous structure compared with blank PU foam with smooth surface (Figure 5b; Figure S44, Supporting Information), and UV–vis–NIR gives a higher adsorption of TB‐TCNQ@PU at the range of 300–1500 nm (Figure S45, Supporting Information). The above results manifested the successful load of TB‐TCNQ into PU. Then, the solar PTC of TB‐TCNQ@PU was tested by recording the temperature changes under 1 Sun irradiation. The temperature of the blank sample almost does not enhance under 1 Sun, indicating that PU foam has relatively slight contribution to PTC. In contrast, the equilibrium temperature of TB‐TCNQ@PU foam is 80 °C under the same condition (Figure 5d). Compared with TB‐TCNQ co‐crystals, such high temperature is originated from the two aspects: on one hand, the loose and porous PU foam can realize multiple reflections of incident light, improving the utilization of light; on the other hand, TB‐TCNQ are uniformly distributed in the PU by soak process, effectively reducing the loss of light and heat.^[^ ^29^ ^]^ Meanwhile, the IR thermal images show that after illumination, the surface temperature of the TB‐TCNQ@PU is much higher than that for the blank PU, revealing an excellent solar PTC capability. Next, we also explored the influence of load capacity of TB‐TCNQ into PU on the solar PTC ability by UV–vis–NIR. The absorbance of TB‐TCNQ@PU increases gradually by augmenting the loading amount, but when the loading amount exceeds 4.24 mg cm^‒3^, the absorbance basically remains unchanged with the further addition of TB‐TCNQ (Figure S46, Supporting Information). To further determine the optimal loading of TB‐TCNQ@PU, the PTC of TB‐TCNQ@PU sample with the different loadings was compared under solar irradiation (0.5 kW· m^−2^). It is found that the surface temperature of TB‐TCNQ@PU foam with different loads showed a trend of rising rapidly to equilibrium, and the temperature of TB‐TCNQ@PU basically unchanged when exceeds 4.24 mg cm^‒3^ load (Figure S47, Supporting Information). Therefore, the optimal loading amount of TB‐TCNQ is 4.24 mg cm^‒3^.

The solar‐driven water evaporation of TB‐TCNQ@PU was then studied in a self‐made evaporation system. TB‐TCNQ@PU floating freely on the water surface was used as a heat source for the steam generation system (Figure 5e). Under 1 sun irradiation for 30 s, the surface temperature between TCNQ@PU and water surface, up to 60 °C, is evidently higher than that for PU foam alone. Compared to the standard water temperature, a near 35 °C increment of the surface water temperature greatly promotes the water evaporation speed. Upon raising the illumination intensity from 1 to 3 Sun, rapid temperature rise and steam generation can be definitely observed at the top of the glass (Figure 5c; Movie S1 and Figure S48, Supporting Information). To estimate the efficiency of solar‐driven water evaporation, the water mass reduction was measured in real‐time during irradiation process. As illustrated in Figure 5f, the evaporation of water with TB‐TCNQ@PU foam was significantly accelerated compared to pure PU foam. The solar energy‐to‐vapor efficiency (*η*) and water evaporation rate were evaluated up to 93.8% and 1.746 kg m^‒2^ h^‒1^ for TB‐TCNQ@PU under 1 Sun (see Supporting Information), respectively, which reaches the high level of composite materials for solar‐driven water evaporation (Table S6, Supporting Information).

### 2.7 Application of TB‐TCNQ@PU in Pure Water Production

**Figure 6:**
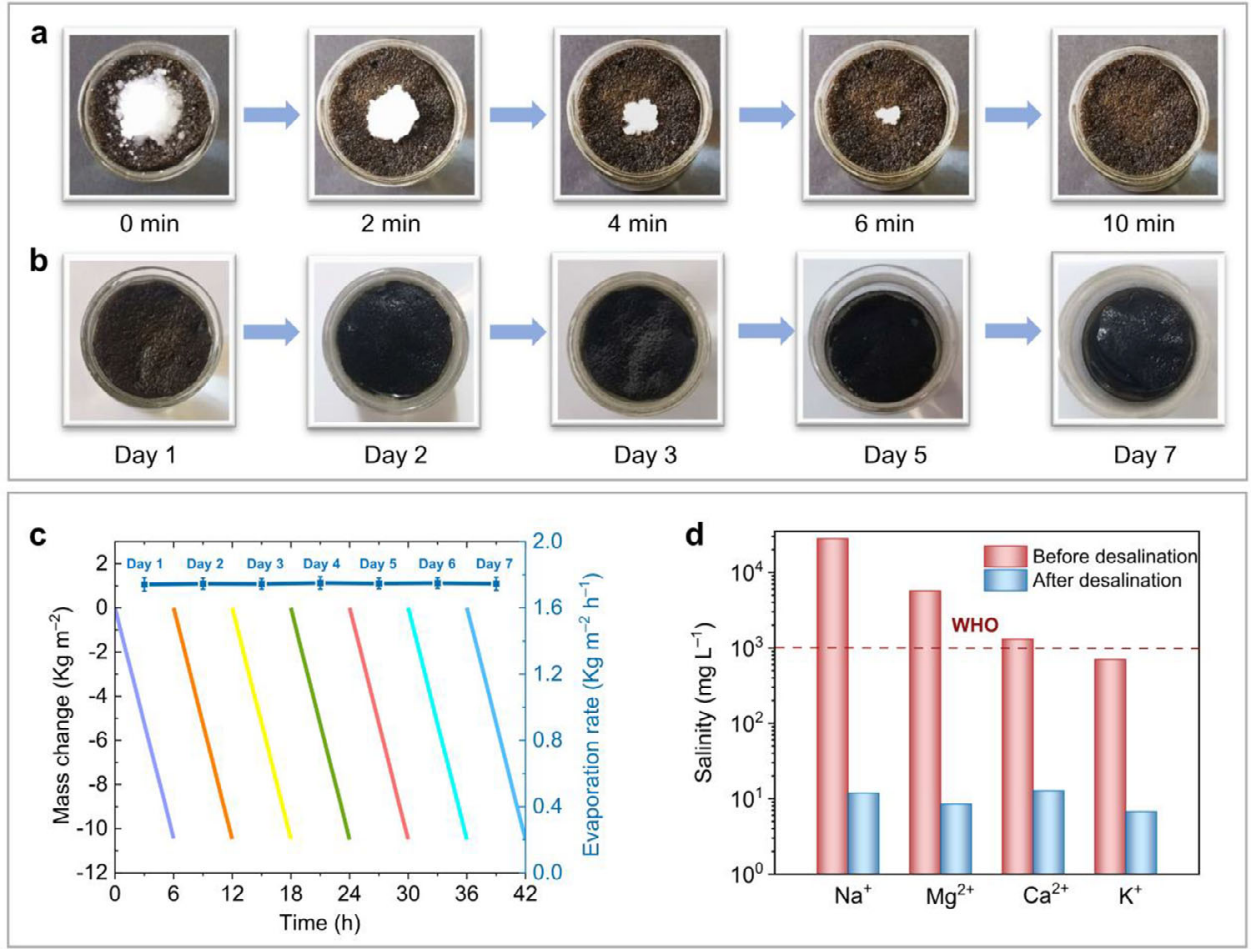
a) Optical picture of NaCl dissolution on TB‐TCNQ@PU within 10 min under solar irradiation. b) Optical picture of TB‐TCNQ@PU in the 7‐day cyclic experiment, indicating no salt accumulation on the material surface. c) The recycle and durability performance of TB‐TCNQ@PU under 1 sun irradiation for 7 days. d) The measured concentrations of four primary ions of simulated seawater before and after desalination.

The salt resistance and durability of materials as important parameters for solar‐driven interfacial evaporation were also investigated. First, the resistance of TB‐TCNQ@PU was tested by directly placing 3 g NaCl salt granules on the upper surface of evaporator. As shown in **Figure** **6**a, the salt of NaCl gradually dissolve and completely disappear within 10 min under solar irradiation, indicating the excellent salt resistance and efficient desalting rate of TB‐TCNQ@PU evaporator. The ordered porous microchannels are conducive to rapid water transport. Salt ions undergo anti‐concentration diffusion and convection during solar water evaporation process, thus a constant flow of vapor dissolves the solid NaCl, allowing it to migrate rapidly from the upper photo‐evaporation layer to the lower bulk water layer and not to accumulate in the photothermal layer.^[^ ^30^ ^]^ The long‐term stability of TB‐TCNQ@PU was then evaluated through cyclic tests involving daily 6 h sun illumination for seven consecutive days, with dark periods simulating natural day‐night cycles. The material demonstrated consistent solar desalination performance in brine, maintaining linear mass changes throughout the testing period. A stable evaporation rate of ≈1.7 kg m⁻^2^ h⁻¹ persisted across all cycles (Figure 6c), confirming excellent operational durability for sustained solar‐driven water evaporation applications. No salt deposition was observed on the surface of TB‐TCNQ@PU (Figure 6b).

To further evaluate the practical applications of TB‐TCNQ@PU composite for solar‐driven desalination, we conducted the experiments using simulated seawater containing four primary ions of Na^+^, Mg^2+^, K^+^, Ca^2+^ in a controlled evaporation system. The purification efficiency was quantitatively analyzed through inductively coupled plasma mass spectrometry (ICP‐MS) measurements comparing ionic concentrations before and after desalination. As illustrated in Figure 6d, the results showed remarkable reduction of the ion concentrations by ≈ 3 or 4 orders of magnitude after purification, which meets the potable water standard of the World Health Organization (WHO), indicating good performance for potential seawater desalination.

## 3 Conclusion

In summary, we demonstrate that inclusion co‐crystal engineering, promoting D−A binding through host–guest encapsulation, is an efficient strategy to boost PTC performance. We designed and synthesized an electron‐rich box‐shaped TB[2] by three‐step facile and efficient reactions, which could encapsulate two electron‐deficient TCNQ molecules into its cavity in the solid state through the synergistic effect of π···π interactions and C–H···π/N hydrogen bonds. The inclusion co‐crystals of TB‐TCNQ could be prepared on a large scale by facile solution method. Under 808 nm laser illumination, TB‐TCNQ displayed extremely high PTC efficiency of 94.3%. The cooperation of multiple noncovalent interactions by inclusion complexation favors the electron transition and delocalization, and enhances D–A interactions, thus leading to excellent PTC performance through the promoted nonradiative transition. Furthermore, due to the high PTC efficiency and a good photostability, a solar‐driven water evaporation system was constructed by introducing the co‐crystals into porous PU foam to generate the heating source of TB‐TCNQ@PU. The water evaporation rate and efficiency of TB‐TCNQ@PU foam are calculated to be 1.746 kg m^‒2^ h^‒1^ and 93.8%, respectively. This work provides a novel and promising strategy to build highly efficient PTC materials by the combination of host–guest chemistry and organic co‐crystal engineering.

## 4 Experimental Section

### Materials and Apparatus

6‐Bromonaphthalen‐2‐amine (98%), 2,4‐dimethoxyphenylboronic acid (98%), and TCNQ (99%) were purchased from the commercial source without further purification. The polyurethane (PU) foam was purchased from commercial source. Nuclear magnetic resonance (NMR) spectra were recorded on Bruker Avance III 400 or 600 MHz. Chemical shifts are reported in ppm relative to the signals corresponding to the residual non‐deuterated solvents (CDCl_3_: *δ*_H_ = 7.26 ppm and *δ*_C_ = 77.0 ppm). High‐resolution mass spectra (HRMS) were determined on Agilent Accurate‐Mass Q‐TOF 6520 instrument with ESI source and Bruker solariX XR with MALDI source. All single crystal X‐ray diffraction data were collected on a Bruker D8 Venture or Bruker APEX‐II CCD diffractometer using Cu‐*K*α radiation (*λ* = 1.54178 Å). Using Olex2,^[^ ^31^ ^]^ the structures were solved with the SHELXT^[^ ^32^ ^]^ structure solution program using Intrinsic Phasing and refined with the SHELXL refinement package using Least Squares minimization. UV–vis absorption spectra were recorded on an Agilent Cary 100 UV–vis. The UV–vis–NIR absorption spectra were performed using a Shimadzu UV3600 plus spectrometer. Fluorescence spectra were performed with an Agilent Cary Eclipse spectrophotometer. Optical microscopy images were collected on a SOPTOP ICX41 imaging system. Powder X‐ray diffraction (PXRD) patterns were obtained from a Rigaku Smartlab SE/ X‐ray powder diffractometer at 40 kV and 40 mA for Cu *K*α radiation (*λ* = 1.5418 Å), with a scan speed of 6° min^−1^ and a step size of 0.02^°^ in 2*θ*. Scanning electron microscopy (SEM) with a TESCAN MIRA LMS instrument at an accelerating voltage of 5–15 keV. The ESR spectrum was measured on a Bruker EMX PLUS instrument. The Raman spectra were measured on a Horiba LabRAM HR Evolution with 532 nm laser excitation. The Fs‐TA spectra were measured with a sapphire laser system (800 nm, 35 fs, 6 mJ pulse^−1^, and 1 kHz repetition rate), and TOPAS Optical Parametric Amplifier (OPA).

### Theoretical Calculation

First‐principles calculations for structure optimizations and electronic self‐consistent field (SCF) calculations were performed using density functional theory (DFT) by implementing ORCA quantum chemistry program (version 5.0.4).^[^ ^33^ ^]^ The B3LYP hybrid functional was employed for electronic SCF energies and expand molecular wave functions by the Pople 6–31G* basis set. The Becke–Johnson damping version (D3BJ) atom‐pairwise dispersion corrections were applied to account for weak intermolecular interactions. The visualization of the HOMO/LUMO molecular orbitals were carried out by the VESTA software with orbital data extracted by the Multiwfn program.^[^ ^34^ ^]^ The excited state property of TB‐TCNQ was calculated at CAM‐B3LYP/6–311G* level. Natural transition orbitals (NTOs) were evaluated with the dominant particle‐hole pair contributions and the associated transition weights. The orbitals were visualized by VMD program (the isovalue was set as 0.02) assisted with the Multiwfn program.

### Photothermal Conversion Property Measurement

Photothermal tests were carried out under 808 nm laser irradiation (DL‐S‐808‐3000 mW, China). Real‐time temperature changes were recorded using an infrared thermal imager (HM‐TPK20‐3AQF/W, China). The photothermal effect was measured by monitoring the temperature of the TB‐TCNQ co‐crystal powders. To evaluate the single wavelength photothermal conversion efficiency, the co‐crystal powders were irradiated under the NIR laser (0.7 W cm^−2^) for 235 s. After switching off the laser, the temperature was further measured for another 215 s.

### Preparation of TB‐TCNQ@PU Foams

The co‐crystals with different masses were first dissolved into dichloromethane solution. Then, the porous 3D polyurethane (PU) foam with 3 cm diameter and 1 cm height was repeatedly impregnated the solution containing TB‐TCNQ until the TB‐TCNQ was completely soaked up. The PU foam loading TB‐TCNQ was finally dried at 80 °C for 2 h to obtain black TB‐TCNQ@PU foam, namely a heat source for interfacial water evaporation system.

### Solar‐Driven Water Evaporation

Simulated solar illumination was provided by a 300 W xenon lamp equipped with an AM 1.5 G filter (CEL‐PF300‐T6, Beijing China Education Au‐light Co. Ltd., China) to replicate a full solar spectrum (200‒2500 nm). The experiments were typically conducted at an ambient of 25 °C. A beaker (40 mm inner diameter, and 70 mm height) is first filled with water. A PU foam or TB‐TCNQ@PU foam with sizes of (30 mm inner diameter and 10 mm height) was freely floated on the water. The simulated sunlight (1 kW m^‒2^) device was placed on the top to illuminate the foam. Temperature changes were real‐time monitored with an IR camera. Mass of water evaporated was measured with a high accuracy balance (0.001 mg inaccuracy) for the evaluation of the evaporation rate and solar‐thermal conversion efficiency.^[^ ^35^ ^]^

### Seawater Desalination

The desalination system employed laboratory‐prepared seawater samples. Ionic concentrations of four principal seawater constituents (Na⁺, Mg^2^⁺, Ca^2^⁺, K⁺) were quantified using inductively coupled plasma mass spectrometry (ICP‐MS, NexION 350D) before and after desalination.

### Crystallographic Data

CCDC 2 394 473 (TBM‐2), 2 385 605 (*race*‐TB[2]), and 2 385 606 (TB‐TCNQ) contains the supplementary crystallographic data for this paper. These data can be obtained free of charge from The Cambridge Crystallographic Data Centre via www.ccdc.cam.ac.uk/data_request/cif.

## Conflict of Interest

The authors declare no conflict of interest.

## Author Contributions

R.W. and Y.S. contributed equally to this work. B.L. and C.L. conceived the project. R.W. performed most of the experiments including the synthesis, characterization and the photophysical properties. Y.S. contributed the photothermal conversion measurement. Z.G. and K.L. contributed theoretical calculations. R.W., B.L. and C.L. finished the preparation of manuscript. All authors discussed and commented on the paper.

## Supporting information



Supporting Information

Supplemental Movie 1

## Data Availability

The data that support the findings of this study are available in the supplementary material of this article.
