# Supplementary material for: Ternary Inclusion Co‐Crystals for Efficient Photothermal Conversion and Solar‐Driven Water Evaporation
Source: Adv Sci (Weinh). 2025 Apr 29;12(23):2500050. doi: 10.1002/advs.202500050 (PMC12199327; doi:10.1002/advs.202500050)
Supplement: Supplementary file 1 — Supporting Information [file ADVS-12-2500050-s002.pdf]

ADVANCED SCIENCE
Open Access

Supporting Information

for *Adv. Sci.*, DOI 10.1002/advs.202500050

Ternary Inclusion Co-Crystals for Efficient Photothermal Conversion and Solar-Driven Water Evaporation

Ruotong Wang, Yi Su, Zhiyu Xiao, Tongtong Wang, Kun Liu, Zhihao Gong, Jiabin Wu, Junyi Chen, Zhixue Liu, Jingjing Li, Yu-Hui Zhang, Lu Wang, Bin Li, Xiaotao Zhang* and Chunju Li**

Supplementary Information for

Ternary Inclusion Co-crystals for Efficient Photothermal Conversion and Solar-Driven Water Evaporation

Ruotong Wang,[#a] *Yi Su,*[#b] *Zhiyu Xiao,*[a] *Tongtong Wang,*[a] *Kun Liu,*[a] *Zhihao Gong,*[a] *Jiabin Wu,*[a] *Junyi Chen,*[a] *Zhixue Liu,*[a] *Yu-Hui Zhang,*[c] *Lu Wang,*[a] *Bin Li,**[a] *Xiaotao Zhang**[b] *and Chunju Li**[a]

[a]*Academy of Interdisciplinary Studies on Intelligent Molecules, Tianjin Key Laboratory of Structure and Performance for Functional Molecules, College of Chemistry, Tianjin Normal University, Tianjin 300387, PR China*
[b]*Key Laboratory of Organic Integrated Circuit, Ministry of Education & Tianjin Key Laboratory of Molecular Optoelectronic Sciences, Department of Chemistry, Institute of Molecular Aggregation Science, Tianjin University, Tianjin 300072, China*
[c]*College of Science & College of Material Science and Art Design, Inner Mongolia Agricultural University, Hohhot 010018, China*
Corresponding Author
**E-mail: bli@tjnu.edu.cn; zhangxt@tju.edu.cn; cjli@shu.edu.cn*

Table of Contents

1. **Synthesis and single-crystal structures of the monomer and *race*-TB[2]** **2**
2. **Host-guest complexation studies** **15**
3. **Photophysical properties of TB-TCNQ** **19**
4. **Photothermal conversion studies** **22**
5. **Solar-driven water evaporation** **28**
6. **The optimized structure coordinates** **33**
7. **References** **42**

1. Synthesis and single-crystal structures of the monomer and *race*-TB[2]

1.1 Synthesis of TBM-1

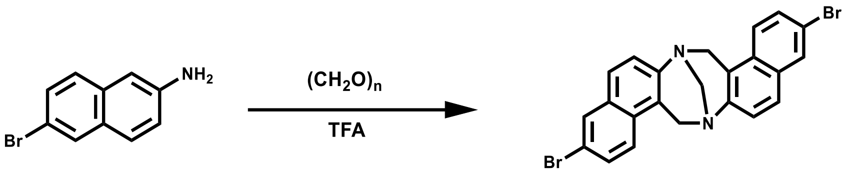

To a 500 mL flask was added 6-Bromonaphthalen-2-amine (5.11 g, 23.0 mmol), paraformaldehyde (1.73 g, 57.5 mmol) and trifluoroacetic acid (50 mL). The mixture was stirred at room temperature for 24 h. After quenching by saturated $NaHCO_3$ (aq., 30.0 mL), the water phase was extracted with CH_2Cl_2 (3 × 30 mL) and the organic phase was washed with saturated NaCl (aq.), respectively. The organic phase was then dried over anhydrous Na_2SO_4 and subsequently concentrated under reduced pressure. The residue was purified by column chromatography on silica gel (eluent petroleum ether: ethyl acetate 6 : 1, *v*/*v*) to afford **TBM-1** as a brown solid (9.23 g, 84.0 %). ^{1}H NMR (400 MHz, $CDCl_3$) δ 7.87 (s, 2H), 7.57 (d, J = 8.8 Hz, 2H), 7.53 (s, 4H), 7.36 (d, J = 8.8 Hz, 2H), 5.01 (d, J = 16.7 Hz, 2H), 4.69 (d, J = 16.8 Hz, 2H), 4.51 (s, 2H). ^{13}C NMR (101 MHz, $CDCl_3$) δ 145.7, 132.1, 130.6, 129.9, 129.8, 127.1, 125.8, 123.0, 121.4, 118.6, 66.7, 55.5. HRMS (ESI) m/z: [TBM-1 + H]$^+$ Calcd for $C_{23}H_{17}Br_2N_2^+$ 480.9733; found 480.9732.

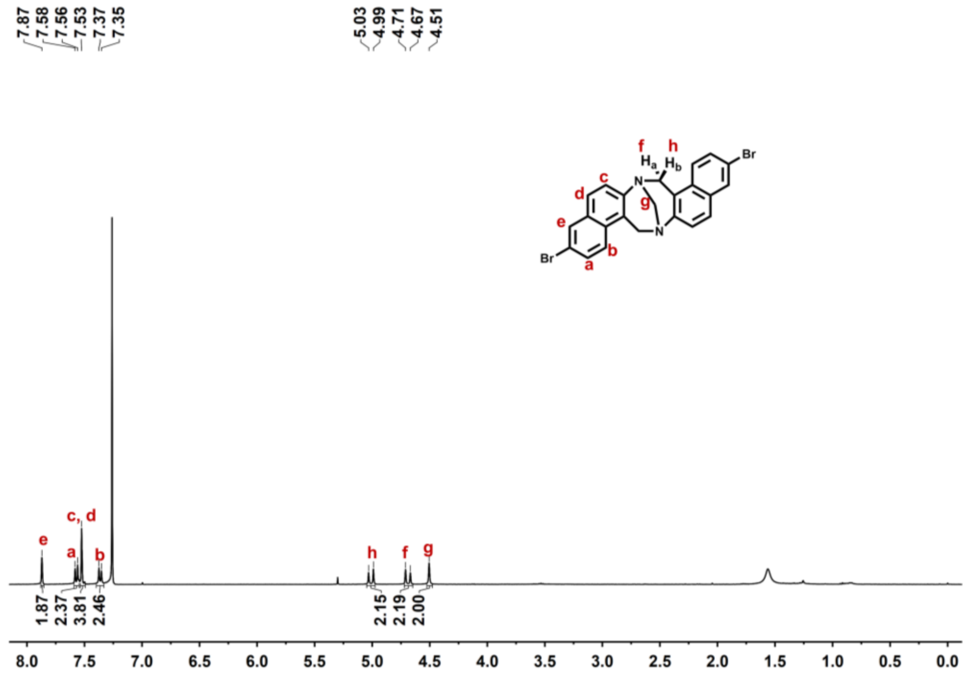

Figure S1. ^{1}H NMR spectrum (400 MHz, $CDCl_3$, 298K) of **TBM-1**.

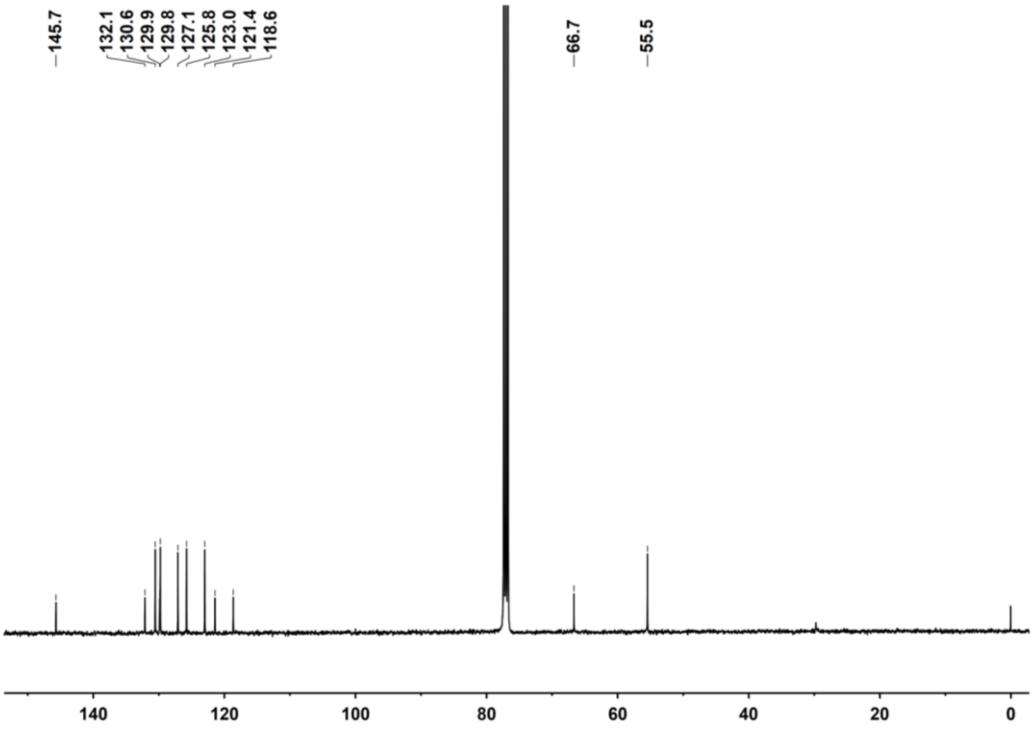

Figure S2. ^{13}C NMR spectrum (101 MHz, $CDCl_3$, 298K) of **TBM-1**.

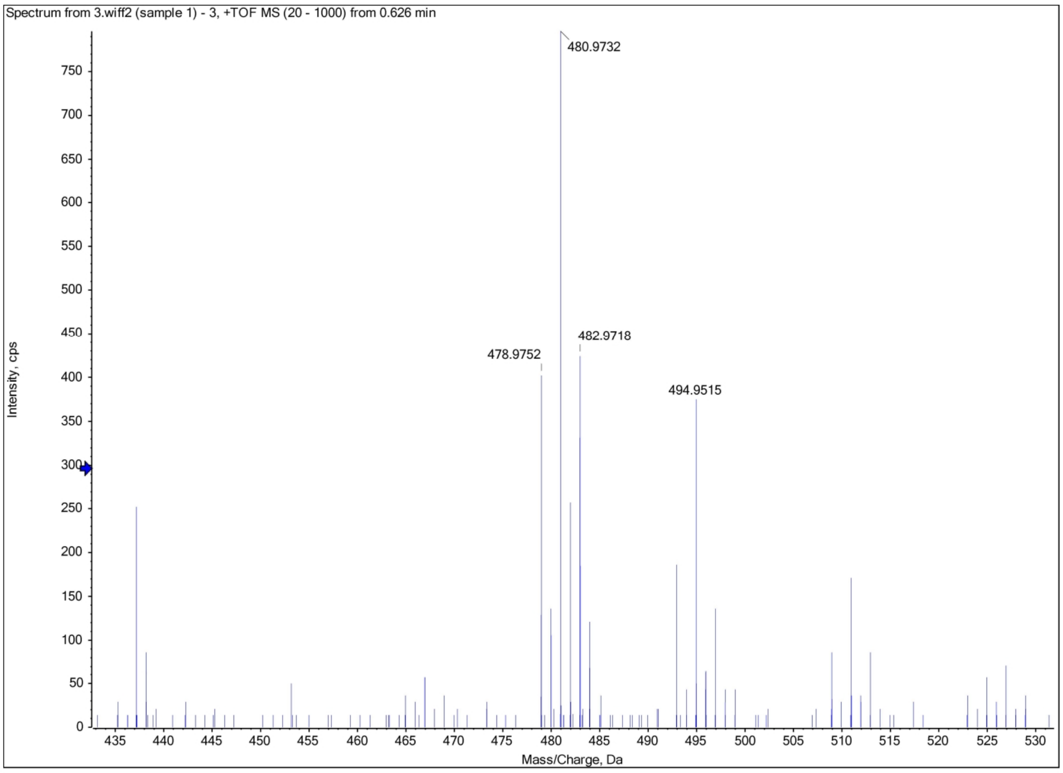

Figure S3. HRMS of **TBM-1**.

1.2 Synthesis of TBM-2

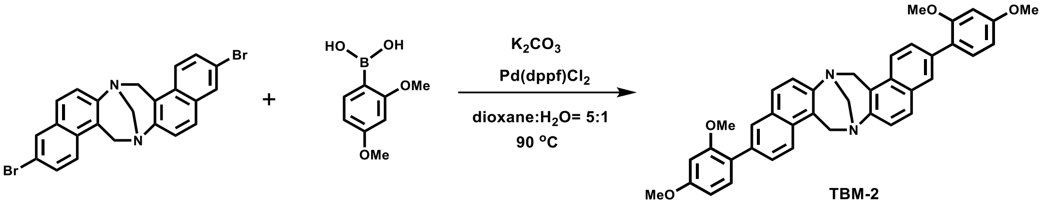

To a 500 mL flask was added TBM-1 (4.80 g, 10.0 mmol), 2,4-dimethoxyphenylboronic acid (4.55 g, 25.0 mmol), [1,1'-Bis(diphenylphosphino)ferrocene]dichloropalladium (0.37 g, 0.50 mmol) and K_2CO_3 (5.53 g, 40.0 mmol) in 1,4-dioxane (150 mL) and water (30 mL). The mixture was stirred and refluxed under nitrogen atmosphere for 12 hours. After cooling down to room temperature, most of the solvents was removed under reduced pressure, then extracted with CH_2Cl_2 (3 × 30mL). The organic layer was dried over anhydrous Na_2SO_4 and subsequently concentrated under reduced pressure. The residue was purified by column chromatography on silica gel (eluent petroleum ether : ethyl acetate 5 : 1, *v*/*v*) to afford **TBM-2** as a white solid (4.21g, 71.0 %). ^{1}H NMR (400 MHz, $CDCl_3$) δ 7.78 (d, J = 1.4 Hz, 2H), 7.69 (s, 1H), 7.67 (d, J = 2.4 Hz, 2H), 7.66 (s, 1H), 7.64 (d, J = 1.7 Hz, 1H), 7.62 (d, J = 1.7 Hz, 1H), 7.35 (d, J = 8.8 Hz, 2H), 7.28 (d, J = 1.5 Hz, 2H), 6.58 (d, J = 2.4 Hz, 1H), 6.56 (s, 3H), 5.06 (d, J = 16.6 Hz, 2H), 4.80 (d, J = 16.6 Hz, 2H), 4.57 (s, 2H), 3.85 (s, 6H), 3.76 (s, 6H). ^{13}C NMR (101 MHz, $CDCl_3$) δ 160.4, 157.7, 145.0, 135.0, 131.4, 130.9, 130.0, 128.7, 128.6, 128.0, 124.6, 123.4, 121.1, 120.6, 104.7, 99.1, 67.0, 55.8, 55.6, 55.5, 29.8. HRMS (ESI) m/z: [TBM-2 + H]$^+$ Calcd for $C_{39}H_{35}N_2O_4^+$ 595.2591; found 595.2591.

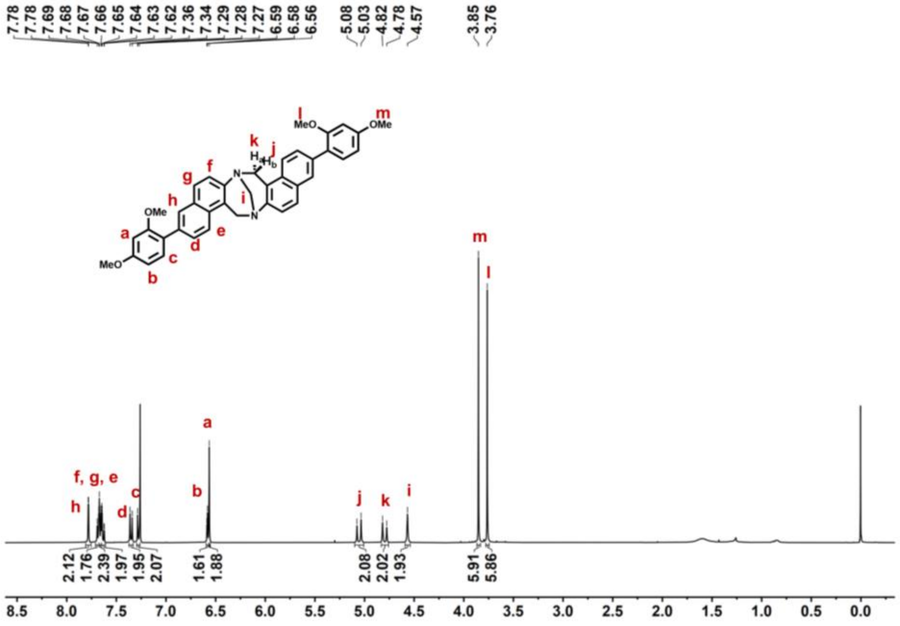

Figure S4. ^{1}H NMR spectrum (400 MHz, $CDCl_3$, 298K) of **TBM-2**.

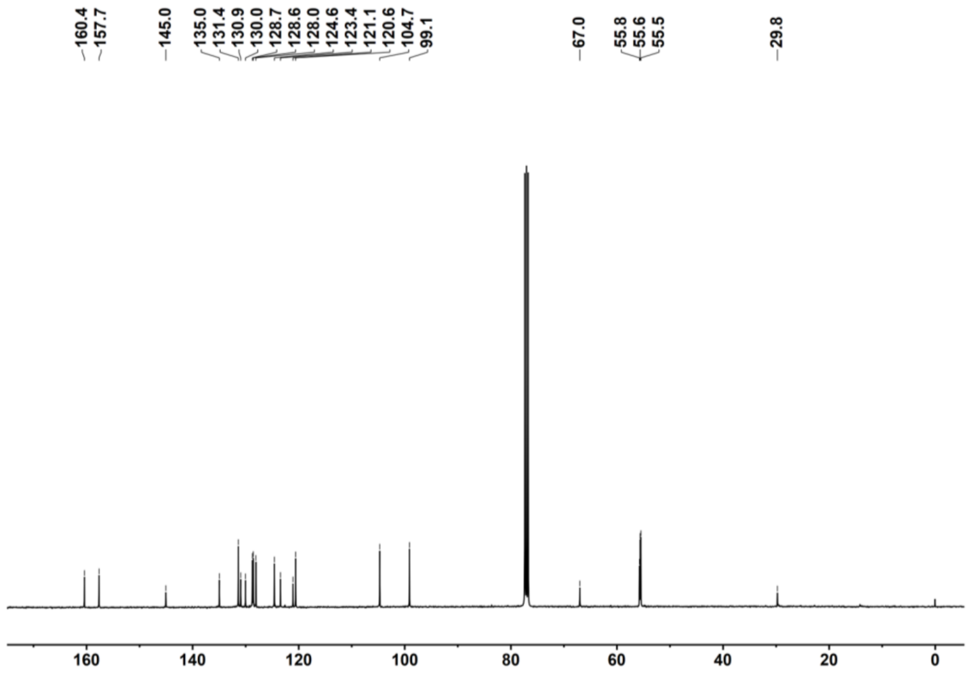

Figure S5. ^{13}C NMR spectrum (101 MHz, $CDCl_3$, 298K) of **TBM-2**.

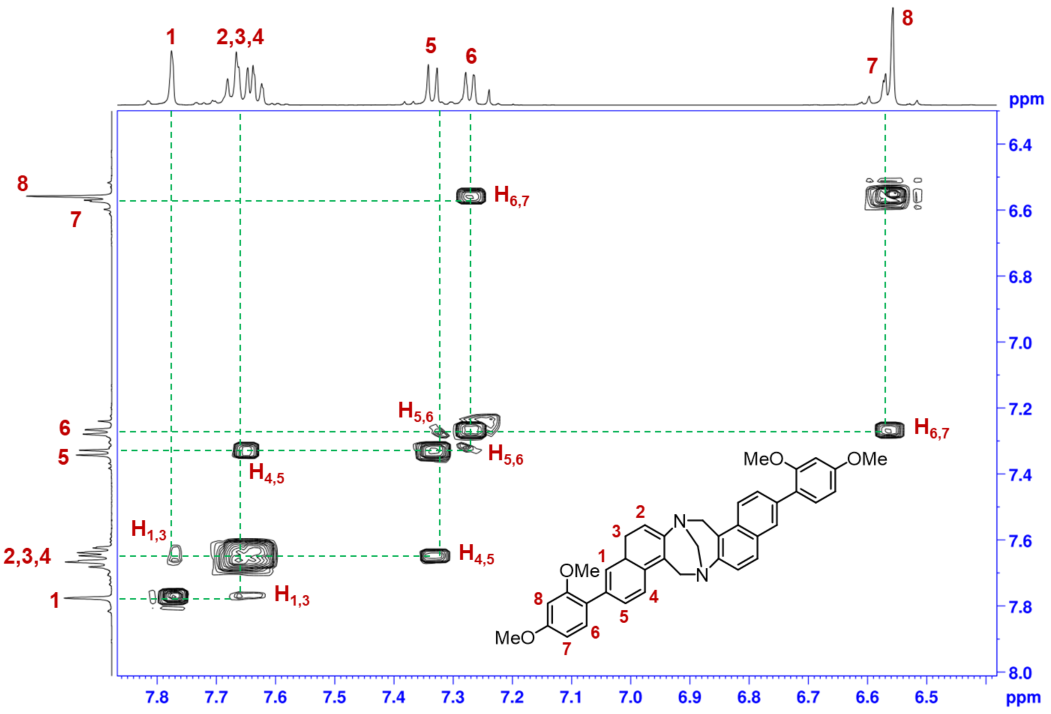

Figure S6. 2D ^{1}H-^{1}H COSY spectrum (600 MHz, 298 K) of **TBM-2** in $CDCl_3$.

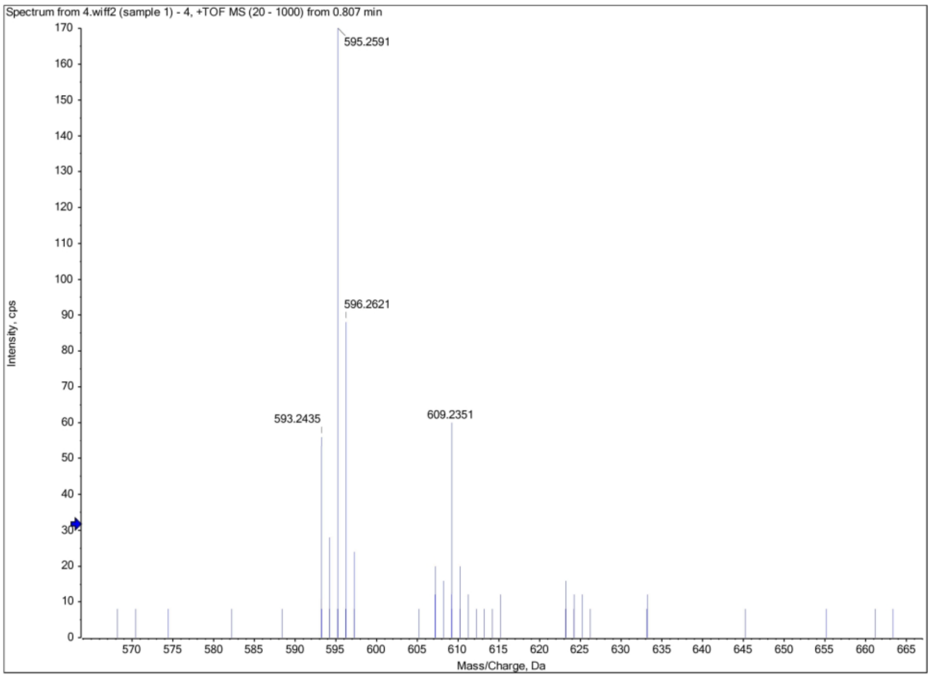

Figure S7. HRMS of **TBM-2**.

1.3 Synthesis of TB[2]

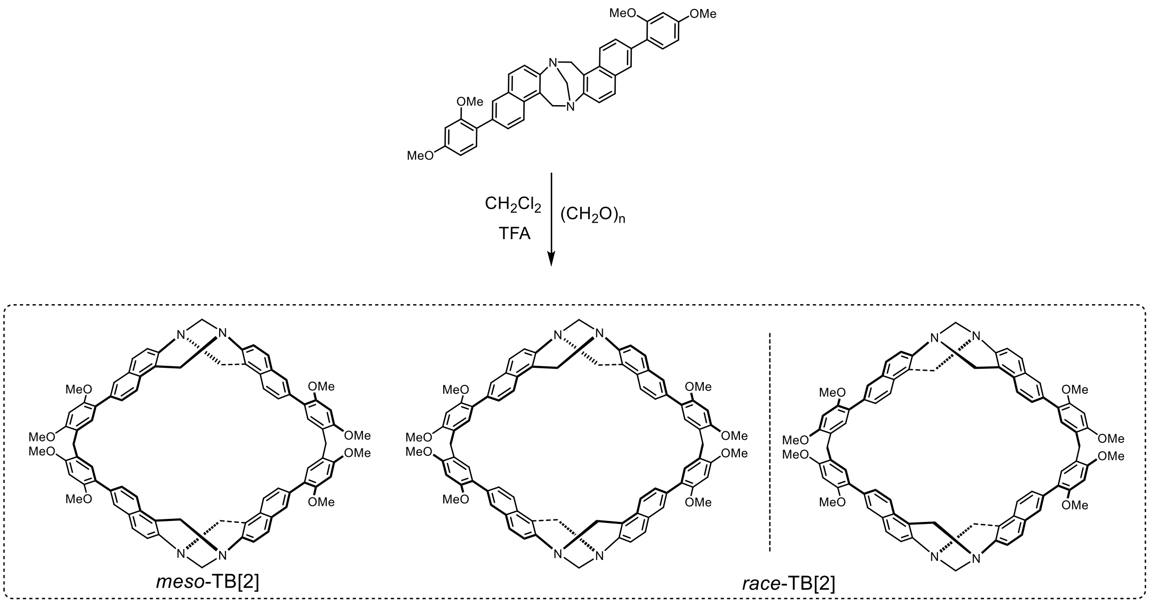

To the solution of **TBM-2** (100 mg, 0.17 mmol) and paraformaldehyde (12.8 mg, 0.43 mmol) in dichloromethane (20 mL) was added TFA (1.50 mL, 0.02 mol) and stirred at room temperature for 30 minutes. After quenching by saturated $NaHCO_3$ (aq. 30 mL), the water phase was extracted with CH_2Cl_2 (3 × 30 mL) and the organic phase was washed with saturated NaCl (aq.), respectively. The organic phase was then dried over anhydrous Na_2SO_4 and subsequently concentrated under reduced pressure. The residue was purified by column chromatography on silica gel

(CH_2Cl_2 : CH_3OH = 120 : 1, *v*/*v*) to afford ***meso*-TB[2]** (71.7 mg, 35%) and ***race*-TB[2]** (55.3 mg, 27%) as a white solid.

***meso*-TB[2]**: ^{1}H NMR (400 MHz, $CDCl_3$) δ 7.79 (s, 4H), 7.73 (d, J = 8.9 Hz, 4H), 7.59 (s, 8H), 7.45 (d, J = 8.9 Hz, 4H), 6.85 (s, 4H), 6.65 (s, 4H), 5.33 (d, J = 16.6 Hz, 4H), 5.17 (s, 4H), 4.96 (d, J = 16.7 Hz, 4H), 4.09 (d, J = 16.7 Hz, 2H), 3.93 (s, 12H), 3.80 (s, 12H), 3.71 (d, J = 17.2 Hz, 2H). ^{13}C NMR (101 MHz, $CDCl_3$) δ 158.0, 155.6, 137.5, 135.7, 132.5, 132.0， 131.8, 130.8, 130.4, 128.7, 128.3, 122.3, 122.3， 122.1, 121.1, 120.7, 118.5, 97.7, 67.4, 55.2. HRMS (ESI) m/z: *meso*-TB[2] Calcd for $C_{80}H_{68}N_4O_8$ 1212.5037; found 1212.5041.

***race*-TB[2]**: ^{1}H NMR (400 MHz, $CDCl_3$) δ 7.66 (d, J = 8.7 Hz, 4H), 7.54 (d, J = 8.7 Hz, 4H), 7.43 (d, J = 8.8 Hz, 4H), 7.37 (s, 4H), 7.22 (d, J = 8.7 Hz, 4H), 6.79 (s, 4H), 6.56 (s, 4H), 4.97 (d, J = 16.7 Hz, 4H), 4.68 (d, J = 16.6 Hz, 4H), 4.55 (s,4H), 3.92 (s, 12H), 3.88 (s, 3H), 3.78 (s, 1H), 3.71 (s, 12H). ^{13}C NMR (101 MHz, $CDCl_3$) δ 158.0, 155.9, 144.6, 135.5, 132.0, 131.0, 129.8, 129.0, 128.2, 127.9, 124.1, 122.6, 121.0, 120.9, 120.0, 95.9, 67.1, 56.1, 55.9, 55.8. HRMS (ESI) m/z: *race*-TB[2] Calcd for $C_{80}H_{68}N_4O_8$ 1212.5037; found 1212.5052.

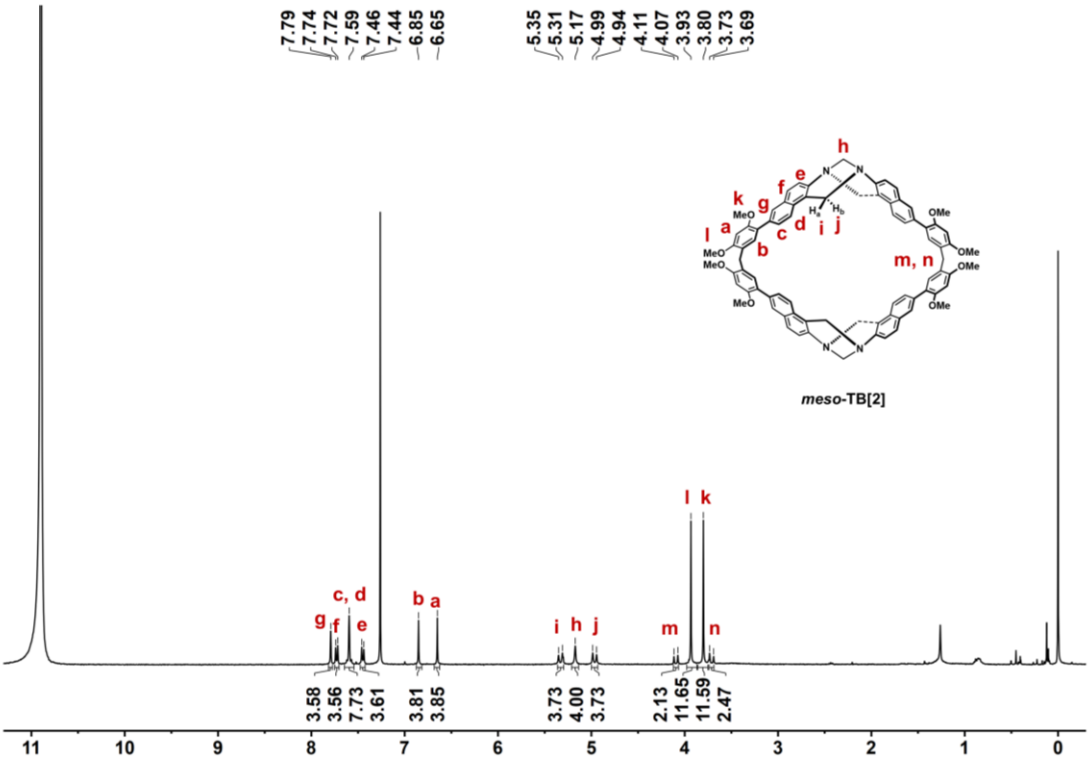

Figure S8. ^{1}H NMR spectrum (400 MHz, 298K) of ***meso*-TB[2]** in $CDCl_3$ with a small amount of TFA.

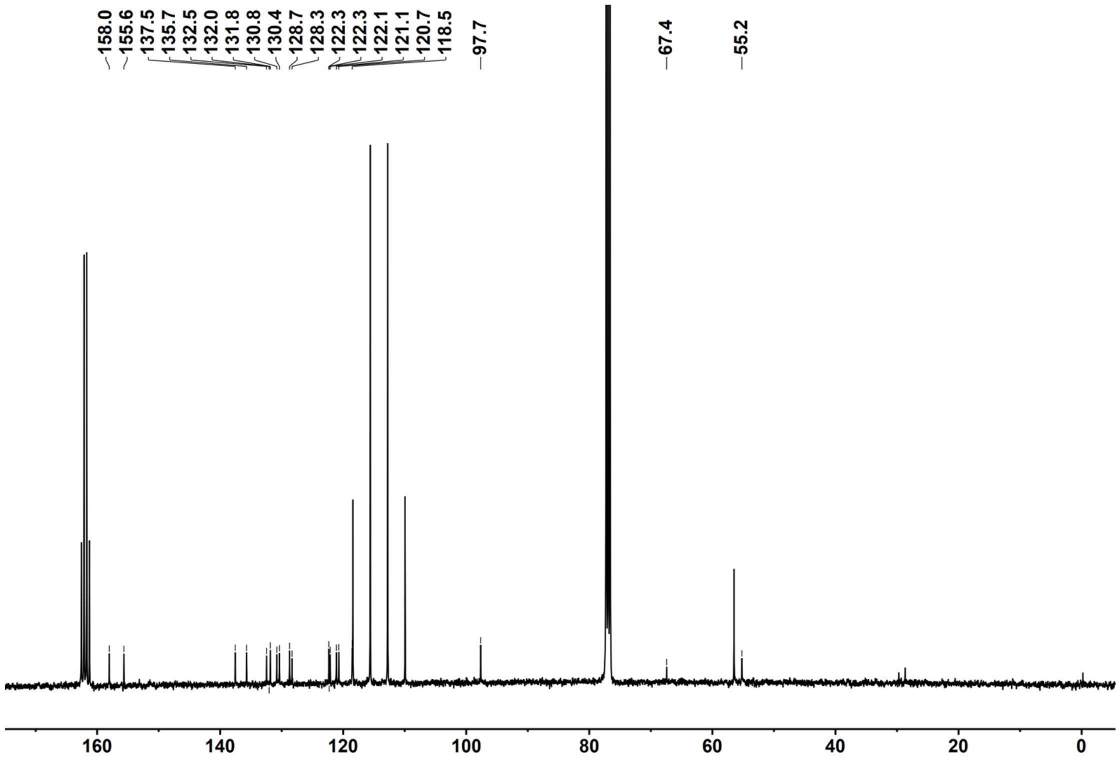

Figure S9. ^{13}C NMR spectrum (101 MHz, 298K) of ***meso*-TB[2]** in $CDCl_3$ with a small amount of TFA.

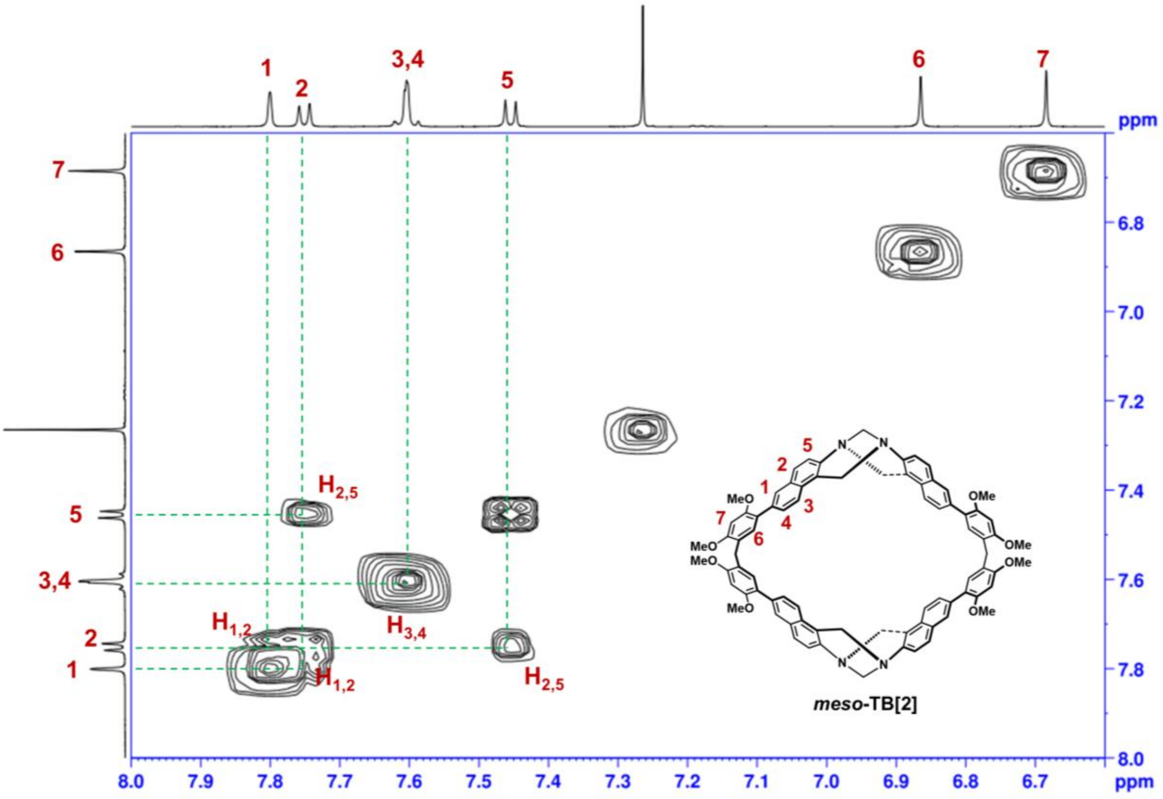

Figure S10. 2D ^{1}H-^{1}H COSY spectrum (600 MHz, 298 K) of ***meso*-TB[2]** in $CDCl_3$ with a small amount of TFA.

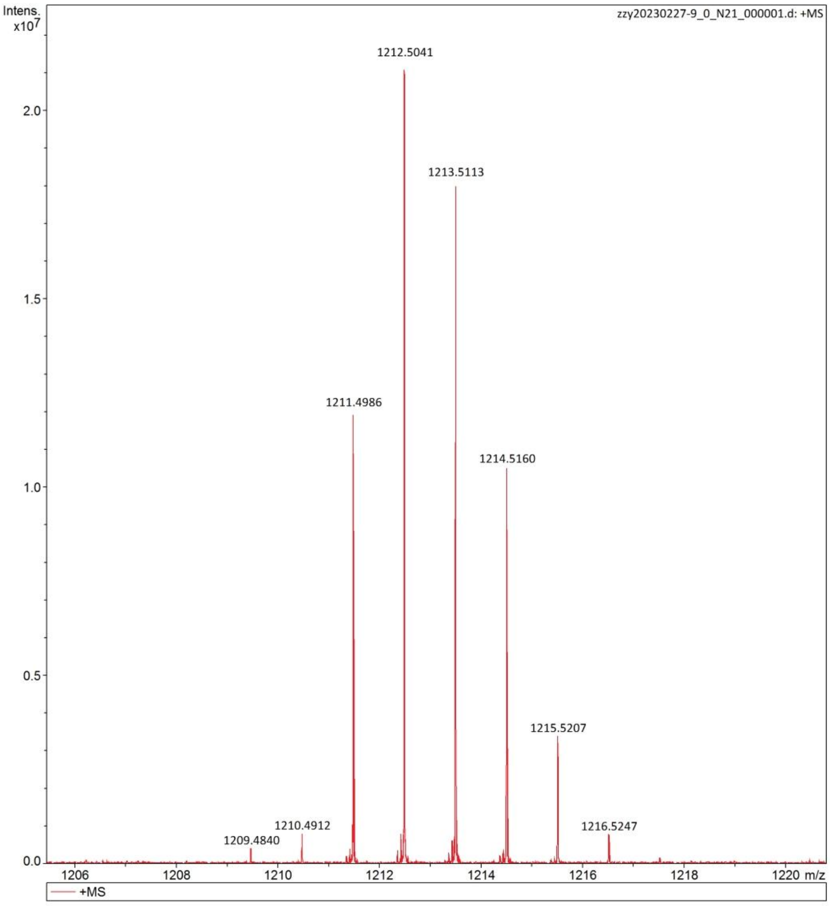

Figure S11. HRMS of ***meso*-TB[2]**.

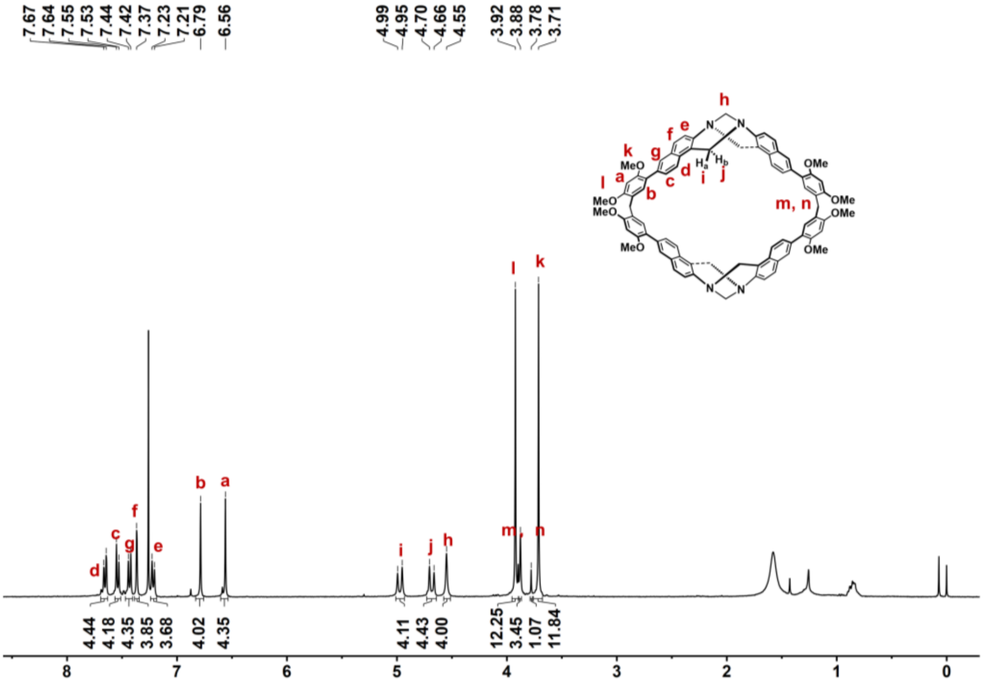

Figure S12. ^{1}H NMR spectrum (400 MHz, $CDCl_3$, 298K) of ***race*-TB[2]**.

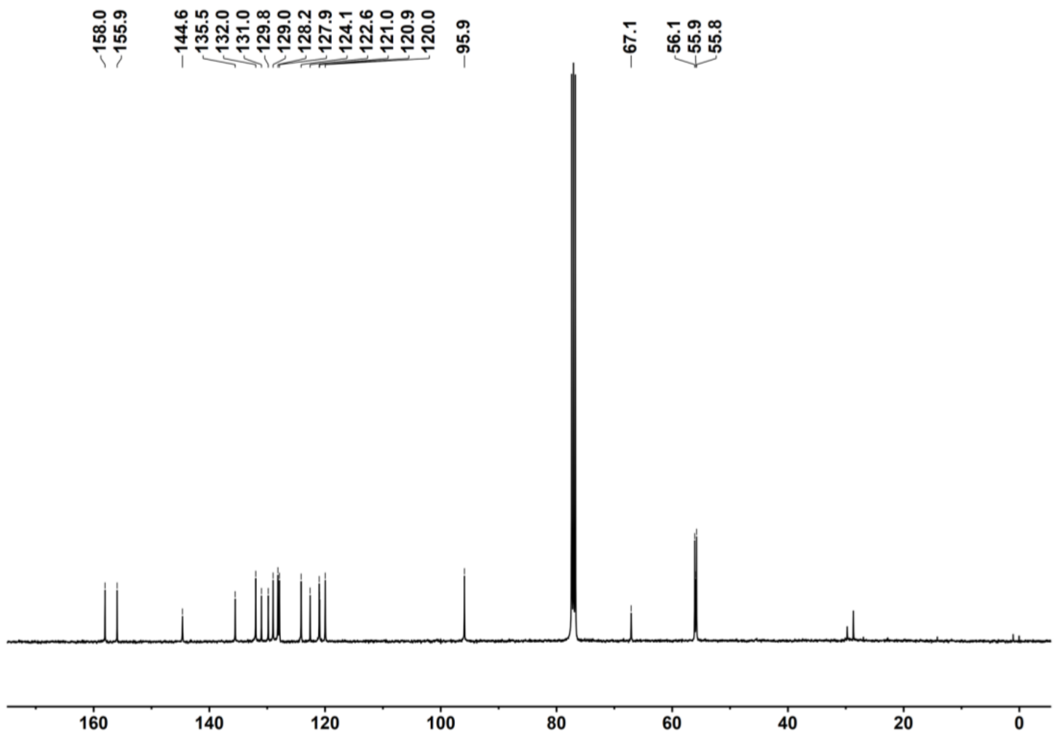

Figure S13. ^{13}C NMR spectrum (101 MHz, $CDCl_3$, 298K) of ***race*-TB[2]**.

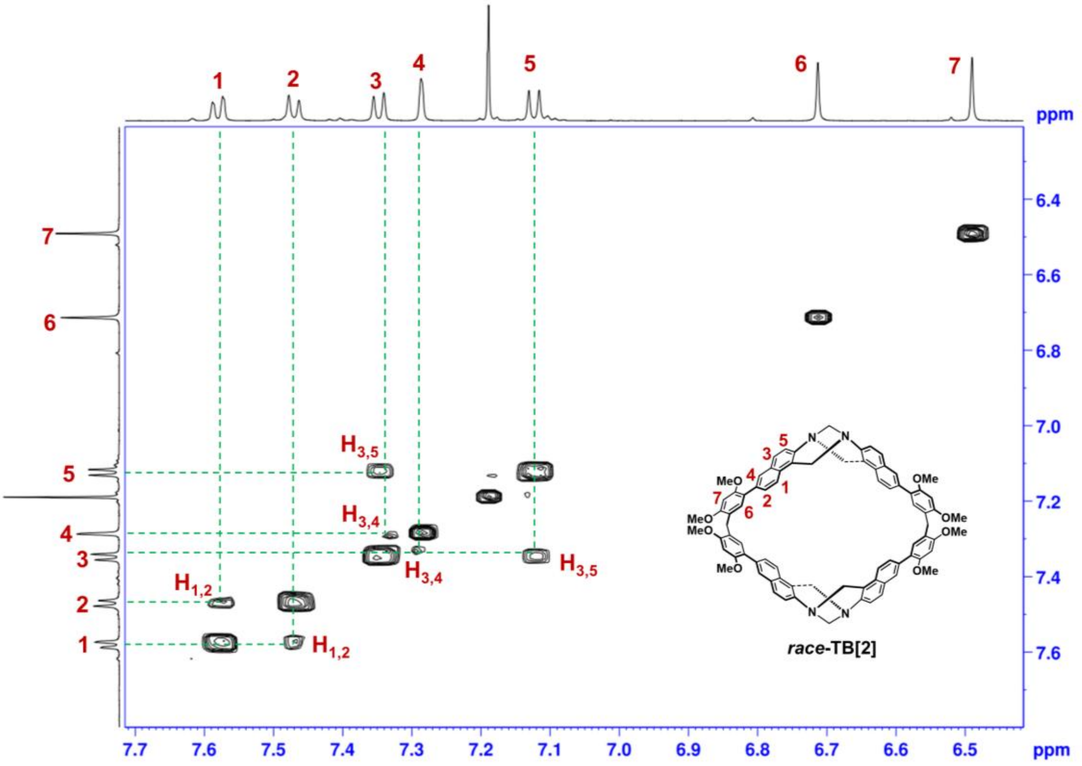

Figure S14. 2D ^{1}H-^{1}H COSY spectrum (600 MHz, 298 K) of *race*-TB[2] in $CDCl_3$.

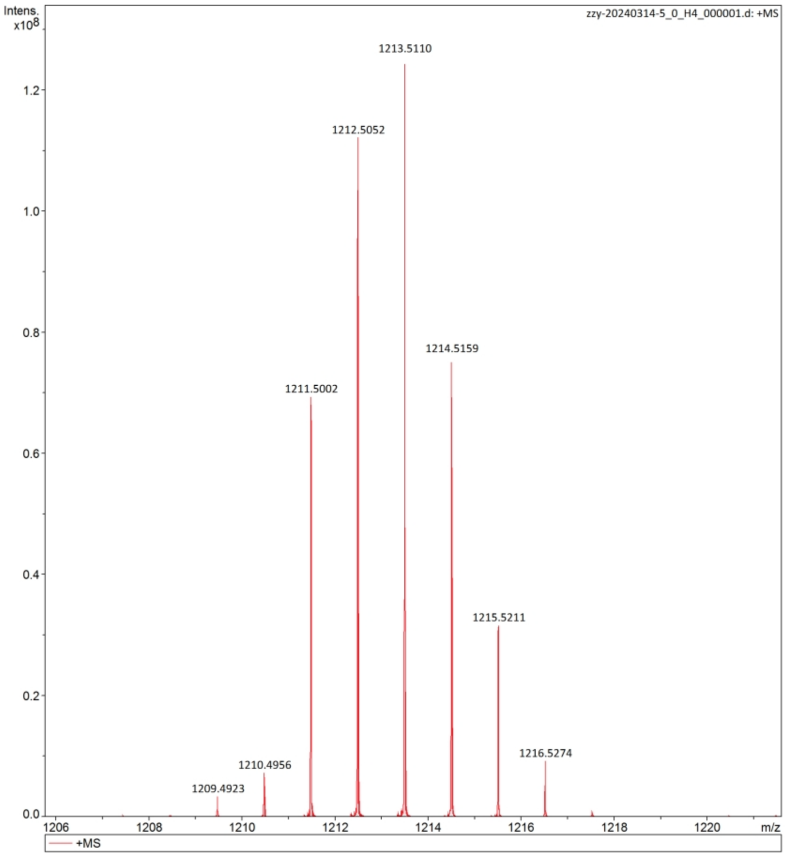

Figure S15. HRMS of ***race*-TB[2]**.

1.4 Single-crystal structure of TBM-2

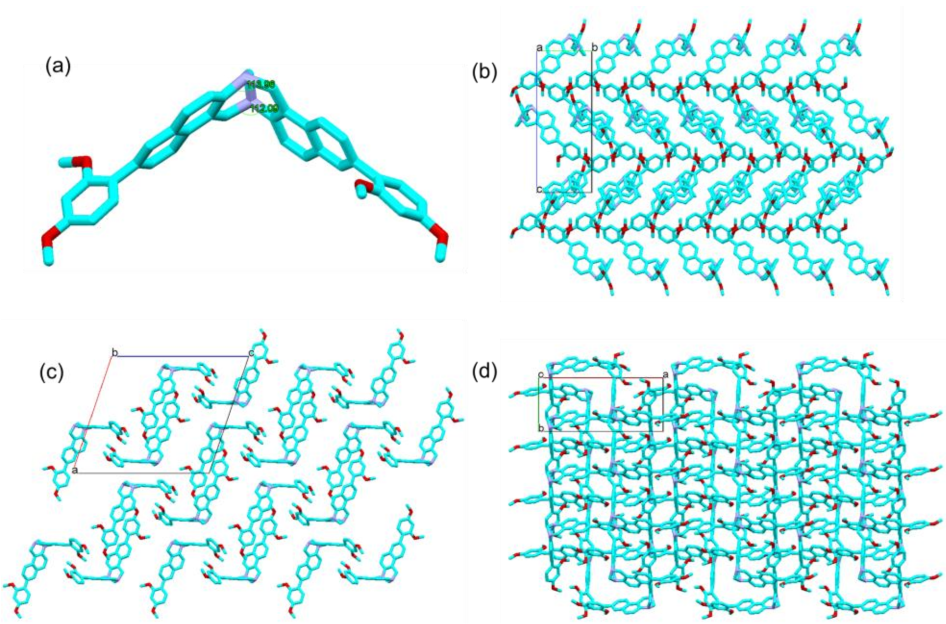

Figure S16. (a) Single-crystal structure of **TBM-2**. (b–d) The stacking structure of **TBM-2** along the *a*-, *b*- and *c*-axis. The hydrogen atoms and solvent molecules are removed for clarity.

Table S1. Crystal data and structure refinement for **TBM-2**.

Empirical formula	$C_{47}H_{50}N_2O_8$
Formula weight	770.89
Temperature/K	298.00
Crystal system	monoclinic
Space group	$P2_1/n$
a/Å	21.0412(5)
b/Å	8.5903(3)
c/Å	23.3311(7)
α/°	90
β/°	108.710(2)
γ/°	90
Volume/Å^3	3994.2(2)
Z	4
ρ_{calc}g/cm^3	1.282
μ/mm^{-1}	0.705
F(000)	1640.0
Crystal size/mm^3	0.04 × 0.03 × 0.03
Radiation	Cu Kα (λ = 1.54178)
2Θ range for data collection/°	4.926 to 144.326
Index ranges	-19 ≤ h ≤ 25, -10 ≤ k ≤ 10, -28 ≤ l ≤ 28
Reflections collected	39785
Independent reflections	7819 [R_{int} = 0.0656, R_{sigma} = 0.0389]
Data/restraints/parameters	7819/0/518
Goodness-of-fit on F^2	0.937
Final R indexes [I>=2σ (I)]	R_1 = 0.0870, wR_2 = 0.2850
Final R indexes [all data]	R_1 = 0.1130, wR_2 = 0.3302
Largest diff. peak/hole / e Å^{-3}	0.58/-0.57
**CCDC number**	**2394473**

1.5 Single crystal structure of race-TB[2]

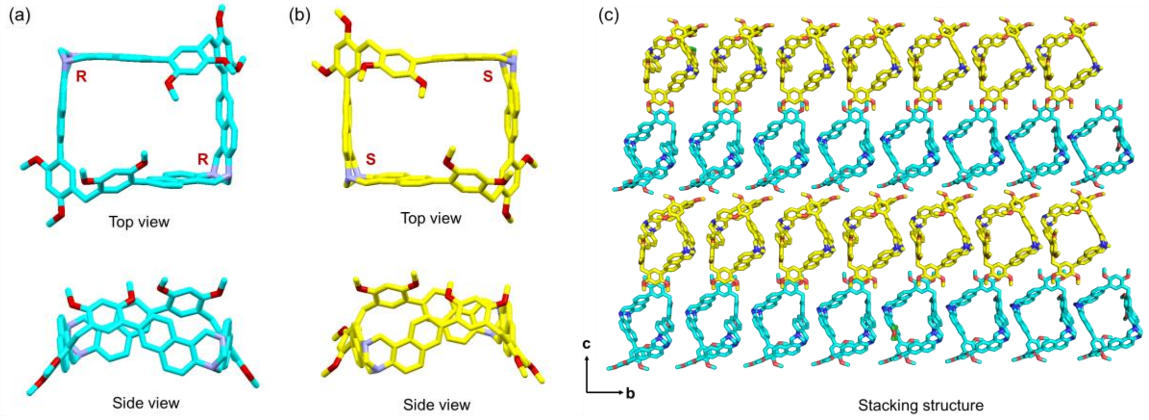

Figure S17. (a, b) Single-crystal structure of ***race*-TB[2]** at top view and side view. (c) The stacking structure of ***race*-TB[2]** along the *a* axis. The hydrogen atoms and solvent molecules are removed for clarity.

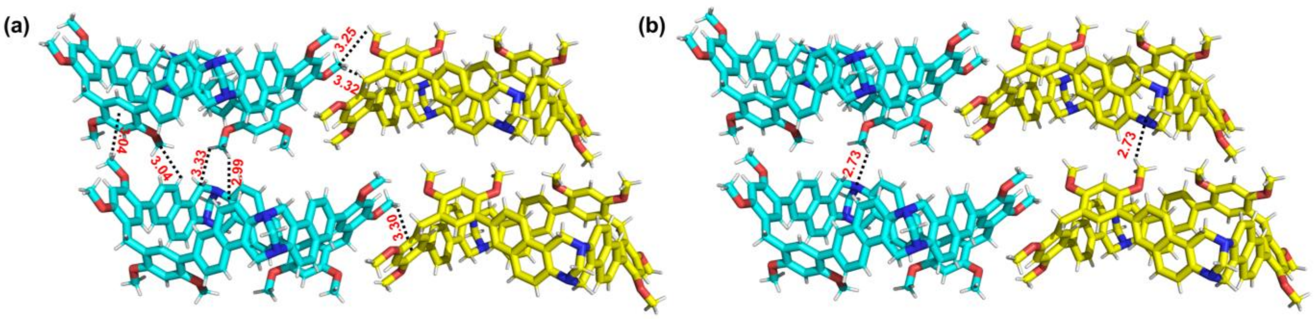

Figure S18. The C–H···π (a) and C–H···N (b) interactions between ***race*-TB[2]**.

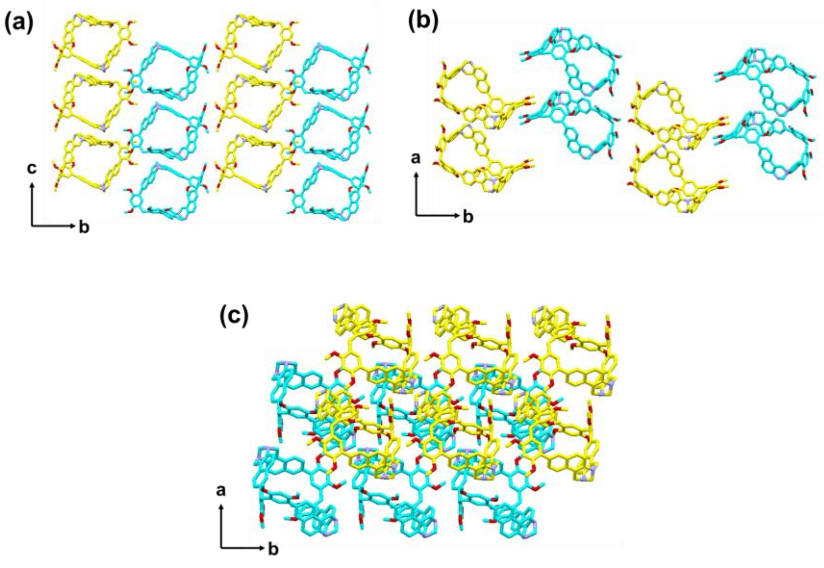

Figure S19. The stacking structures of ***race*-TB[2]** along the *a-, b-* and *c*-axis. The hydrogen atoms and solvents are removed for clarity.

Table S2. Crystal data and structure refinement for ***race*-TB[2]**.

Empirical formula	$C_{124}H_{156}N_4O_{30}$
Formula weight	2182.52
Temperature/K	150.00
Crystal system	triclinic
Space group	*P*-1
a/Å	11.9445(4)
b/Å	13.5729(4)
c/Å	36.2072(11)
α/°	89.204(2)
β/°	84.896(2)
γ/°	78.610(2)
Volume/Å^3	5731.5(3)
Z	2
ρ_{calc}g/cm^3	1.265
μ/mm^{-1}	0.735
F(000)	2336.0
Crystal size/mm^3	0.21 × 0.18 × 0.1
Radiation	Cu Kα (λ = 1.54178)
2Θ range for data collection/°	4.9 to 133.188
Index ranges	$-14 \le h \le 13$, $-16 \le k \le 16$, $-43 \le l \le 43$
Reflections collected	61608
Independent reflections	20177 [R_{int} = 0.0494, R_{sigma} = 0.0818]
Data/restraints/parameters	20177/1369/1704
Goodness-of-fit on F^2	1.188
Final R indexes [I>=2σ (I)]	R_1 = 0.1015, wR_2 = 0.2938
Final R indexes [all data]	R_1 = 0.1290, wR_2 = 0.3357
Largest diff. peak/hole / e Å^{-3}	0.69/-0.55
**CCDC number**	**2385605**

2. Host-guest complexation studies

2.1 Host-guest complexation in solution

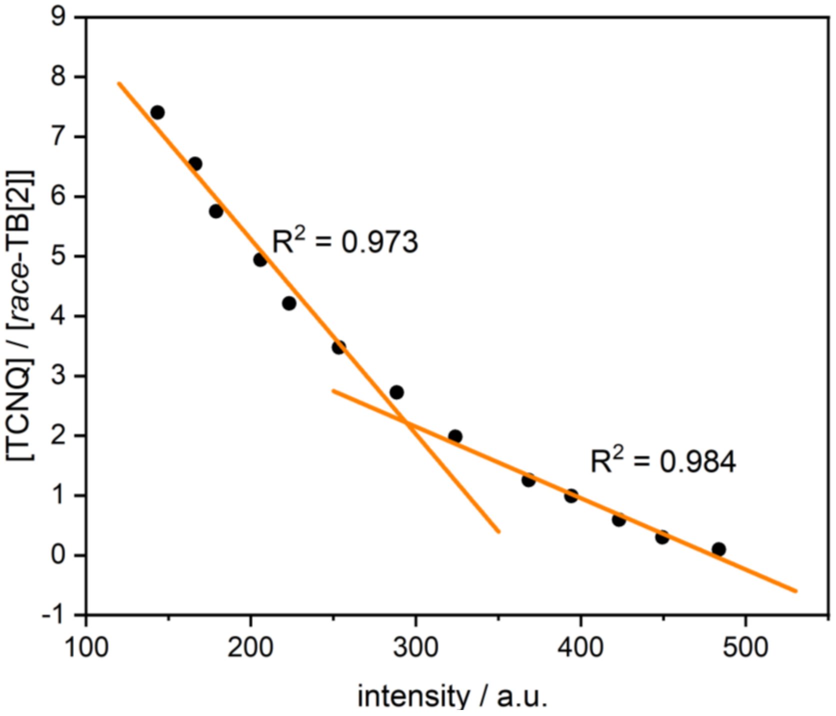

Figure S20. Mole ratio plot for ***race*-TB[2]** and TCNQ from fluorescence experiment, wherein ***race*-TB[2]** (at a fixed concentration) in $CHCl_3$ was treated different molar equivalents of TCNQ. The results are consistent with a 1 : 2 binding stoichiometry.

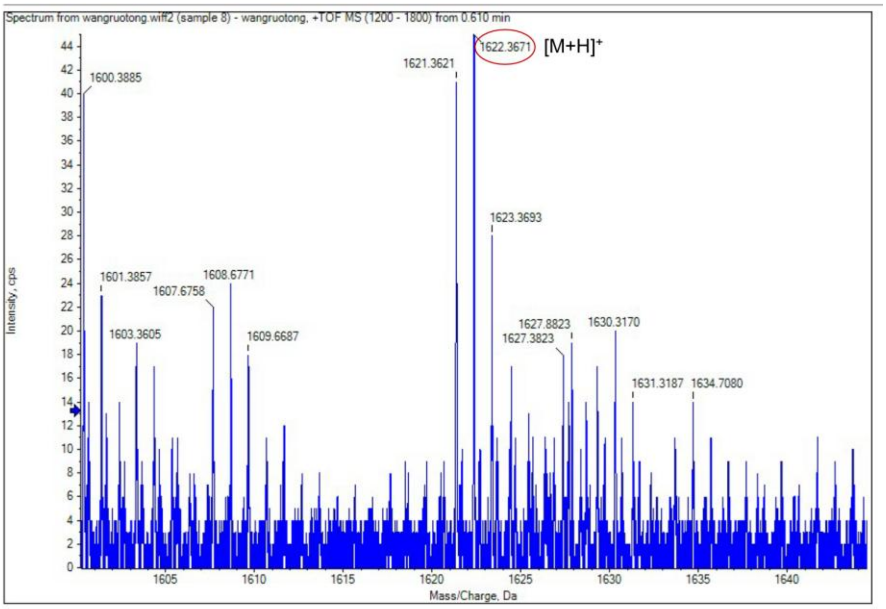

Figure S21. HRMS of *race*-TB[2] and TCNQ complex, indicating a 1:2 stoichiometric ratio.

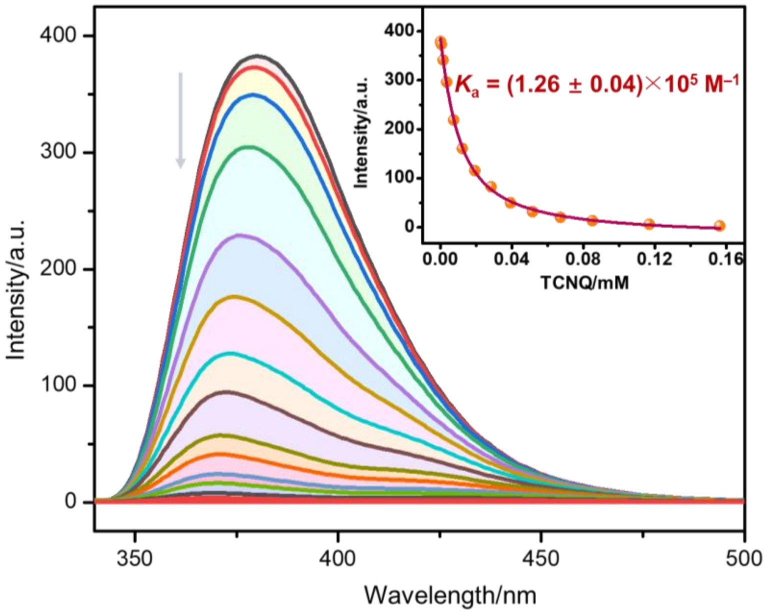

Figure S22. Direct fluorescence titration of ***race*-TB[2]** (5 μM) with TCNQ in $CHCl_3$. Inset: the associated titration curve at λ_{em} = 383 nm fitted according to a 1:2 host-guest equivalent binding site model.

2.2 Host-guest complexation in the solid state

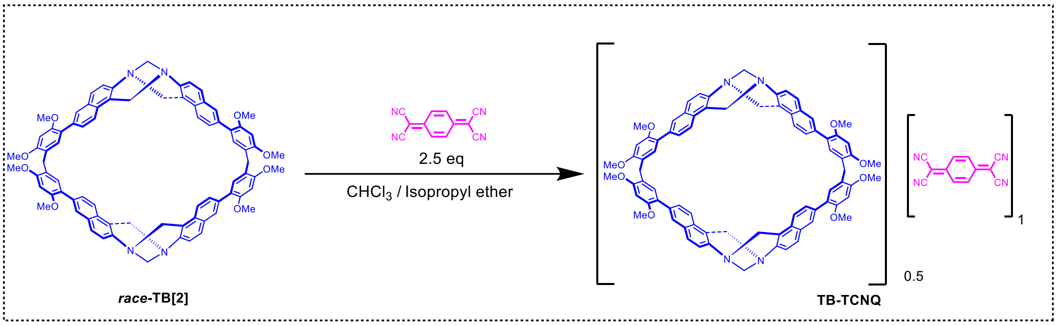

Figure S23. Co-crystallization of ***race*-TB[2]** with TCNQ in $CHCl_3$ to afford TB-TCNQ.

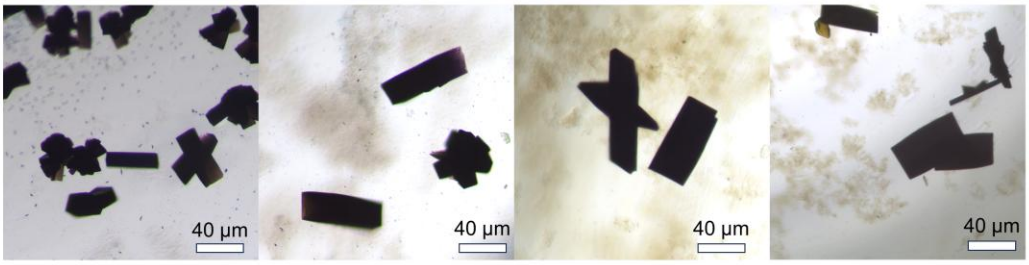

Figure S24. Optical images of TB-TCNQ. Scale bar: 40 μm.

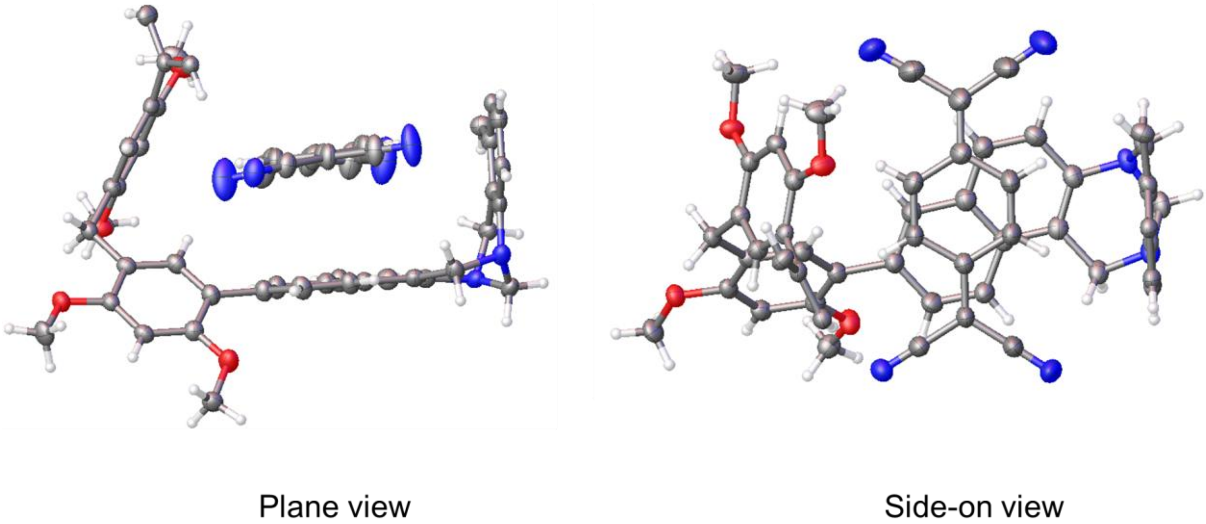

Figure S25. ORTEP drawing of TB-TCNQ with 0.5 *race*-TB[2] and one TCNQ molecules in the asymmetric unit from plane view and side-on view. The thermal ellipsoids are displayed at a 50% probability. The solvents are omitted for clarity.

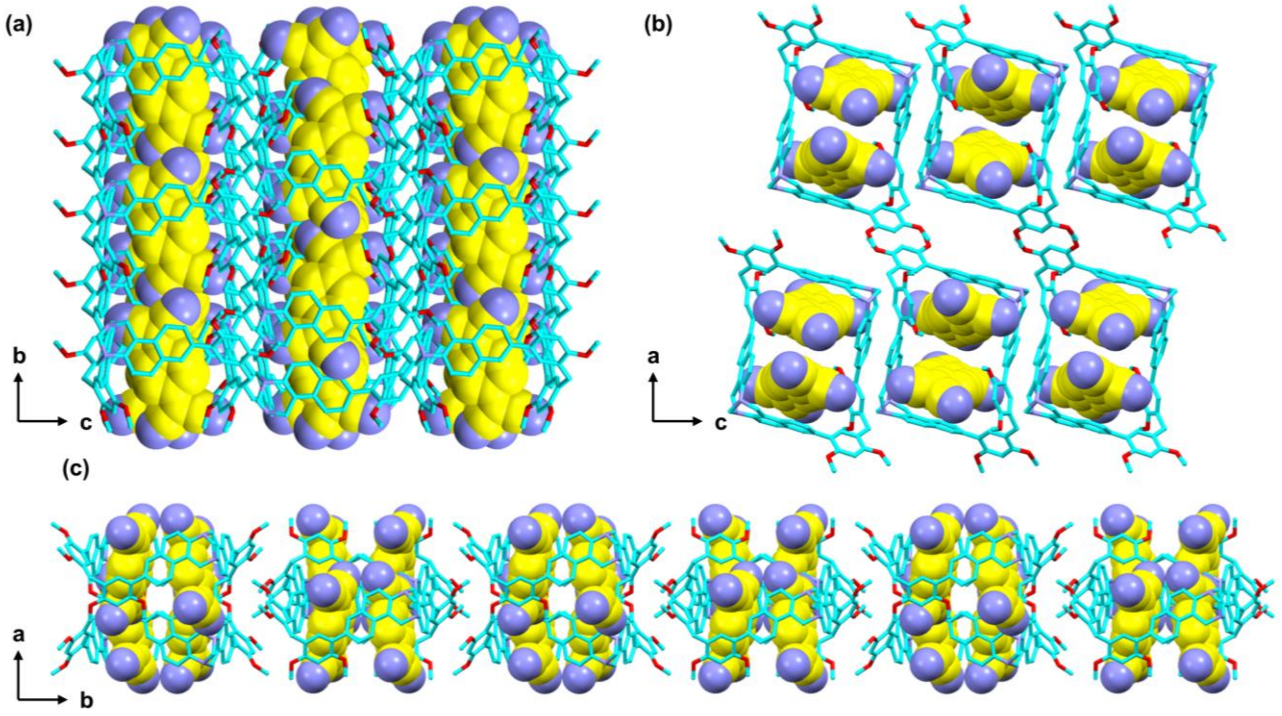

Figure S26. (a–c) Stacking structures of TB-TCNQ in the solid state along the *a*-, *b*- and *c*- axis.

Table S3. Crystal data and structure refinement for TB-TCNQ.

Identification code	TB-TCNQ
Empirical formula	$C_{104}H_{76}N_{12}O_8$
Formula weight	1621.76
Temperature/K	100.01(10)
Crystal system	monoclinic
Space group	C2/c
a/Å	36.7411(6)
b/Å	10.3690(2)
c/Å	23.8093(3)
α/°	90
β/°	99.9080(10)
γ/°	90
Volume/Å^3	8935.3(3)
Z	4
ρ_{calc}g/cm^3	1.206
μ/mm^{-1}	0.623
F(000)	3392.0
Crystal size/mm^3	0.17 × 0.16 × 0.15
Radiation	Cu *K*α (λ = 1.54184)
2Θ range for data collection/°	4.884 to 151.176
Index ranges	$-46 \le h \le 45$, $0 \le k \le 12$, $0 \le l \le 29$
Reflections collected	9050
Data/restraints/parameters	9050/0/564
Goodness-of-fit on F^2	1.065
Final R indexes [I>=2σ (I)]	R_1 = 0.0879, wR_2 = 0.2661
**CCDC number**	**2385606**

3. Photophysical Properties of TB-TCNQ

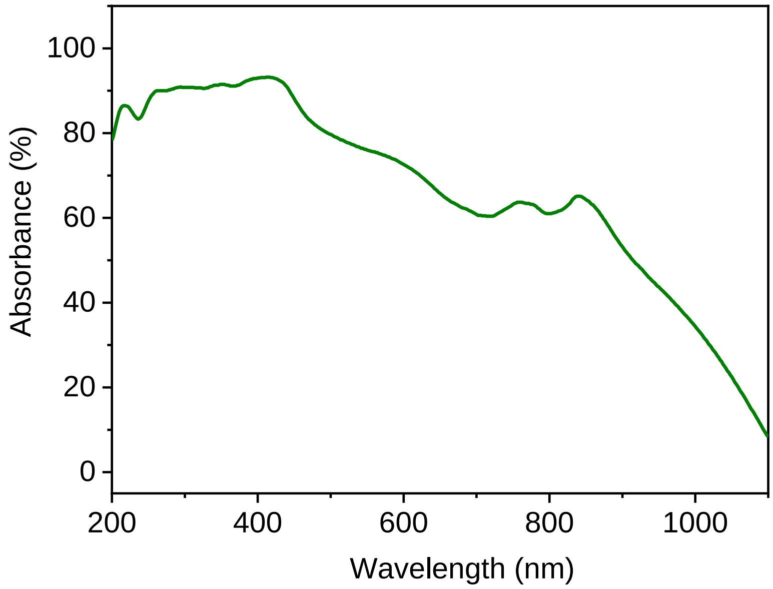

Figure 27. Absorption spectrum of TB-TCNQ co-crystals.

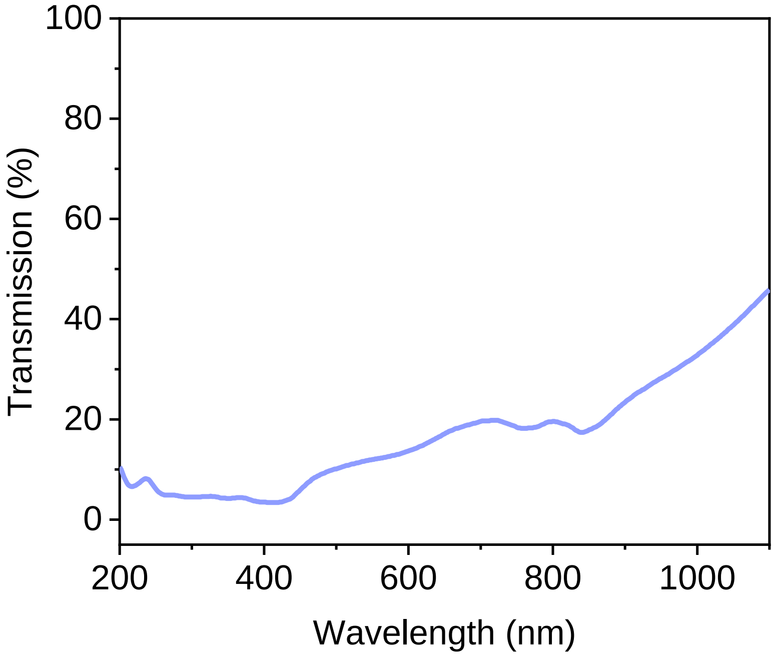

Figure 28. Transmission spectrum of TB-TCNQ co-crystals.

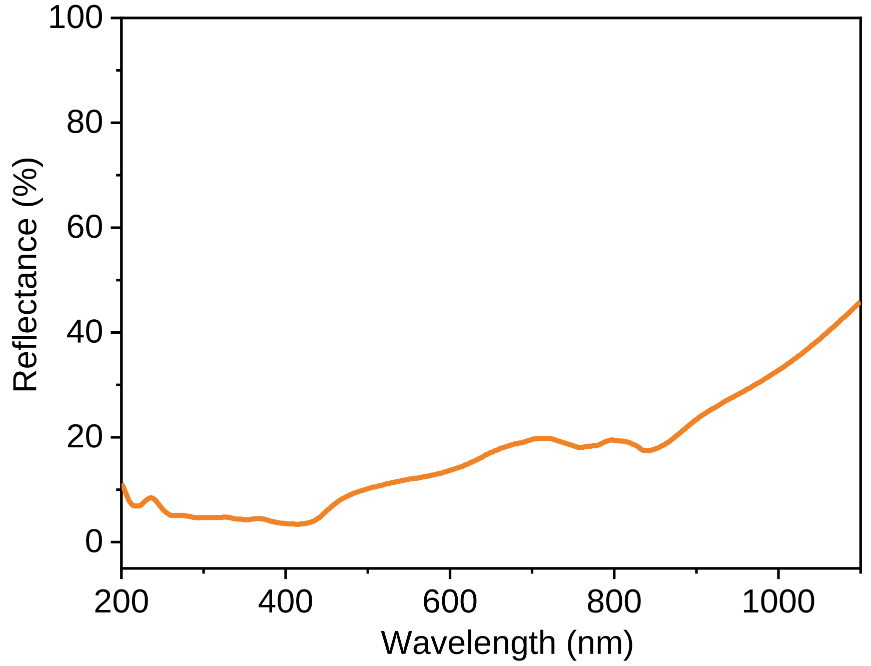

Figure 29. Reflectance spectrum of TB-TCNQ co-crystals.

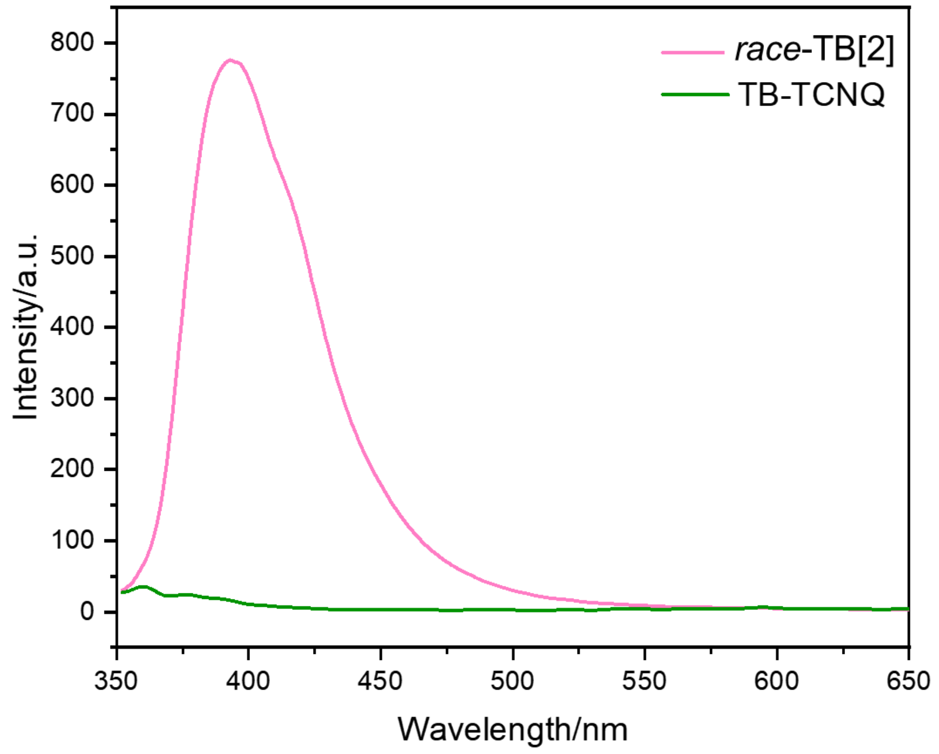

Figure S30. Solid-state fluorescence spectra of *race*-TB[2] and TB-TCNQ, showing the fluorescence of *race*-TB[2] is completely quenched by the co-crystallization.

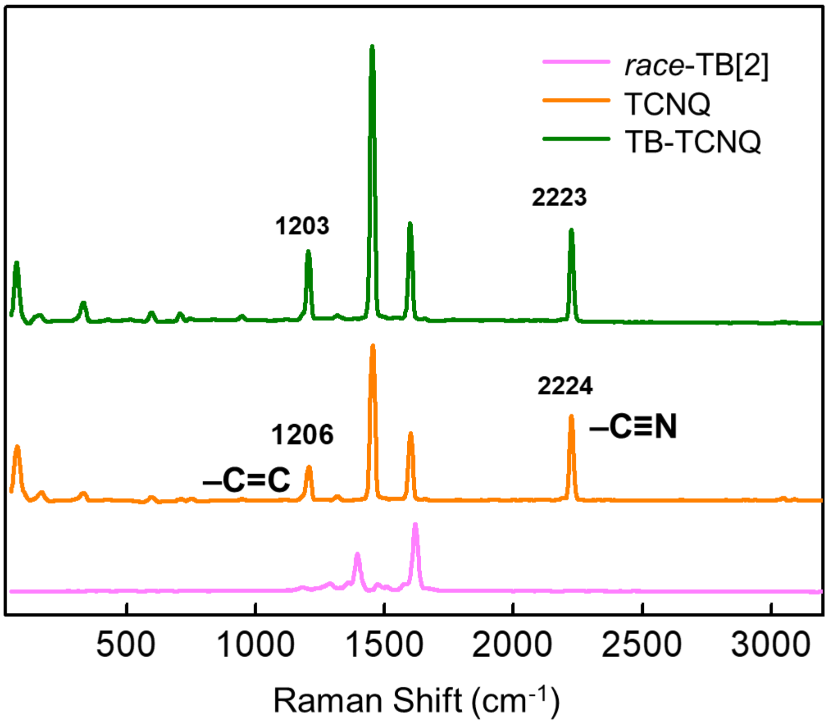

Figure S31. Raman spectra of *race*-TB[2], TCNQ and TB-TCNQ.

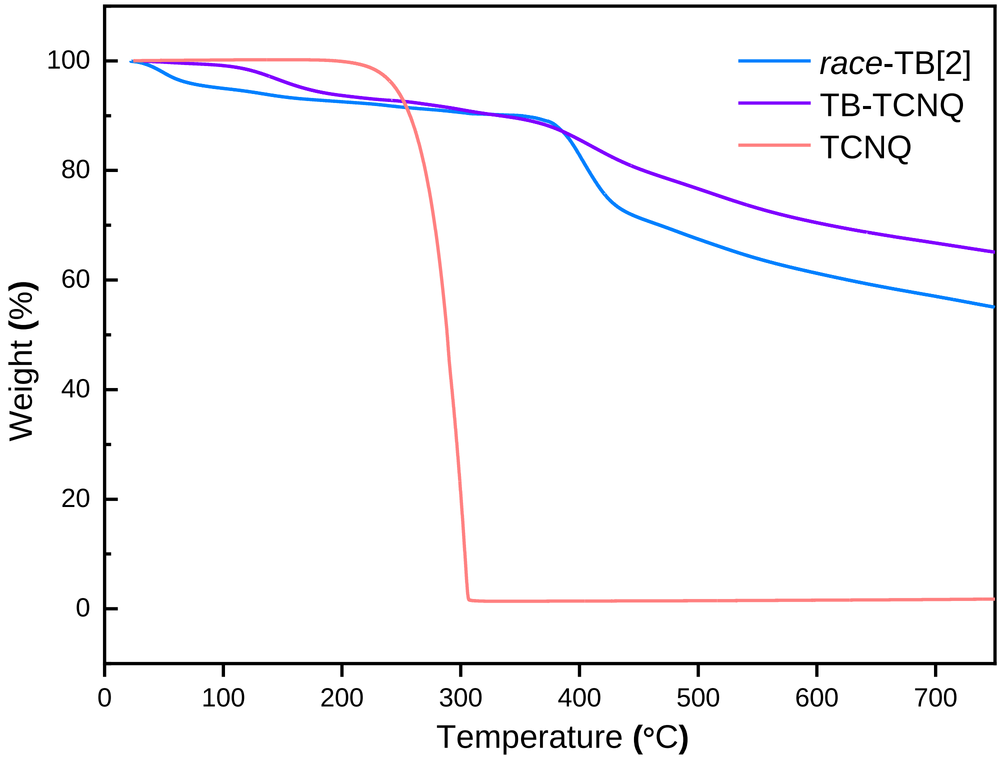

Figure S32. TGA curves of *race*-TB[2], TCNQ and TB-TCNQ.

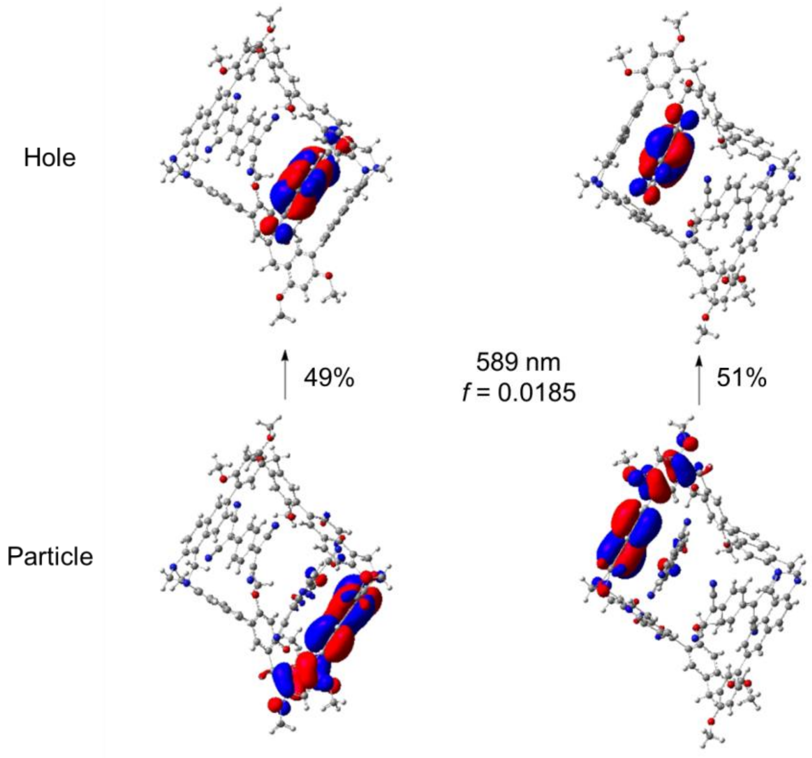

Figure S33. The dominant natural transition orbital pairs for the first excited singlet state (the absorption peak is at 589 nm, and oscillator strength value is 0.0185). The "hole" is on the top, and the "particle" is on the bottom. The DFT and TDDFT calculation were carried out at CAM-B3LYP/6-311G* level using the Gaussian 16 suite.

4. Photothermal Conversion Studies

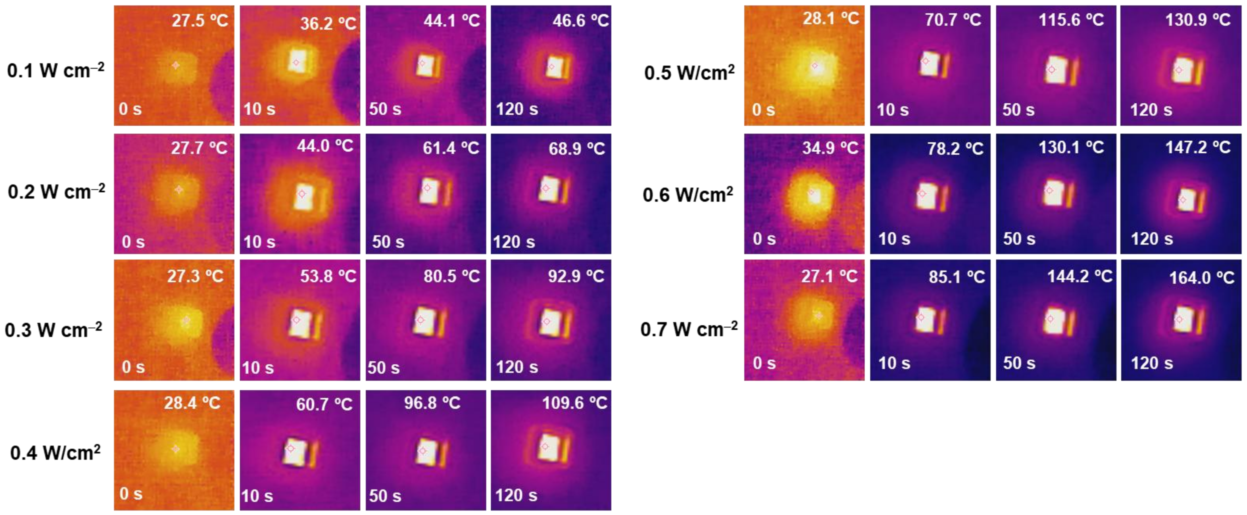

Figure S34. The photothermal imaging process of TB-TCNQ under 808 nm laser irradiation at 0.1–0.7 W cm^{-2}.

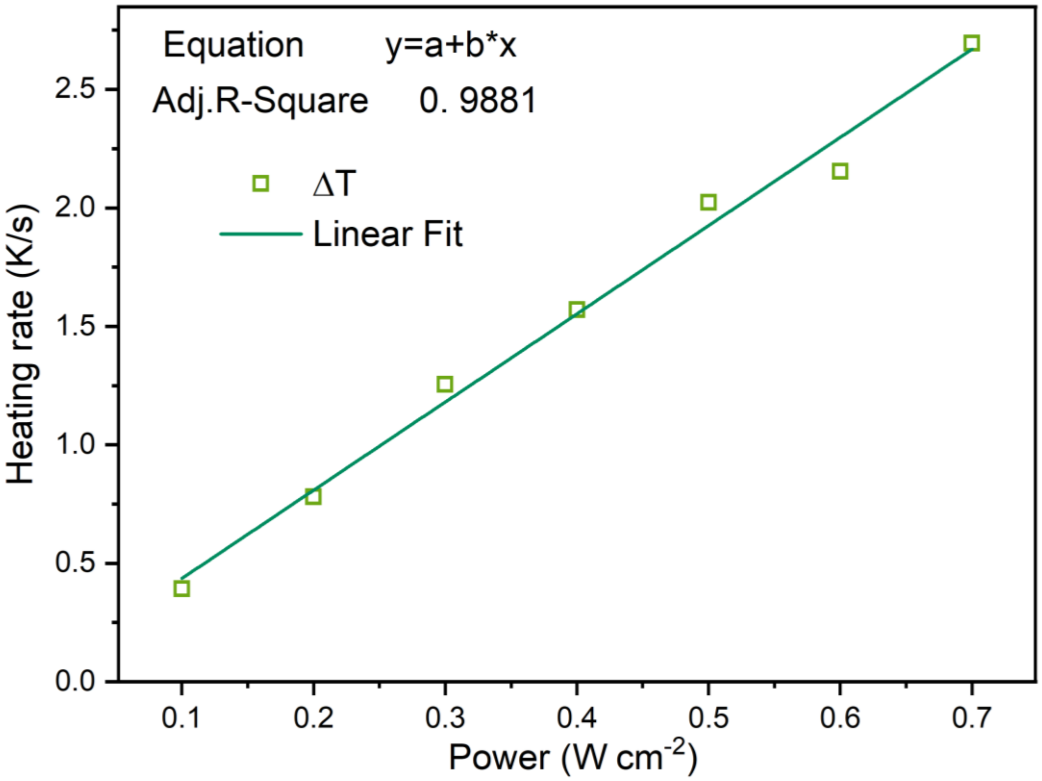

Figure S35. Photothermal heating rate of TB-TCNQ co-crystals as a function of laser power densities.

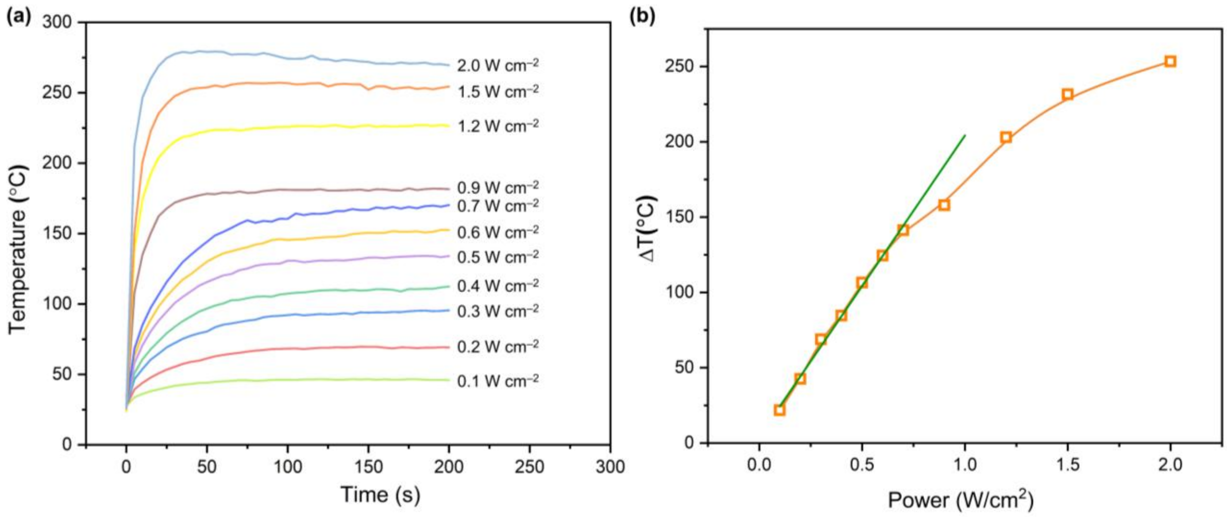

Figure S36. (a) The heating curves of TB-TCNQ under various powers (0.1–2.0 W cm^{-2}) of 808 nm laser. (b) The relationship between ΔT and laser power (0.1–2.0 W cm^{-2}) for TB-TCNQ.

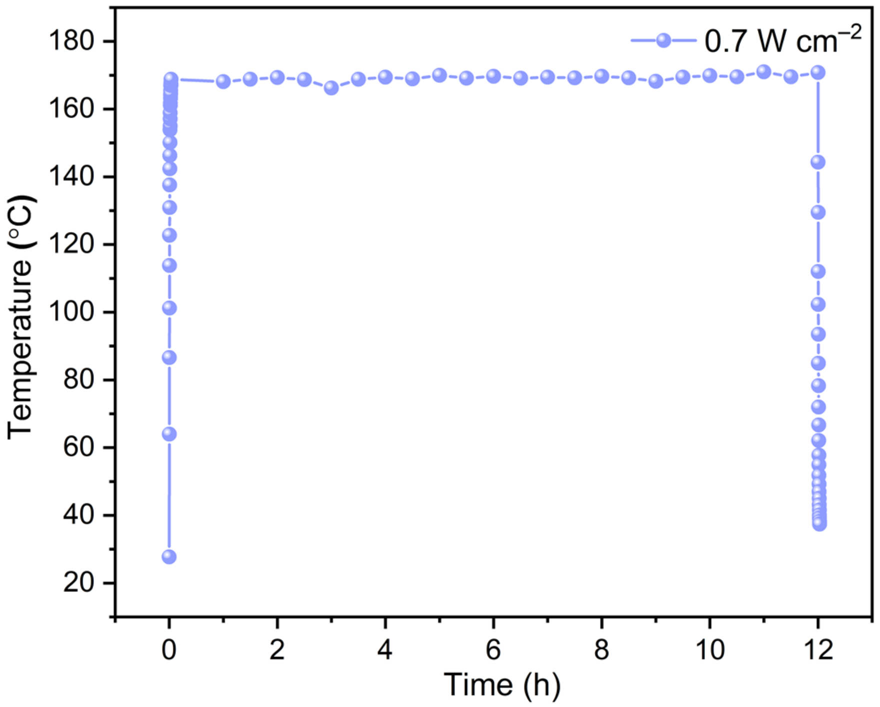

Figure S37. Heating and cooling curve of TB-TCNQ under continuous laser irradiation (0.7 W cm^{-2}) for 12 h.

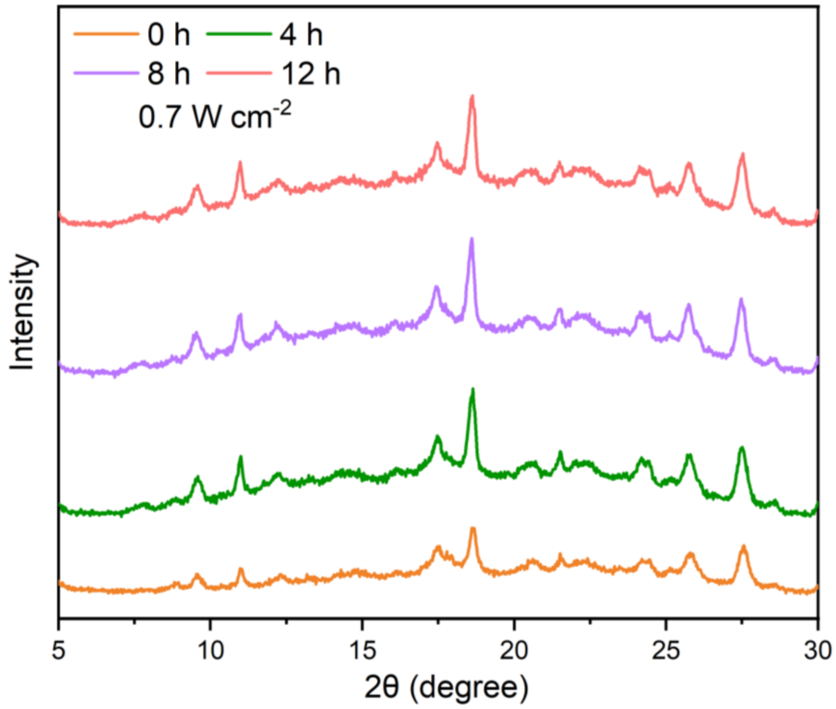

Figure S38. Powder X-ray diffraction spectra of TB-TCNQ before and after continuous laser irradiation (0.7 W cm^{-2}) over 12 h.

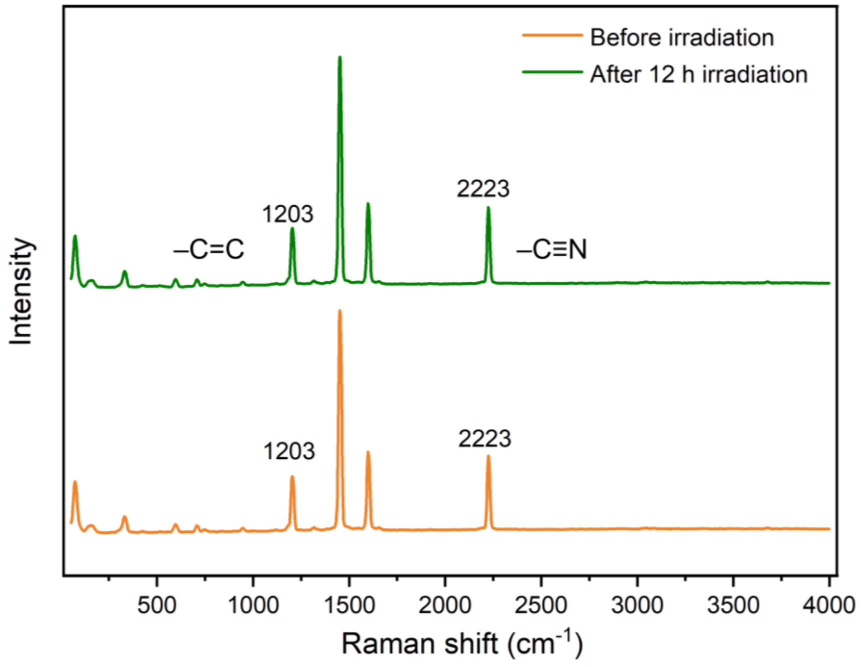

Figure S39. Raman spectra of TB-TCNQ before and after continuous laser irradiation (0.7 W cm^{-2}) over 12 h.

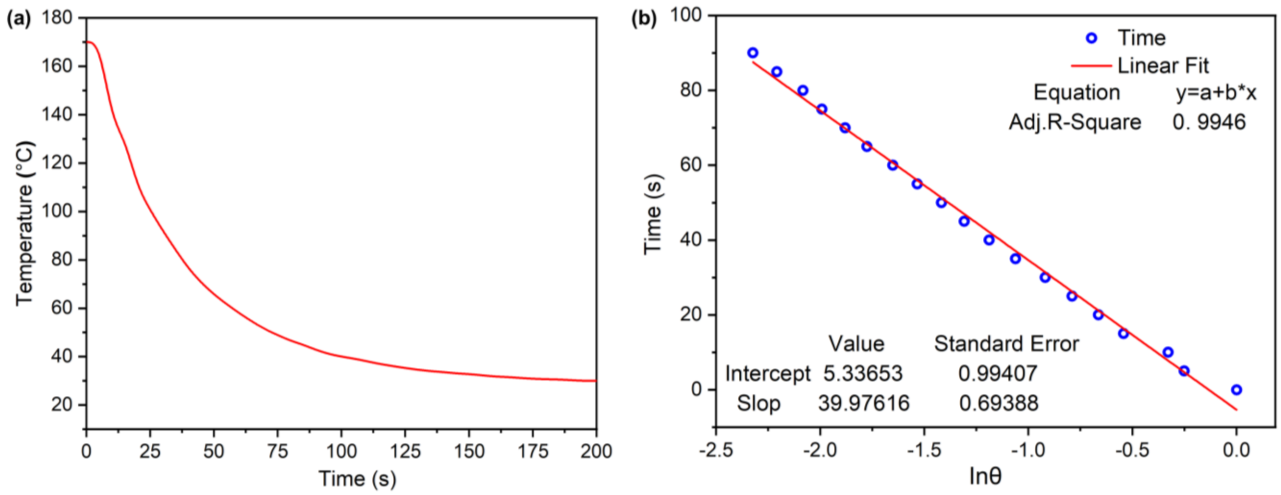

Figure S40. (a) The cooling curve of TB-TCNQ sample after 808 nm laser irradiation at 0.7 W cm^{-2}. (b) The corresponding time-Inθ linear curve.

Calculation of the photothermal conversion efficiency

The TB-TCNQ cocrystals were irradiated by NIR laser (808 nm) with a power density of 0.7 W cm^{-2} (DL-S-808-3000 mW, China) for 235 s. Then, the system cools naturally and the temperature change is recorded by IR thermal camera (HM-TPK20-3AQF/W, China). The photothermal conversion efficiency (PCE, η) of TB-TCNQ cocrystals can be calculated according to the previous method[S1] Details are as follows: Based on the total energy balance for this system:

$$\sum_i m_i C_{p,i} \frac{dT}{dt} = Q_s - Q_{loss}$$

Where m_i (95.0 mg) and $C_{p,i}$ (1.51 J g^{-1} K^{-1}) are the mass and heat capacity of co-crystal samples, respectively. Q_s is the photothermal heat energy input by irradiating NIR laser to co-crystal sample, and Q_{loss} is thermal energy lost to the surroundings. When the temperature is maximum, the system is in balance.

$$Q_s = Q_{loss} = hS\Delta T_{max}$$

Where h is heat transfer coefficient, S is the surface area of the samples, ΔT_{max} is the maximum temperature change. The photothermal conversion efficiency η is calculated from the following equation:

$$\eta = \frac{hS\Delta T_{max}}{I(1 - 10^{-A808})}$$

Where I is the laser power (0.7 W cm^{-2}) and $A808$ is the absorbance of the samples at the wavelength of 808 nm (0.66373).

In order to get the hS, a dimensionless driving force temperature, θ is introduced as follows:

$$\theta = \frac{T - T_{surr}}{T_{max} - T_{surr}}$$

Where T is the temperature of cocrystal, T_{max} is the maximum system temperature (171.2 °C), and T_{surr} is the initial temperature (27.1 °C). And a sample system time constant τ_s

$$\tau_S = \frac{\sum_i m_i C_{p,i}}{hS}$$

Thus, $\frac{d\theta}{dt} = \frac{1}{\tau_S}\frac{Q_S}{hS\Delta T_{max}} - \frac{\theta}{\tau_S}$

When the laser is off, $Q_S = 0$, therefore $\frac{d\theta}{dt} = -\frac{\theta}{\tau_S}$, and $t = -\tau_S \ln\theta$

So hS could be calculated from the slope of cooling time *vs* lnθ. Therefore, τ_s is 39.98 s (Figure S40b). And the photothermal conversion efficiency η is 94.33 %.

Table S4. The reported examples of PTC CT co-crystals

PT materials	Laser wavelength (nm)	PCE (%)	Ref.
**TB-TCNQ**	**808**	**94.3**	**This work**
DBTTF-TCNB	808	18.8	[S2]
CF	808	69.3	[S3]
TQC	1 Sun	90.3	[S4]
ATQ	808	60.5	[S5]
AFQ		57.0	
CZ/TCNQ	808	53.7	[S6]
ClCZ/TCNQ		48.5	
BrCZ/TCNQ		32.0	
TMB-TCNQ	1064	42.4	[S7]
TMB-4FTCNQ		48.0	
TMPD-PMDA	808	87.2	[S8]
T2C1	808	60.3	[S9]
T2C2		75.5	
MFC	1064	54.6	[S10]
MTC		36.8	
TMB-ABTS$^{+\bullet}$-H_2O	1064	49.6	[S11]
DBTTF/TCB	808	21.9	[S12]
ZnTPP/C60	808	31.7	[S13]
PER/TCNQ	1064	42	[S14]
PY/PEC	808	80.89	[S15]

Table S5. A comprehensive comparison between our system and other state-of-the-art PTC materials

Type	PTC materials	laser wavelength (nm)	PCE (%)	Practical application	Ref.
**Inclusion co-crystals**	**TB-TCNQ**	**808**	**94.3**	**water evaporation**	**This work**
Nanowire aerogel	Cu@PPy	1 sun	97.6	water evaporation	[S16]
Organic polymer	Polypyrrole nanosheets	1 sun	95.33	solar steam generation	[S17]
Cage-stabilized gold nanoparticles	AuNCs@Superphane	808	92.8	water evaporation	[S18]
MOF-isolated graphene	G@ZIF	1 sun	98	water evaporation	[S19]
Small organic molecules	BAF4 NPs	1064	80	Photothermal therapy	[S20]
Covalent-Organic Framework (COF)	TPAD-COF-BF_2	1 sun	94	water evaporation	[S21]

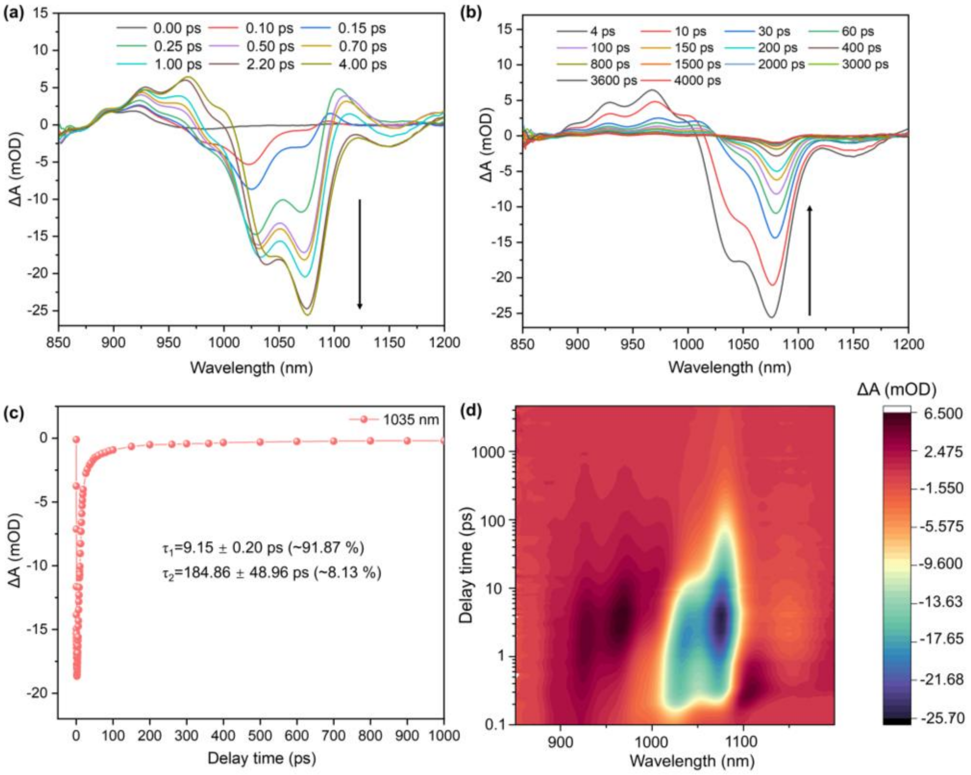

Figure S41. Fs-TA spectra of the TB-TCNQ in $CHCl_3$ (10 mg/200 μL) in the range of (a) 0 ps to 4ps and (b) 4 ps to 4000 ps. (c) Kinetic fitting results of the TB-TCNQ at 1035 nm. (d) Representative pseudocolor TA spectrum of TB-TCNQ.

5. Solar-driven water evaporation

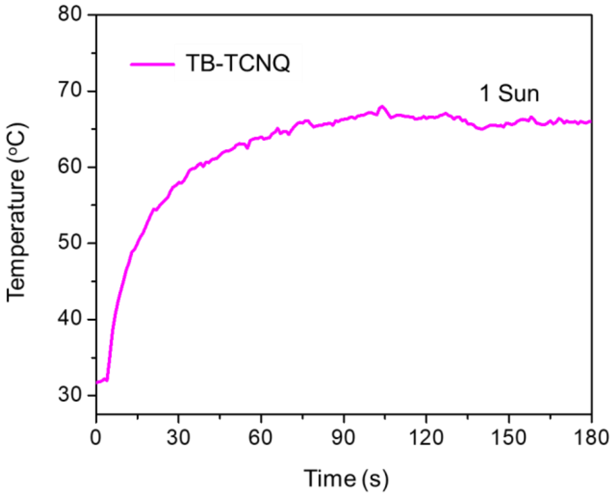

Figure S42. The temperature changes of TB-TCNQ under 1 Sun irradiation.

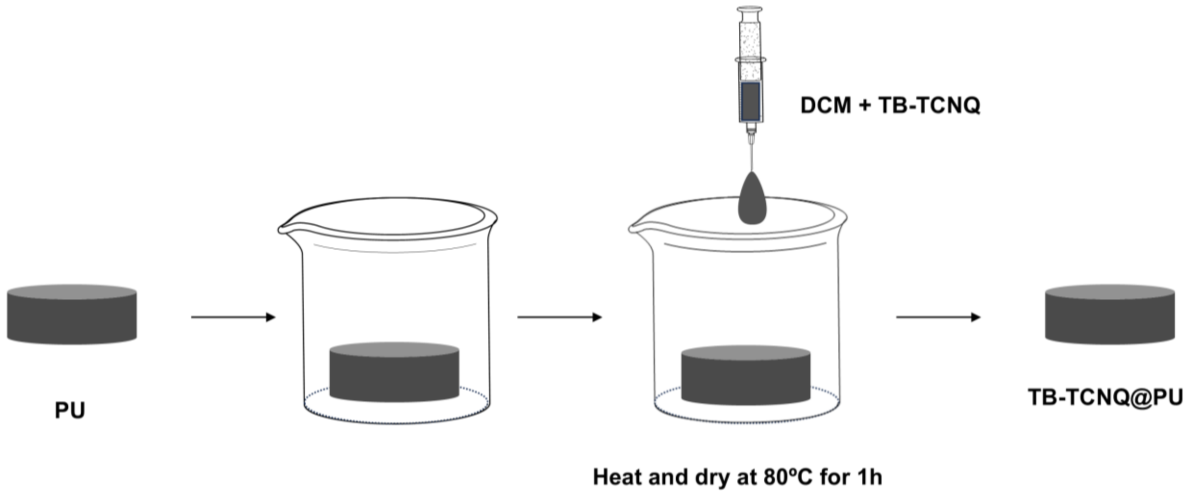

Figure S43. The schematic of the process for PU foam loading TB-TCNQ by impregnation method.

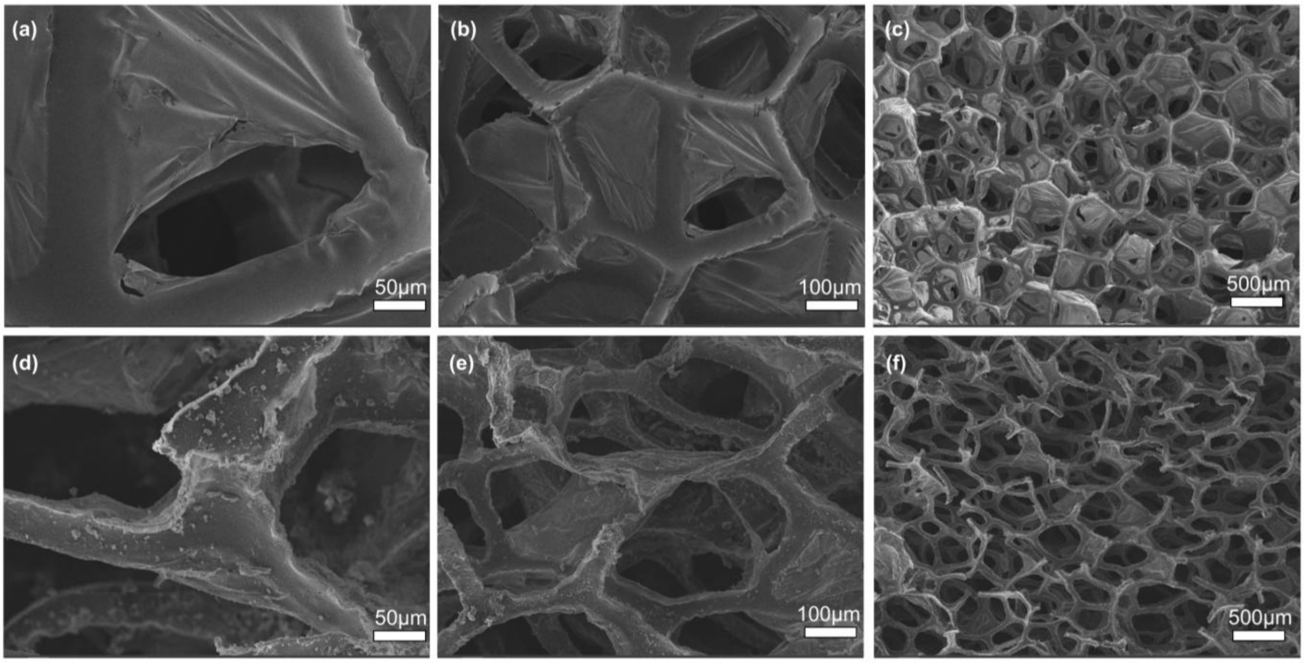

Figure S44. SEM images of blank PU foam (a–c) and TB-TCNQ@PU foam (d–f) with mass of TB-TCNQ loading in PU of 4.24 mg cm^{-3}.

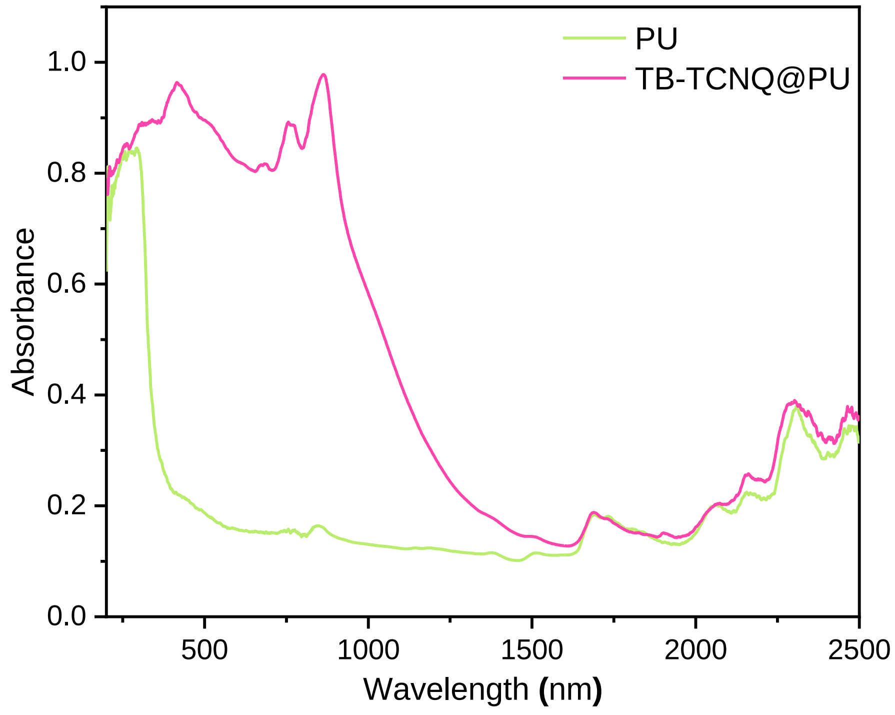

Figure S45. UV−vis−NIR absorption of PU and TB-TCNQ@PU foam.

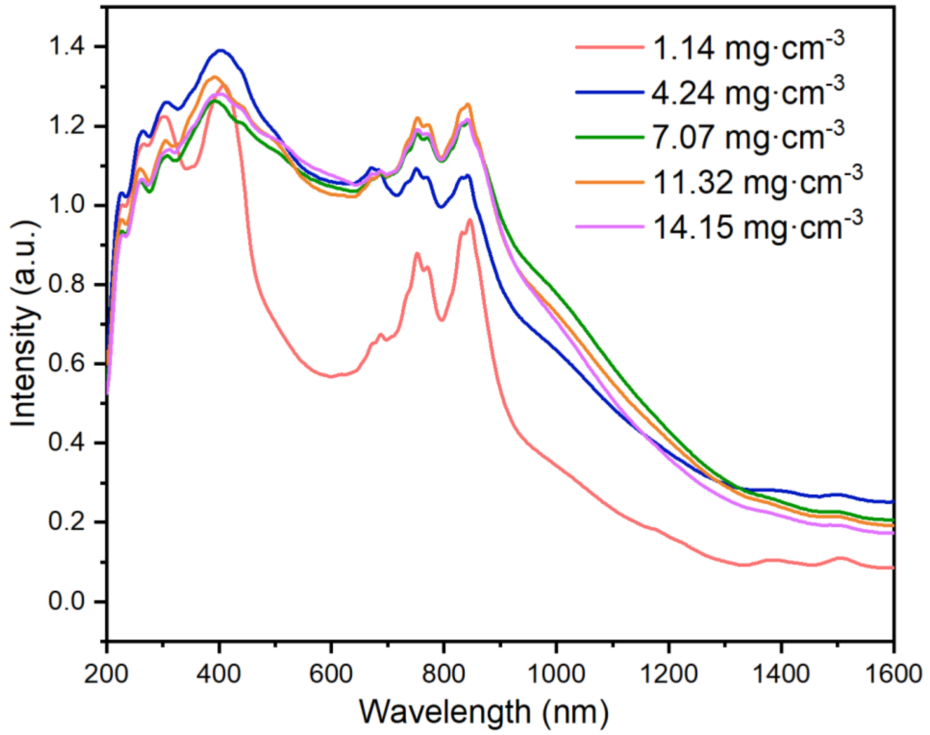

Figure S46. UV-Vis-NIR of TB-TCNQ@PU composites with different amount of TB-TCNQ loading in PU foams (1.41, 4.24, 7.07, 11.32, and 14.15 mg cm^{-3}).

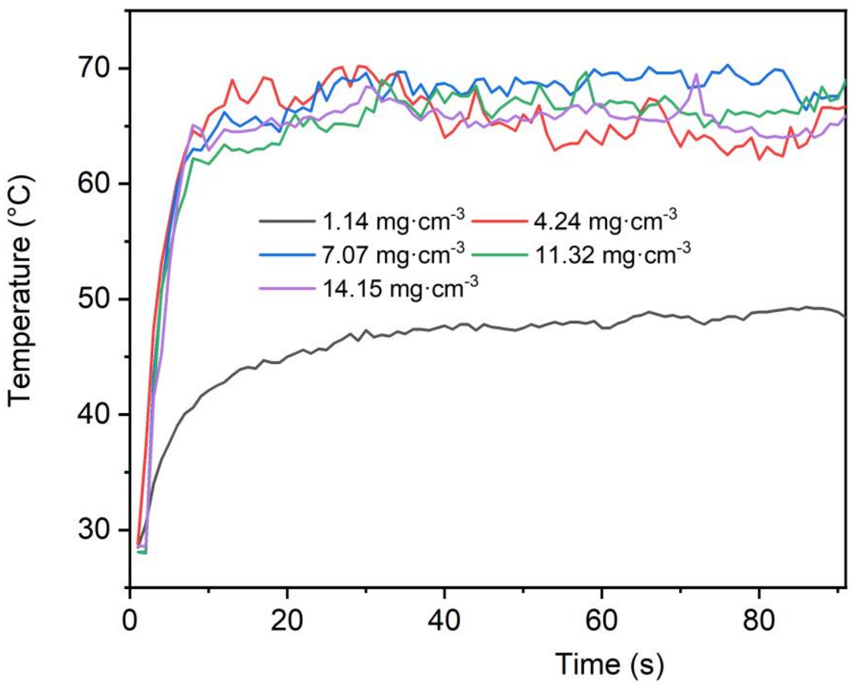

Figure S47. The temperature changes of TB-TCNQ@PU composites with different amount of TB-TCNQ loading in PU foams (0, 1.41, 4.24, 7.07, 11.32, and 14.15 mg cm^{-3}) under 0.5 Sun irradiation.

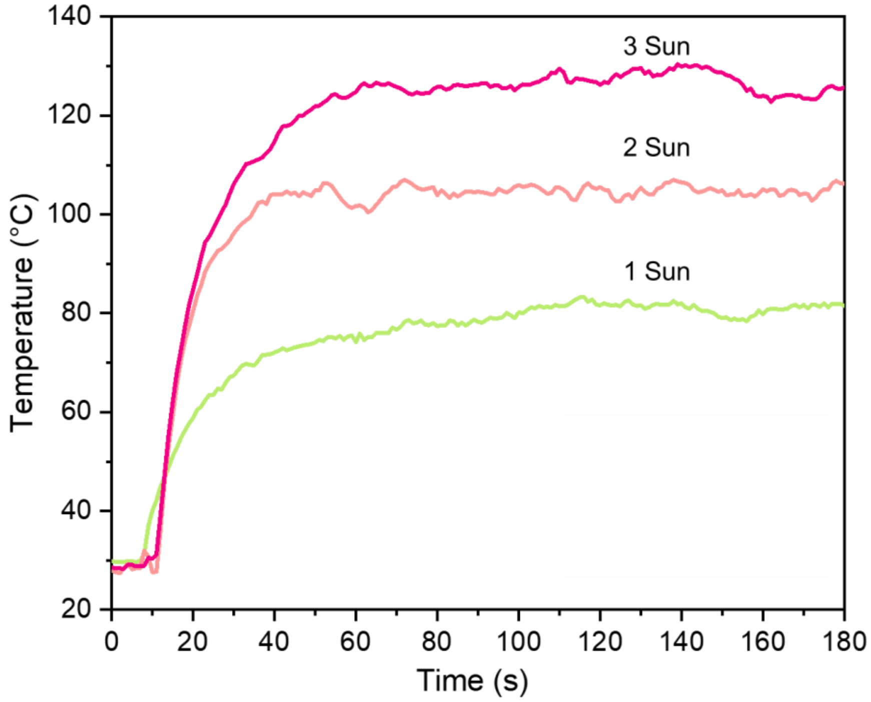

Figure S48. The temperature changes of TB-TCNQ@PU under 1 Sun, 2 Sun, and 3 Sun irradiation.

Water evaporation conversion efficiency Equation

The conversion efficiency (η) for solar to steam generation can be calculated as the following formula.[S22,S23]

$$\boldsymbol{\eta = \frac{mh_{LV}}{C_{opt}P_0}}$$

Where, m is the water evaporation rate under solar illumination, $\boldsymbol{h_{LV}}$ refers to the sensible heat and latent heat of total liquid-vapor phase-change, $\boldsymbol{P_0}$ is the solar irradiation density (1 kw·m^{-2}), $\boldsymbol{C_{opt}}$ represents the optical concentration, usually 1.

Where m is obtained from the following formula: $\boldsymbol{m = m_{light} - m_{black}}$

$\boldsymbol{m_{light}}$ refers to the water evaporation rate of samples irradiated by sunlight in a certain time period, while $\boldsymbol{m_{blank}}$ refers to the evaporation rate of water in the same time. In addition, the formula for $\boldsymbol{h_{LV}}$ is as follows:

$$\boldsymbol{h_{LV} = Q + \Delta h_{vap}}$$

Where $\boldsymbol{Q}$ is the energy that heats the system from its initial temperature($\boldsymbol{T_0}$) to its final temperature($\boldsymbol{T}$), and $\boldsymbol{\Delta h_{vap}}$ is the latent heat of vaporization of water. In order to further obtain the temperature change energy $\boldsymbol{Q}$ of the system, the specific heat capacity parameter $\boldsymbol{C_{liquid}}$ of liquid water is introduced, and its relationship with $\boldsymbol{Q}$ is as follows:

$$\boldsymbol{Q = C_{liquid} \times (T - T_0)}$$

In this paper, $\boldsymbol{C_{liquid}}$ is the heat capacity of liquid water of 4.18 J/(g·°C), In addition, due to the law of energy conservation, the formula for $\boldsymbol{\Delta h_{vap}}$ is as follows:

$$\boldsymbol{\Delta h_{vap} = Q_1 + \Delta h_{100} + Q_2}$$

Where $\boldsymbol{Q_1}$ refers to the energy released by liquid water when it rises from temperature $\boldsymbol{T}$ to 100 °C, $\boldsymbol{\Delta h_{100}}$ refers to the latent heat of vaporization of liquid water into water vapor at constant temperature, which is 2260 kJ/kg, and $\boldsymbol{Q_2}$ refers to the energy released when water vapor drops from 100 °C to temperature $\boldsymbol{T}$, At the same time, $\boldsymbol{Q_1}$ and $\boldsymbol{Q_2}$ can be obtained from the following equations:

$$\boldsymbol{Q_1 = C_{liquid} \times (100 - T)}$$

$$\boldsymbol{Q_2 = C_{vapor} \times (T - 100)}$$

In this paper, $\boldsymbol{C_{vapor}}$ is the heat capacity of water vapor of 1.865 J/(g·°C).

For TB-TCNQ@PU 4.24 mg cm^{-3}:

$\boldsymbol{T}$ = 60.23 °C, $\boldsymbol{T_0}$=28.34°C, $\mathbf{m}_{light}$ = 1.746 kg·m^{-2}·h^{-1}, $\mathbf{m}_{blank}$=0.386 kg m^{-2} h^{-1}.

$\boldsymbol{\eta}$=93.85%

Table S6. Comparison of solar-driven evaporation performance of TB-TCNQ@PU with other cutting-edge solar evaporators.

Type	PTC materials	Evaporation rate (kg m^{-2} h^{-1})	Evaporation efficiency (%)	Ref.
**Inclusion co-crystals**	**TB-TCNQ**	**1.746**	**93.8**	**This work**
2D semiconductor	Eu-doped SnSe nanosheets	2.17	96.5	[S24]
2D semiconductor	Pr-doped Bi2Se3 nanosheets	1.669	91.5	[S25]
Organic polymer	Polypyrrole Nanosheets	1.38	92	[S17]
MOF-isolated graphene	CoTCPP-Bi	1.43	98.5	[S26]
Cage-stabilized gold nanoparticles	AuNCs@TBN–Superphane	2.35	95.1	[S18]
MOF-isolated graphene	G@ZIF	1.78	>99.9	[S19]
Liquid Metal	LM/PAN	2.66	96.5	[S27]
Radical cations co-crystal	$ABTS^{+\bullet}$	1.407	97.0	[S28]
Carbon nanotube modified with polydopamine	ISWG	1.2	81.8	[S29]
Polymer	Polypyrrole Origamis	2.12	91.5	[S30]
Hydrogel	D-SH	2.6	91	[S31]
Covalent organic frameworks	GT-COF-3-loaded foam	1.414	90.7	[S32]
Charge-transfer complex	CTCC-S	1.67	90.3	[S33]
Polyaniline coated MOFs	CBAP/PVDF	1.866	90.8	[S34]

6. The optimized structure coordinates

***race*-TB[2]**

O	8.380904	6.8006	14.17717
O	8.814842	4.038708	10.19631
O	9.376303	-0.58268	13.04476
O	14.08583	-0.83365	14.25523
N	10.84598	5.629654	22.18291
N	9.689787	3.514945	22.23171
C	7.886133	7.894754	13.41891
H	8.629046	8.253904	12.68832
H	7.680755	8.69052	14.14313
H	6.952697	7.6364	12.89272
C	8.411808	5.106294	9.350134
H	7.390627	5.448809	9.584669
H	8.429069	4.704387	8.331228
H	9.105126	5.960489	9.415326
C	8.733275	5.645329	13.53276
C	8.600984	5.483351	12.14721
H	8.226278	6.301105	11.54354
C	8.933648	4.26445	11.54106
C	9.419179	3.188054	12.30792
C	9.548419	3.391318	13.67904

H	9.952666	2.575608	14.27352
C	9.21677	4.588473	14.33565
C	9.775036	1.858515	11.65321
H	8.876925	1.229374	11.58656
H	10.08554	2.051774	10.61728
C	10.87982	1.102821	12.36676
C	10.65943	-0.11269	13.041
C	11.72475	-0.78681	13.66449
H	11.5411	-1.71429	14.1935
C	9.09899	-1.83717	13.66211
H	9.658143	-2.65488	13.17931
H	8.025378	-2.00401	13.52235
H	9.332378	-1.82641	14.73706
C	13.01756	-0.24614	13.63401
C	13.26999	0.978612	12.9752
C	15.78601	0.87509	12.60717
H	15.70975	-0.19352	12.42695
C	14.61046	1.613751	12.92537
C	14.73509	2.978803	13.1346
H	13.85681	3.576382	13.3716
C	12.17996	1.610425	12.36189
H	12.362	2.54403	11.83044
C	13.94338	-2.1474	14.7858
H	13.23038	-2.1793	15.62306
H	14.93901	-2.42997	15.14534
H	13.62083	-2.86184	14.01157
C	9.352181	4.67205	15.81081
C	9.791858	5.850031	16.48333
H	10.01508	6.737783	15.90116
C	9.947911	5.882186	17.8544
H	10.28159	6.808487	18.31733
C	9.692962	4.739194	18.66324
C	9.259971	3.547472	17.9962
C	9.088362	3.55332	16.59119
H	8.721053	2.642337	16.12026
C	9.016691	2.376952	18.76532
H	8.68178	1.471937	18.25841
C	9.16994	2.391099	20.13082
H	8.94716	1.507442	20.7269
C	9.585077	3.572453	20.8038
C	9.873221	4.730111	20.08232
C	10.41191	5.958356	20.80924
H	11.26837	6.377506	20.26858
H	9.649626	6.752883	20.86446
C	9.777199	4.855583	22.81438
H	8.826025	5.384416	22.67385
H	9.972029	4.759845	23.88978
C	12.08989	4.911739	22.19403
C	12.15837	3.545364	22.4534

C	10.87574	2.752613	22.67665
H	10.90182	1.803797	22.1283
H	10.74236	2.4941	23.7397
C	13.26883	5.652594	21.90339
H	13.1794	6.72149	21.72027
C	14.49418	5.036185	21.86045
H	15.3919	5.608628	21.6352
C	14.6097	3.640654	22.12065
C	13.43069	2.890176	22.43998
C	13.58196	1.492857	22.67657
H	12.71809	0.886996	22.93865
O	22.21539	6.800628	21.00367
O	21.78182	4.038925	24.98471
O	21.21933	-0.58243	22.13678
O	16.50976	-0.83309	20.92643
N	19.74977	5.629119	12.99814
N	20.90596	3.514396	12.9495
C	22.71066	7.894669	21.76177
H	21.96803	8.254053	22.49254
H	22.91608	8.690354	21.03748
H	23.64415	7.636069	22.28774
C	22.18516	5.106478	25.83078
H	23.20635	5.448846	25.59605
H	22.16804	4.704593	26.8497
H	21.49195	5.960763	25.7657
C	21.86293	5.645461	21.64822
C	21.99553	5.483486	23.03374
H	22.37057	6.30117	23.6373
C	21.66276	4.26466	23.63998
C	21.17689	3.188316	22.87326
C	21.04747	3.391533	21.50215
H	20.64294	2.575904	20.90776
C	21.37915	4.588639	20.84546
C	20.82083	1.858918	23.52814
H	21.71886	1.229684	23.59493
H	20.5103	2.052372	24.56403
C	19.71597	1.10325	22.81468
C	19.93625	-0.11233	22.14054
C	18.87085	-0.78641	21.5171
H	19.05441	-1.71394	20.98814
C	21.4965	-1.83701	21.51955
H	20.93726	-2.65461	22.00244
H	22.5701	-2.00395	21.65932
H	21.26311	-1.82635	20.4446
C	17.57809	-0.24565	21.54761
C	17.32574	0.979135	22.20641
C	14.80975	0.875678	22.57448
H	14.886	-0.19291	22.75481
C	15.98529	1.614324	22.25626

C	15.86068	2.979345	22.04685
H	16.73894	3.576859	21.80964
C	18.41585	1.610924	22.81961
H	18.23388	2.544535	23.35108
C	16.65208	-2.14686	20.39588
H	17.36503	-2.17884	19.55856
H	15.65641	-2.42935	20.03641
H	16.97462	-2.86131	21.1701
C	21.2437	4.672109	19.3703
C	20.80401	5.850022	18.69766
H	20.58087	6.737864	19.27974
C	20.64794	5.882052	17.3266
H	20.31428	6.808318	16.86358
C	20.90289	4.738984	16.51786
C	21.33588	3.547323	17.185
C	21.50752	3.553307	18.59002
H	21.87487	2.64238	19.06103
C	21.57916	2.376739	16.41599
H	21.91412	1.471778	16.92297
C	21.42588	2.390757	15.05049
H	21.64865	1.507046	14.45449
C	21.01072	3.572043	14.3774
C	20.72259	4.729771	15.09879
C	20.18388	5.957944	14.37177
H	19.32744	6.377162	14.91241
H	20.94617	6.752463	14.31646
C	20.81853	4.854984	12.36671
H	21.76971	5.38383	12.50716
H	20.62367	4.759148	11.29133
C	18.50585	4.911195	12.98714
C	18.43738	3.544794	12.7279
C	19.71999	2.75203	12.50467
H	19.69395	1.803245	13.05308
H	19.85333	2.493452	11.44164
C	17.32692	5.652084	13.27772
H	17.41636	6.721001	13.46071
C	16.10157	5.03568	13.32077
H	15.20383	5.608132	13.54598
C	15.98607	3.640115	13.06076
C	17.16506	2.889605	12.74147
C	17.01378	1.49227	12.50501
H	17.87764	0.886395	12.24294
N	12.78842	5.731579	14.49014
N	13.76372	6.732513	18.65967
N	10.83098	-2.065	16.90117
N	11.56484	-0.885	21.08748
C	11.20136	-1.25708	17.65598
C	11.6598	-0.26856	18.57289
C	11.61612	-0.61736	19.95406

C	12.06467	0.997099	18.15113
C	12.53055	1.975463	19.1085
H	12.63171	1.689474	20.15066
C	12.85414	3.234477	18.72105
H	13.1965	3.95765	19.45729
C	12.74059	3.632118	17.33458
C	12.33578	2.633694	16.36567
H	12.27943	2.905632	15.31525
C	12.02322	1.371565	16.75555
H	11.71306	0.639364	16.0139
C	13.00869	4.938612	16.94715
C	12.88502	5.372815	15.59421
C	13.4223	5.930171	17.88715
N	17.80771	5.731642	20.69097
N	16.83221	6.732372	16.5213
N	19.76456	-2.06515	18.28066
N	19.03089	-0.88544	14.09423
C	19.39426	-1.25728	17.52577
C	18.93595	-0.26878	16.60877
C	18.97958	-0.61769	15.22762
C	18.53121	0.996967	17.0304
C	18.06542	1.975275	16.07295
H	17.96429	1.689233	15.0308
C	17.74186	3.234342	16.46025
H	17.39956	3.957459	15.72393
C	17.85543	3.632134	17.84667
C	18.26023	2.633786	18.81567
H	18.31661	2.905854	19.86606
C	18.5727	1.371589	18.42593
H	18.88285	0.639477	19.16767
C	17.58736	4.938697	18.23396
C	17.71111	5.372905	19.5869
C	17.17366	5.930105	17.29389

TCNQ

N	17.80771	5.731642	20.69097
N	16.83221	6.732372	16.5213
N	19.76456	-2.06515	18.28066
N	19.03089	-0.88544	14.09423
C	19.39426	-1.25728	17.52577
C	18.93595	-0.26878	16.60877
C	18.97958	-0.61769	15.22762
C	18.53121	0.996967	17.0304
C	18.06542	1.975275	16.07295
H	17.96429	1.689233	15.0308
C	17.74186	3.234342	16.46025
H	17.39956	3.957459	15.72393
C	17.85543	3.632134	17.84667

C	18.26023	2.633786	18.81567
H	18.31661	2.905854	19.86606
C	18.5727	1.371589	18.42593
H	18.88285	0.639477	19.16767
C	17.58736	4.938697	18.23396
C	17.71111	5.372905	19.5869
C	17.17366	5.930105	17.29389

TB-TCNQ

O	8.380904	6.8006	14.17717
O	8.814842	4.038708	10.19631
O	9.376303	-0.58268	13.04476
O	14.08583	-0.83365	14.25523
N	10.84598	5.629654	22.18291
N	9.689787	3.514945	22.23171
C	7.886133	7.894754	13.41891
H	8.629046	8.253904	12.68832
H	7.680755	8.69052	14.14313
H	6.952697	7.6364	12.89272
C	8.411808	5.106294	9.350134
H	7.390627	5.448809	9.584669
H	8.429069	4.704387	8.331228
H	9.105126	5.960489	9.415326
C	8.733275	5.645329	13.53276
C	8.600984	5.483351	12.14721
H	8.226278	6.301105	11.54354
C	8.933648	4.26445	11.54106
C	9.419179	3.188054	12.30792
C	9.548419	3.391318	13.67904
H	9.952666	2.575608	14.27352
C	9.21677	4.588473	14.33565
C	9.775036	1.858515	11.65321
H	8.876925	1.229374	11.58656
H	10.08554	2.051774	10.61728
C	10.87982	1.102821	12.36676
C	10.65943	-0.11269	13.041
C	11.72475	-0.78681	13.66449
H	11.5411	-1.71429	14.1935
C	9.09899	-1.83717	13.66211
H	9.658143	-2.65488	13.17931
H	8.025378	-2.00401	13.52235
H	9.332378	-1.82641	14.73706
C	13.01756	-0.24614	13.63401
C	13.26999	0.978612	12.9752
C	15.78601	0.87509	12.60717
H	15.70975	-0.19352	12.42695
C	14.61046	1.613751	12.92537
C	14.73509	2.978803	13.1346

H	13.85681	3.576382	13.3716
C	12.17996	1.610425	12.36189
H	12.362	2.54403	11.83044
C	13.94338	-2.1474	14.7858
H	13.23038	-2.1793	15.62306
H	14.93901	-2.42997	15.14534
H	13.62083	-2.86184	14.01157
C	9.352181	4.67205	15.81081
C	9.791858	5.850031	16.48333
H	10.01508	6.737783	15.90116
C	9.947911	5.882186	17.8544
H	10.28159	6.808487	18.31733
C	9.692962	4.739194	18.66324
C	9.259971	3.547472	17.9962
C	9.088362	3.55332	16.59119
H	8.721053	2.642337	16.12026
C	9.016691	2.376952	18.76532
H	8.68178	1.471937	18.25841
C	9.16994	2.391099	20.13082
H	8.94716	1.507442	20.7269
C	9.585077	3.572453	20.8038
C	9.873221	4.730111	20.08232
C	10.41191	5.958356	20.80924
H	11.26837	6.377506	20.26858
H	9.649626	6.752883	20.86446
C	9.777199	4.855583	22.81438
H	8.826025	5.384416	22.67385
H	9.972029	4.759845	23.88978
C	12.08989	4.911739	22.19403
C	12.15837	3.545364	22.4534
C	10.87574	2.752613	22.67665
H	10.90182	1.803797	22.1283
H	10.74236	2.4941	23.7397
C	13.26883	5.652594	21.90339
H	13.1794	6.72149	21.72027
C	14.49418	5.036185	21.86045
H	15.3919	5.608628	21.6352
C	14.6097	3.640654	22.12065
C	13.43069	2.890176	22.43998
C	13.58196	1.492857	22.67657
H	12.71809	0.886996	22.93865
O	22.21539	6.800628	21.00367
O	21.78182	4.038925	24.98471
O	21.21933	-0.58243	22.13678
O	16.50976	-0.83309	20.92643
N	19.74977	5.629119	12.99814
N	20.90596	3.514396	12.9495
C	22.71066	7.894669	21.76177
H	21.96803	8.254053	22.49254

H	22.91608	8.690354	21.03748
H	23.64415	7.636069	22.28774
C	22.18516	5.106478	25.83078
H	23.20635	5.448846	25.59605
H	22.16804	4.704593	26.8497
H	21.49195	5.960763	25.7657
C	21.86293	5.645461	21.64822
C	21.99553	5.483486	23.03374
H	22.37057	6.30117	23.6373
C	21.66276	4.26466	23.63998
C	21.17689	3.188316	22.87326
C	21.04747	3.391533	21.50215
H	20.64294	2.575904	20.90776
C	21.37915	4.588639	20.84546
C	20.82083	1.858918	23.52814
H	21.71886	1.229684	23.59493
H	20.5103	2.052372	24.56403
C	19.71597	1.10325	22.81468
C	19.93625	-0.11233	22.14054
C	18.87085	-0.78641	21.5171
H	19.05441	-1.71394	20.98814
C	21.4965	-1.83701	21.51955
H	20.93726	-2.65461	22.00244
H	22.5701	-2.00395	21.65932
H	21.26311	-1.82635	20.4446
C	17.57809	-0.24565	21.54761
C	17.32574	0.979135	22.20641
C	14.80975	0.875678	22.57448
H	14.886	-0.19291	22.75481
C	15.98529	1.614324	22.25626
C	15.86068	2.979345	22.04685
H	16.73894	3.576859	21.80964
C	18.41585	1.610924	22.81961
H	18.23388	2.544535	23.35108
C	16.65208	-2.14686	20.39588
H	17.36503	-2.17884	19.55856
H	15.65641	-2.42935	20.03641
H	16.97462	-2.86131	21.1701
C	21.2437	4.672109	19.3703
C	20.80401	5.850022	18.69766
H	20.58087	6.737864	19.27974
C	20.64794	5.882052	17.3266
H	20.31428	6.808318	16.86358
C	20.90289	4.738984	16.51786
C	21.33588	3.547323	17.185
C	21.50752	3.553307	18.59002
H	21.87487	2.64238	19.06103
C	21.57916	2.376739	16.41599
H	21.91412	1.471778	16.92297

C	21.42588	2.390757	15.05049
H	21.64865	1.507046	14.45449
C	21.01072	3.572043	14.3774
C	20.72259	4.729771	15.09879
C	20.18388	5.957944	14.37177
H	19.32744	6.377162	14.91241
H	20.94617	6.752463	14.31646
C	20.81853	4.854984	12.36671
H	21.76971	5.38383	12.50716
H	20.62367	4.759148	11.29133
C	18.50585	4.911195	12.98714
C	18.43738	3.544794	12.7279
C	19.71999	2.75203	12.50467
H	19.69395	1.803245	13.05308
H	19.85333	2.493452	11.44164
C	17.32692	5.652084	13.27772
H	17.41636	6.721001	13.46071
C	16.10157	5.03568	13.32077
H	15.20383	5.608132	13.54598
C	15.98607	3.640115	13.06076
C	17.16506	2.889605	12.74147
C	17.01378	1.49227	12.50501
H	17.87764	0.886395	12.24294
N	12.78842	5.731579	14.49014
N	13.76372	6.732513	18.65967
N	10.83098	-2.065	16.90117
N	11.56484	-0.885	21.08748
C	11.20136	-1.25708	17.65598
C	11.6598	-0.26856	18.57289
C	11.61612	-0.61736	19.95406
C	12.06467	0.997099	18.15113
C	12.53055	1.975463	19.1085
H	12.63171	1.689474	20.15066
C	12.85414	3.234477	18.72105
H	13.1965	3.95765	19.45729
C	12.74059	3.632118	17.33458
C	12.33578	2.633694	16.36567
H	12.27943	2.905632	15.31525
C	12.02322	1.371565	16.75555
H	11.71306	0.639364	16.0139
C	13.00869	4.938612	16.94715
C	12.88502	5.372815	15.59421
C	13.4223	5.930171	17.88715
N	17.80771	5.731642	20.69097
N	16.83221	6.732372	16.5213
N	19.76456	-2.06515	18.28066
N	19.03089	-0.88544	14.09423
C	19.39426	-1.25728	17.52577
C	18.93595	-0.26878	16.60877

C	18.97958	-0.61769	15.22762
C	18.53121	0.996967	17.0304
C	18.06542	1.975275	16.07295
H	17.96429	1.689233	15.0308
C	17.74186	3.234342	16.46025
H	17.39956	3.957459	15.72393
C	17.85543	3.632134	17.84667
C	18.26023	2.633786	18.81567
H	18.31661	2.905854	19.86606
C	18.5727	1.371589	18.42593
H	18.88285	0.639477	19.16767
C	17.58736	4.938697	18.23396
C	17.71111	5.372905	19.5869
C	17.17366	5.930105	17.29389

7. References

[S1] Y. D. Zhao, J. Han, Y. Chen, Y. Su, Y. M. Cao, B. Wu, S. M. Yu, M.-D. Li, Z. Wang, M. Zheng, M.-P. Zhuo, L.-S. Liao, Organic Charge-Transfer Cocrystals toward Large-Area Nanofiber Membrane for Photothermal Conversion and Imaging. *ACS Nano* **2022**, *16*, 15000.

[S2] Y. Wang, W. Zhu, W. Du, X. Liu, X. Zhang, H. Dong, W. Hu, Cocrystals Strategy towards Materials for Near-Infrared Photothermal Conversion and Imaging, *Angew. Chem. Int. Ed.* **2018**, *57*, 3963.

[S3] Y. D. Zhao, J. Han, Y. Chen, Y. Su, Y. M. Cao, B. Wu, S. M. Yu, M.-D. Li, Z. Wang, M. Zheng, M.-P. Zhuo, L.-S. Liao, Organic Charge-Transfer Cocrystals toward Large-Area Nanofiber Membrane for Photothermal Conversion and Imaging, *ACS Nano* **2022**, *16*, 15000.

[S4] S. Tian, Z. Huang, J. Tan, X. Cui, Y. Xiao, Y. Wan, X. Li, Q. Zhao, S. Li, C.-S. Lee, Manipulating Interfacial Charge-Transfer Absorption of Cocrystal Absorber for Efficient Solar Seawater Desalination and Water Purification, *ACS Energy Lett.* **2020**, *5*, 2698.

[S5] Y.-T. Chen, X. Wen, J. He, Z. Li, S. Zhu, W. Chen, J. Yu, Y. Guo, S. Ni, S. Chen, L. Dang, M.-D. Li, Boosting Near-Infrared Photothermal Conversion by Intermolecular Interactions in Isomeric Cocrystals, *ACS Appl. Mater. Interfaces* **2022**, *14*, 28781.

[S6] P. Shi, X.-X. Liu, X.-L. Dai, T.-B. Lu, J.-M. Chen, Near-infrared photothermal conversion properties of carbazole-based cocrystals with different degrees of charge transfer, *CrystEngComm* **2022**, *24*, 4622.

[S7] C. Ou, W. Na, W. Ge, H. Huang, F. Gao, L. Zhong, Y. Zhao, X. Dong, Biodegradable Charge-Transfer Complexes for Glutathione Depletion Induced Ferroptosis and NIR-II Photoacoustic Imaging Guided Cancer Photothermal Therapy, *Angew. Chem. Int. Ed.* **2021**, *60*, 8157.

[S8] W. Chen, S. Sun, G. Huang, S. Ni, L. Xu, L. Dang, D. L. Phillips, M.-D. Li, Unprecedented Improvement of Near-Infrared Photothermal Conversion Efficiency to 87.2% by Ultrafast Non-radiative Decay of Excited States of Self-Assembly Cocrystal, *J. Phys. Chem. Lett.* **2021**, *12*, 5796.

[S9] J. Xu, W. Chen, S. Li, Q. Chen, T. Wang, Y. Shi, S. Deng, M. Li, P. Wei, Z. Chen, Organic stoichiometric cocrystals with a subtle balance of charge-transfer degree and molecular stacking towards high-efficiency NIR photothermal conversion, *Chin. Chem. Lett*. **2024**, *35*, 109808.

[S10] D. Zhang, S. Li, S. Gao, S. Fu, K. Liu, D. He, H. Liu, X. Zhang, and W. Hu. NIR-II Organic Photothermal Cocrystals with Strong Charge Transfer Interaction for Flexible Wearable Heaters. *Chin. J. Chem.* **2024**, *42*, 1563.

[S11] J. Xu, Q. Chen, S. Li, J. Shen, P. Keoingthong, L. Zhang, Z. Yin, X. Cai, Z. Chen, W. Tan, Charge-Transfer Cocrystal via a Persistent Radical Cation Acceptor for Efficient Solar-Thermal Conversion, *Angew. Chem., Int. Ed.* **2022**, *61*, e202202571.

[S12] H. Xiang, Q. Yang, Y. Gao, D. Zhu, S. Pan, T. Xu, Y. Chen, Cocrystal Strategy toward Multifunctional 3D-Printing Scaffolds Enables NIR-Activated Photonic Osteosarcoma Hyperthermia and Enhanced Bone Defect Regeneration, *Adv. Funct. Mater.* **2020**, *30*, 1909938.

[S13] D. Wang, W. Wang, H. Lu, C. You, L. Liang, C. Liu, H. Xiang, Y. Chen, Charge transfer of ZnTPP/C60 cocrystal-hybridized bioimplants satisfies osteosarcoma eradication with antitumoral, antibacterial and osteogenic performances, *Nano Today.* **2022**, *46*, 101562.

[S14] S. Tian, H. Bai, S. Li, Y. Xiao, X. Cui, X. Li, J. Tan, Z. Huang, D. Shen, W. Liu, P. Wang, B. Z. Tang, C.-S. Lee, Water-Soluble Organic Nanoparticles with Programable Intermolecular Charge Transfer for NIR-II Photothermal Anti-Bacterial Therapy, *Angew. Chem., Int. Ed.* **2021**, *60*, 11758.

[S15] Y.-T. Chen, W. Chen, J. He, G. Zhang, X. Wen, S. Ran, Z. Deng, S. Zhu, H. Li, S. Ni, S. Chen, L. Dang, M.-D. Li, Tailormade Nonradiative Rotation Tuning of the Near-Infrared Photothermal Conversion in Donor–Acceptor Cocrystals, *J. Phys. Chem. C.* **2021**, *125*, 25462.

[S16] W. Wang, X. Yan, J. Geng, N. Zhao, L. Liu, T. Vogel, Q. Guo, L. Ge, B. Luo, and Y. Zhao, Engineering a Copper@Polypyrrole Nanowire Network in the Near Field for Plasmon-Enhanced Solar Evaporation. *ACS Nano.* **2021**, *15*, 16376.

[S17] X. Wang, Q. Liu, S. Wu, B. Xu, H. Xu, Multilayer Polypyrrole Nanosheets with Self-Organized Surface Structures for Flexible and Efficient Solar–Thermal Energy Conversion. *Adv. Mater*. **2019**, *31*, 1807716.

[S18] Y. Zhang, J. Zhou, K. Luo, W. Zhou, F. Wang, J. Li, and Q. He, Ferritin-Inspired Encapsulation and Stabilization of Gold Nanoclusters for High-Performance Photothermal Conversion. *Angew. Chem. Int. Ed*. **2025**, e202500058.

[S19] X. Han, L. V. Besteiro, C. S. L. Koh, H. K. Lee, I. Y. Phang, G. C. Phan-Quang, J. Y. Ng, H. Y. F. Sim, C. L. Lay, A. Govorov, X. Y. Ling, Intensifying Heat Using MOF-Isolated Graphene for Solar-Driven Seawater Desalination at 98% Solar-to-Thermal Efficiency. *Adv. Funct. Mater.* **2021**, *31*, 2008904.

[S20] Z. Jiang, C. Zhang, X. Wang, M. Yan, Z. Ling, Y. Chen, Z. Liu, A Borondifluoride-Complex-Based Photothermal Agent with an 80% Photothermal Conversion Efficiency for Photothermal Therapy in the NIR-II Window. *Angew. Chem. Int. Ed*. **2021**, *60*, 22376.

[S21] X. Yan, S. Lyu, X.-Q. Xu, W. Chen, P. Shang, Z. Yang, G. Zhang, W. Chen, Y. Wang, L. Chen, Superhydrophilic 2D Covalent Organic Frameworks as Broadband Absorbers for Efficient Solar Steam Generation. *Angew. Chem. Int. Ed*. **2022**, *61*, e202201900.

[S22] M. Gao, L. Zhu, C. K. Peh, G. W. Ho, Solar Absorber Material and System Designs for Photothermal Water Vaporization towards Clean Water and Energy Production *Energy Environ. Sci.* **2019**, *12*, 841.

[S23] F. Zhao, X. Zhou, Y. Shi, X. Qian, M. Alexander, X. Zhao, S. Mendez, R. Yang, L. Qu, G. Yu, Solar Absorber Material and System Designs for Photothermal Water Vaporization towards Clean Water and Energy Production. *Nat. Nanotech*. **2018**, *13*, 489.

[S24] J. Hu, X. Yao, K. Han, Y. Ge, S. Xu, G. Bai, Boosted Near-Infrared Photothermal Conversion in Rare Earth Ions-Doped 2D SnSe Nanosheets for Solar-Powered Water Evaporation Systems. *Small* **2024**, *20*, 2405742.

[S25] Y. Huang, G. Bai, Y. Zhao, Y. Liu, S. Xu, J. Hao, Lanthanide-Doped Topological Nanosheets with Enhanced Near Infrared Photothermal Performance for Energy Conversion. *ACS Appl. Mater. Interfaces* **2021**, *13*, 43094–43103.

[S26] L. He, J. He, E.-X. Chen and Q. Lin, Boosting photothermal conversion through array aggregation of metalloporphyrins in bismuth-based coordination frameworks. *Chem. Sci.*, **2024**, *15*, 17498.

[S27] Y. Sun, D. Liu, F. Zhang, X. Gao, J. Xue, Q. Zheng, Multiscale Biomimetic Evaporators Based on Liquid Metal/Polyacrylonitrile Composite Fibers for Highly Efficient Solar Steam Generation. *Nano-Micro Letters* **2025**, *17*, 129.

[S28] J. Xu, Q. Chen, S. Li, J. Shen, P. Keoingthong, L. Zhang, Z. Yin, X. Cai, Z. Chen, W. Tan, Charge-Transfer Cocrystal via a Persistent Radical Cation Acceptor for Efficient Solar-Thermal Conversion. *Angew. Chem. Int. Ed.* **2022**, *61*, e202202571.

[S29] B. Jin, Y. Lu, X. Zhang, X. Zhang, D. Li, Q. Liu, B. Deng, H. Li. Iceberg-inspired solar water generator for enhanced thermoelectricity–freshwater synergistic production. *Chem. Eng. J.* **2023**, *469*,143906.

[S30] W. Li, Z. Li, K. Bertelsmann, D. E. Fan, Portable Low-Pressure Solar Steaming-Collection Unisystem with Polypyrrole Origamis. *Adv. Mater.* **2019**, *31*, 1900720.

[S31] Y. Guo, F. Zhao, X. Zhou, Z. Chen, G. Yu, Tailoring Nanoscale Surface Topography of Hydrogel for Efficient Solar Vapor Generation. *Nano Lett.* **2019**, *19*, 2530–2536.

[S32] X. Tang, Z. Chen, Q. Xu, Y. Su, H. Xu, S. Horike, H. Zhang, Y. Li, C. Gu, Design of Photothermal Covalent Organic Frame works by Radical Immobilization. *CCS Chem.* **2022**, *4*, 2842–2853.

[S33] S. Tian, Z. Huang, J. Tan, X. Cui, Y. Xiao, Y. Wan, X. Li, Q. Zhao, S. Li, C. S. Lee, Manipulating Interfacial Charge-Transfer Absorption of Cocrystal Absorber for Efficient Solar Seawater Desalination and Water Purification. *ACS Energy Lett.* **2020**, *5*, 2698–2705.

[S34] Z. Li, X. Ma, D. Chen, X. Wan, X. Wang, Z. Fang, X. Peng, Polyaniline-Coated MOFs Nanorod Arrays for Efficient Evaporation-Driven Electricity Generation and Solar Steam Desalination. *Adv. Sci.* **2021**, *8*, 2004552.
